# Financial toxicity on treatment outcomes in head & neck cancer patients undergoing radiation therapy

**DOI:** 10.1186/s13014-025-02749-x

**Published:** 2025-11-25

**Authors:** Garrett K. Harada, Eric Ku, Jino Park, Akul Munjal, Nicholas Peterson, Sophie Hsu, Rupali Banker, Shirin Attarian, Erin Healy, Michael Hoyt, Gelareh Sadigh, Allen Chen, Jeremy P. Harris

**Affiliations:** 1 Department of Radiation Oncology, University of California Irvine, Orange, CA USA; 2 Department of Radiation Oncology, Sutter Memorial Medical Center, Modesto, CA USA; 3 Department of Hematology/Oncology, University of California Irvine, Orange, CA USA; 4 Department of Population Health and Disease Prevention, University of California Irvine, Irvine, CA USA; 5 Department of Radiological Sciences, University of California Irvine, Orange, CA USA; 6 Department of Radiation Oncology, University of California Irvine, Chao Family Comprehensive Cancer Center, 101 The City Drive South, Building 23, Orange, CA 92868 USA

**Keywords:** Financial toxicity, FACIT, Radiation therapy, Head and neck cancer, HRQoL, QLQ-C30, CTCAE

## Abstract

**Background:**

Financial toxicity, defined as hardship from medical costs, is an emerging concept in healthcare. Here we define financial toxicity in head and neck cancer patients receiving radiation, identify risk factors, and determine associations with HRQoL, treatment morbidity, and survival.

**Methods:**

We conducted a prospective study on consecutive patients referred to a tertiary referral center for radiation therapy for head and neck malignancies (July 2021–June 2023). Patients provided consent and were assessed using validated patient-reported outcome measures for financial toxicity (FACIT-COST), HRQoL (EORTC-QLQ-C30), and symptom burden (PRO-CTCAE) before and after radiation therapy. Primary outcomes included two-year overall survival (OS), treatment morbidity (ER visits, hospitalizations, feeding tube placement, missed radiation days), HRQoL, and symptom burden.

**Results:**

Among 74 patients (median age 69), all completed pre-radiation therapy (pre-RT) measures, and 39 completed post-RT measures. Median pre-RT COST was 29 (range: 0–44), with 41.9% scoring ≤25, indicating worse financial toxicity. Lower pre-RT COST scores correlated with younger age, Black race, Medicaid insurance, single or unemployed status, advanced T-stage, and concurrent chemoradiotherapy (*p* < 0.05). These patients had worse HRQoL, more severe symptoms, increased feeding tube placements, and more ER visits/hospitalizations (*p* < 0.05). OS was worse with lower pre- (HR = 0.95; 95% CI = 0.91–0.99; *p* = 0.015) and post-RT COST scores (HR = 0.92; 95% CI = 0.86–0.98; *p* = 0.012).

**Conclusions:**

Financial toxicity is common in head and neck radiation patients and linked to worse HRQoL, morbidity, and OS. Affected patients had clear socioeconomic risk factors and advanced disease. Further research should explore interventions to improve cancer outcomes.

**Supplementary information:**

The online version contains supplementary material available at 10.1186/s13014-025-02749-x.

## Background

Primary malignancies of the head and neck are common in the United States, with roughly 12 cases per 100,000 people per year [1, 2]. Radiotherapy (RT) is frequently indicated in both the primary and adjuvant treatment of these cancers. However, head and neck RT is notoriously associated with treatment-related toxicity and morbidity, including pain, mucositis, desquamation, dysphagia, and weight loss. Consideration and management of these toxicities is challenging for treating providers, and often necessitates extensive multidisciplinary care with potentials for parenteral nutrition and treatment cessation. Such outcomes may lead to significant increases in treatment-related costs or financial toxicity, and may include out-of-pocket expenses, time away from work, transportation fees, and caregiving needs, which have been linked to worse health-related quality of life (HRQoL) and mortality [3, 4].

With a primary focus on symptom mitigation to facilitate completion of head and neck RT, the financial toxicity and associated impacts on HRQoL are poorly understood. Understanding such burdens are critical for these patients as advances in oncologic care have led to people surviving longer after head and neck cancer diagnoses, potentially left with crippling economic loss or bankruptcy [5–7]. Such financial toxicity may be further compounded by considerations of an aging population, development of expensive therapies, and projected inflation in costs for cancer patients [8]. In addition to socioeconomic indicators such as income and employment, race may independently be associated with financial toxicity. For instance, Black patients more often have less access to quality care, adequate screening, and are more likely to be financially hurt by cancer care compared to White patients [9].

The goal of this study was to determine the degree of financial toxicity in a population undergoing RT for primary head and neck cancer, as well as identify associations between financial toxicity and outcomes of overall survival, treatment morbidity, and HRQoL in this patient group.

## Methods

### Patients

This study included adult patients diagnosed with cancer who were referred to our NCI-designated Comprehensive Cancer Center for radiation therapy to the head and neck region between July 2021 and June 2023. Eligible patients were 18 years or older, required to complete baseline patient-reported outcome (PRO) measures (pre-RT), and must have initiated radiation therapy to the head and neck, here defined as receipt of at least one fraction of treatment. No incentives were provided for participation, however, given administration of PRO measures in English, patients were screened to verify English reading comprehension prior to questionnaire administration and subsequent study enrollment. Enrollment of non-English speaking patients was permitted. Enrolled patients were subsequently assessed using PRO measures following completion of radiation therapy (post-RT) administered at the date of the next scheduled post-RT follow-up. The timing of post-RT follow-up was left to the discretion of the treating radiation oncologist but would occur at 2 to 4 weeks or 10 to 12 weeks after treatment, based upon severity of acute toxicity. This study was thoroughly reviewed and approved by the institutional review board before commencement (IRB #2639).

### Measures

Financial toxicity was assessed with the Functional Assessment of Chronic Illness Therapy (FACIT) – Comprehensive Score Financial Toxicity (COST) (Version 2) 11-item questionnaire [10]. Scores range from 0 to 44 with lower scores indicating worse financial toxicity.

Treatment satisfaction was assessed with the FACIT – Treatment Satisfaction General (FACIT-TS-G) (Version 4) [11]. Quality of life was assessed with the European Organisation for Research and Treatment of Cancer Quality of Life Questionnaire (EORTC QLQ-C30) (Version 3) which includes domains in global, physical, role, emotional, cognitive, and social functioning [12]. Symptoms and treatment-related adverse effects were assessed with the Patient-Reported Outcomes version of the Common Terminology Criteria for Adverse Events (PRO-CTCAE) (Version 1) and included items assessing pain, fatigue, appetite, nausea, vomiting, shortness of breath, cough, wheezing, swallowing, voice quality, and hoarseness [13]. Self-reported measures and relevant scoring criterion can be found in (Supplemental File 1, 2 and 3).

Given prospective and ongoing enrollment, a date for interim analysis was arbitrarily set to 3 years after the first patient was enrolled on study. It was therefore expected that many patients would have not completed post-RT questionnaires for the present study. At that time, patient demographics, clinical information, disease characteristics, insurance status, socioeconomic factors, radiation treatment details, and survival outcomes were collected from electronic health records. Percutaneous endoscopic gastrostomy (PEG) tube placements, emergency room visits, and hospital admissions during or within 6 weeks of completion of RT were recorded. Median household income was defined by listed zip code using 2020 Census data [14].

### Statistical analyses

COST, FACIT-TS-G, EORTC-QLQ-C30 and PRO-CTCAE scores were calculated using previously published methods [15–18]. Incomplete COST questionnaire responses were accounted for using previously published methods [16].

Association of baseline categorical variables and continuous variables with COST were evaluated with univariate linear regression. Nonparametric evaluation was also performed using Spearman’s rank correlation coefficient and the Kruskal Wallis test. The Shapiro-Wilk test was used to assess data for normality followed by paired t-tests and Wilcoxon rank sum assessments to compare pre-RT and post-RT COST, EORTC QLQ-C30 domains, and PRO-CTCAE outcomes for parametric and non-parametric data, respectively.

Grading of financial toxicity was performed to stratify the cohort into two groups based on a previously proposed grading system – Grade 0 (score ≥ 26) and Grade 1+ (score ≤ 25) [19, 20]. Kaplan-Meier curves were used to estimate overall survival (OS) from time of RT start to death from any cause or date of final follow-up. The log-rank test was used to compare survival between groups. Univariate and multivariate Cox proportional hazards regression models were used to analyze associations between financial toxicity, clinical, and demographic factors with overall survival. Multivariate Cox model covariates were selected given assessed potential for confounding survival outcomes and included pre- versus post-RT COST scores, age, advanced T-stage, and treatment intent. For the multivariate Cox model inclusive of post-RT COST scores, the time from RT completion to survey administration (in days) was additionally included as a covariate due to observed relationship of post-RT COST score and survey administration date. The proportional hazards assumption was checked using Schoenfeld residuals, with non-proportional hazards assessed by creating an interaction term with the natural log of time. Data analysis was performed using Stata version 18.0 (StataCorp LLC, College Station, TX).

## Results

74 patients who underwent radiation therapy to the head and neck region and completed pre-RT PRO measures were included in this study. Of these patients, at the time of analysis, 39 patients had completed post-RT questionnaires. Of the 35 missing post-RT measures, 25 patients had yet to be seen for post-treatment follow-up, while the remaining 10 patients died prior. The median duration of follow-up was 16.7 months (range: 4.0 to 33.8 months). Baseline clinical, socioeconomic, and treatment details show in Table 1.

**Table 1:** Patient baseline demographics, disease characteristics, and treatment details

Characteristics (n = 74)	Median or Number (Percent)	Range
Age (years)	69	[20-95]
Female	20 (27%)	
Male	54 (73%)	
Race		
Asian	7 (10%)	
Black	4 (5%)	
White	52 (70%)	
Other/Unknown	11 (15%)	
English Preferred Language	56 (76%)	
Insurance		
Medicare	45 (64%)	
Medicaid	10 (14%)	
Area income ($ per year)	91,000	[21000] − 180,000]
Distance to treatment Center	14	[1.5 - 70]
Performance Score		
ECOG 0	11 (15%)	
ECOG 1	58 (78%)	
ECOG 2	5 (7%)	
Charlson Comorbidity Index (CCI)	5	[2–11]
Smoking Status		
Active	8 (11%)	
Former	26 (35%)	
Primary Treatment Site		
Oral Cavity	18 (24%)	
Oropharynx	3 (4%)	
Larynx/Hypopharynx	6 (8%)	
Nasopharynx	3 (4%)	
Sinonasal	6 (8%)	
Salivary Gland	3 (4%)	
Thyroid	3 (4%)	
Orbit	3 (4%)	
Cutaneous	25 (34%)	
Neck	4 (5%)	
Disease		
Stage 1	11 (15%)	
Stage 2	16 (22%)	
Stage 3	11 (15%)	
Stage 4	36 (49%)	
Recurrent Disease	14 (19%)	
Distant Metastases	6 (8%)	
Prior Surgery	45 (61%)	
Prior Systemic Therapy	8 (11%)	
Prior Radiation Therapy	6 (8%)	
Radiation Therapy		
Definitive Intent	69 (93%)	
Palliative Intent	5 (7%)	
Concurrent Chemotherapy	20 (27%)	
Total Dose (Gy)	60	[8-72]
No. of Fractions	30	[1-60]
BED10 Dose (Gy)	72	[13-84]
3D Conformal Technique	15 (20%)	
IMRT Technique	59 (80%)	

BED10 = Biologically effective dose using an α/β = 10 Gy; IMRT = Intensity modulated radiation therapy

Median age was 68.9 years old, 73% were male, 27% were female. The most common primary disease sites treated were cutaneous (33.8%), oral cavity (24.3%), and sinonasal (8.1%). Stage I disease was found in 14.9%, Stage 2 in 21.6%, Stage 3 in 14.9%, Stage 4 in 48.7%, and metastatic disease in 8.1%. 8.1% received prior courses of radiation therapy, 10.8% had prior chemotherapy, and 60.8% had prior surgery. ECOG scores were all 0 to 2 with most being 0 or 1 (93.2%). Median Charlson Comorbidity Index (CCI) was 5 (range 2 to 11). 32.4% had a greater than 10 pack-year smoking history while 10.8% were still actively smoking.

The majority of patients identified their race as white (70.3%) and listed English as their preferred language (75.7%). 63.5% had Medicare, 13.5% had Medicaid, and 23.0% had commercial insurance. 86.2% were either employed or retired. Median household income by listed zip-code was $91,000 (range: $42,000 to $183,000). Median distance to the cancer center was 14 miles (range: 1 to 70 miles).

Treatment plan was curative-intent chemoradiation in 27.0%, curative-intent radiation alone in 66.2%, and palliative-intent radiation alone in 6.8%. Median biologically effective dose using an α/β = 10 Gy (BED_α/β = 10 Gy_) was 72 Gy (range: 14 to 72 Gy). Intensity-modulated radiation therapy (IMRT) technique was used in 79.7%.

### Pre-RT financial toxicity

Median pre-RT COST was 29 (range: 0 to 44) with lower scores indicating worse financial toxicity − 58.1% had grade 0 (score ≥ 26) and 41.9% had grade 1 or higher (score 14–25) financial toxicity.

Baseline demographic, cancer, and treatment factors associated with lower pre-RT COST were younger age, black race, Medicaid insurance, being single, unemployed, advanced T-stage, and receipt of concurrent chemoradiotherapy (*p* < 0.05, for all). In contrast, older (*p* = 0.001), widowed patients (*p* = 0.020) with cutaneous head and neck malignancies (*p* = 0.002) had less financial toxicity. Non-parametric evaluation also identified significant association with financial toxicity and increasing radiation dose (Rho = −0.247; *p* = 0.035) and number of fractions delivered (Rho = −0.262, *p* = 0.024)(Table 2).

**Table 2:** Univariate regression & Spearman’s Rank correlation coefficient for demographic & disease characteristics with pre- and post-RT COST score

	Pre-RT COST			Post-RT COST			Pre-RT COST		Post-RT COST	
	Coeff	95% CI	p	Coeff	95% CI	p	Rho	p	Rho	p
**Baseline Characteristics**										
Age (years)	**0.26**	**(0.11 - 0.41)**	**0.001**	**0.30**	**(0.09 - 0.52)**	**0.007**	**0.382**	** < 0.001**	**0.459**	**0.004**
Female (vs Male)	4.84	(−0.75 - 10.43)	0.089	**9.40**	**(1.23 - 17.57)**	**0.025**	0.204	0.082	**0.319**	**0.048**
Race								**0.030**		0.264
Asian	4.21	(−4.39 - 12.81)	0.333	4.13	(−10.14 - 18.40)	0.561				
Black	**−15.09**	**(−5.71 - −4.46)**	**0.006**	5.22	(−9.02 - 19.45)	0.462				
White	5.04	(−0.38 - 10.45)	0.068	3.68	(−4.50 - 11.87)	0.368				
Other/Unknown	−5.07	(−12.09 - 1.95)	0.155	**−11.13**	**(−21.05 - −1.21)**	**0.029**				
Marital Status								** < 0.001**		0.272
Single	**−10.89**	**(−16.34 - −5.44)**	** < 0.001**	−7.37	(−16.10 - 1.35)	0.095				
Married	3.43	(−1.64 - 8.50)	0.182	4.03	(−3.71 - 11.76)	0.299				
Widowed	**9.39**	**(1.54 - 17.24)**	**0.020**	8.41	(−8.68 - 25.50)	0.325				
Divorced	2.51	(−6.75 - 11.77)	0.591	−0.58	(−13.17 - 12.01)	0.926				
English Preferred Language (vs Non-English)	5.23	(−0.54 - 11.00)	0.075	**10.29**	**(0.94 - 19.64)**	**0.032**	0.211	0.071	**0.333**	**0.038**
Employment Status								**0.006**		0.050
Employed	0.95	(−4.77 - 6.68)	0.741	2.83	(−5.71 - 11.36)	0.506				
Retired	**5.52**	**(0.17 - 10.87)**	**0.044**	5.15	(−2.76 - 13.06)	0.195				
Unemployed	**−13.42**	**(−20.68 - −6.16)**	** < 0.001**	**−12.21**	**(−21.52 - −2.90)**	**0.012**				
Insurance								**0.002**		0.206
Medicare	4.97	(−0.16 - 10.10)	0.057	5.01	(−2.78 - 12.80)	0.201				
Medicaid	**−13.94**	**(−20.59 - −7.30)**	** < 0.001**	−5.87	(−18.31 - 6.57)	0.345				
Other Insurance	2.69	(−3.29 - 8.68)	0.373	−3.21	(−11.89 - 5.48)	0.459				
Area income ($ per year)	0.04	(−0.05 - 0.14)	0.401	0.09	(−0.04 - 0.21)	0.180	0.089	0.451	0.184	0.260
Distance to treatment Center	0.06	(−0.16 - 0.28)	0.582	0.16	(−0.31 - 0.64)	0.488	0.054	0.646	0.071	0.667
Performance Score							−0.136	0.250	−0.106	0.521
ECOG 0	4.73	(−2.30 - 11.76)	0.184	1.65	(−8.29 - 11.59)	0.739				
ECOG 1	−3.32	(−9.42 - 2.78)	0.282	0.52	(−8.55 - 9.58)	0.909				
ECOG 2	−0.57	(−10.67 - 9.52)	0.911	−6.87	(−24.04 - 10.30)	0.423				
Date of Survey Administration Prior to RT (Days)	0.01	(−0.03 - 0.04)	0.783				0.04	0.711		
Date of Survey Administration After RT (Days)				0.04	(−0.00 - 0.08)	0.058			**0.37**	**0.039**
**Disease Characteristics**										
Primary Treatment Site								0.101		0.789
Oral Cavity	−3.31	(−9.16 - 2.55)	0.264	0.70	(−8.04 - 9.45)	0.871				
Oropharynx	−12.35	(−24.86 - 0.16)	0.053	−0.02	(−17.34 - 17.30)	0.998				
Larynx/Hypopharynx	−1.50	(−10.78 - 7.77)	0.748	−9.77	(−33.72 - 14.18)	0.414				
Nasopharynx	−4.74	(−17.53 - 8.05)	0.463	−3.71	(−20.99 - 13.57)	0.666				
Sinonasal	−1.89	(−11.16 - 7.38)	0.685	1.28	(−13.05 - 15.61)	0.857				
Salivary Gland	0.12	(−12.72 - 12.97)	0.985	3.67	(−13.61 - 20.95)	0.670				
Thyroid	−5.09	(−17.87 - 7.70)	0.430	−20.03	(−42.26 - 3.20)	0.089				
Orbit	4.64	(−8.16 - 17.44)	0.472	10.76	(−13.15 - 34.66)	0.368				
Cutaneous	**8.23**	**(3.23 - 13.22)**	**0.002**	1.91	(−6.03 - 9.85)	0.629				
Neck	−5.89	(−17.01 - 5.22)	0.294	−2.61	(−16.92 - 11.70)	0.714				
T Stage										
T3-4 (vs T1-2)	**−6.54**	**(−12.25 - − 0.82)**	**0.026**	−6.37	(−14.83 - 2.08)	0.134	**−0.322**	**0.015**	−0.319	0.081
Stage							**−0.315**	**0.007**	**−0.430**	**0.007**
Stage 1	3.40	(−3.67 - 10.48)	0.341	6.71	(−3.64 - 17.06)	0.197				
Stage 2	**6.77**	**(0.82 - 12.71)**	**0.026**	7.50	(−0.88 - 15.89)	0.078				
Stage 3	0.21	(−6.91 - 7.33)	0.953	−2.78	(−13.33 - 7.77)	0.596				
Stage 4	**−6.42**	**(−11.26 - −1.58)**	**0.010**	**−7.90**	**(−15.14 - −0.66)**	**0.033**				
Recurrent Disease	−3.27	(−9.70 - 3.15)	0.313	**−10.02**	**(−19.40 - −0.64)**	**0.037**	−0.133	0.258	**−0.366**	**0.022**
Distant Metastatic Recurrence	−6.24	(−15.40 - 2.93)	0.179	−8.75	(−22.79 - 5.29)	0.215	−0.172	0.146	−0.214	0.198
Prior Surgery	3.66	(−1.45 - 8.78)	0.158	−5.94	(−13.45 - 1.57)	0.117	0.154	0.191	−0.274	0.092
Prior Systemic Therapy	−0.17	(−2.53 - 2.19)	0.888	−5.39	(−16.68 - 5.90)	0.339	−0.184	0.117	−0.188	0.256
Prior RT	−5.29	(−14.49 - 3.90)	0.255	−11.76	(−25.55 - 2.04)	0.092	−0.160	0.176	−0.261	0.110
Radiation Therapy										
Definitive Intent (vs Palliative)	−0.99	(−11.08 - 9.09)	0.845	−5.66	(−29.76 - 18.43)	0.637	−0.035	0.770	−0.072	0.744
Concurrent Chemotherapy	**−7.43**	**(−12.86 - −2.00)**	**0.008**	−4.89	(−13.49 - 3.70)	0.256	**−0.302**	**0.009**	−0.220	0.179
Total Dose (Gy)	−0.11	(−0.29 - 0.07)	0.212	−0.12	(−0.52 - 0.28)	0.532	**−0.247**	**0.035**	−0.192	0.240
No. of Fractions	−0.16	(−0.40 - 0.08)	0.199	−0.14	(−0.57 - 0.28)	0.499	**−0.231**	**0.048**	−0.152	0.354
BED10 Dose (Gy)	−0.11	(−0.27 - 0.05)	0.175	−0.16	(−0.54 - 0.23)	0.414	**−0.262**	**0.024**	−0.249	0.126
3D Conformal Technique (vs IMRT)	5.25	(−0.93 - 11.43)	0.095	7.11	(−3.21 - 17.43)	0.171	−0.215	0.066	−0.246	0.132

Results for univariate linear regressions, Spearmann’s Rho, and Kruskal-Wallis tests are shown for associations between baseline patient and disease characteristics with pre-radiation therapy (pre-RT) and post-radiation therapy (post-RT) COST scores. Bolded values indicate statistical significance at *p* < 0.05. Coeff = Coefficient; CI = Confidence interval; BED10 = Biologically effective dose using an α/β = 10 Gy; IMRT = Intensity modulated radiation therapy

Lower pre-RT COST was also associated with lower pre-RT EORTC QLQ-C30 global health (*p* = 0.001), role (*p* = 0.019), and emotional functioning (*p* = 0.042) scores. Social functioning demonstrated an inverse relationship with pre-RT COST scores, such that financial toxicity was more likely in patients with few disruptions to family or social interactions (*p* = 0.048). Baseline symptoms of pain, fatigue, nausea, shortness of breath, cough, swallowing dysfunction, voice changes, and hoarseness were also associated with worse pre-RT financial toxicity (*p* < 0.05, for all)(Table 3).

**Table 3:** Univariate regression & Spearman’s rank correlation coefficient for patient-reported outcomes & pre- and post-RT COST score

	Pre-COST			Post-COST			Pre-COST		Post-COST	
	Coeff	95% CI	p	Coeff	95% CI	p	Rho	p	Rho	p
**Patient-Reported Outcomes**										
Pre-Treatment EORTC-QLQ-C30										
Global Functioning	**0.18**	**(0.08 - 0.28)**	**0.001**	**0.22**	**(0.06 - 0.38)**	**0.008**	**0.405**	** < 0.001**	**0.462**	**0.003**
Role Functioning	**0.09**	**(0.02 - 0.16)**	**0.019**	**0.17**	**(0.05 - 0.28)**	**0.007**	**0.308**	**0.009**	**0.474**	**0.004**
Cognitive Functioning	0.09	(−0.28 - 0.21)	0.132	0.12	(−0.06 - 0.30)	0.170	0.224	0.062	0.252	0.144
Physical Functioning	0.07	(−0.03 - 0.16)	0.163	**0.24**	**(0.08 - 0.40)**	**0.005**	**0.270**	**0.023**	**0.532**	**0.001**
Emotional Functioning	**0.15**	**(0.07 - 0.24)**	**0.042**	**0.17**	**(0.06 - 0.29)**	**0.003**	**0.367**	**0.002**	**0.485**	**0.003**
Social Functioning	**−2.34**	**(−4.65 - −0.02)**	**0.048**	**−4.91**	**(−8.43 - −1.39)**	**0.008**	**−0.251**	**0.035**	**−0.412**	**0.013**
Post-Treatment EORTC-QLQ-C30										
Global Functioning	0.12	(−0.03 - 0.26)	0.108	**0.18**	**(0.04 - 0.31)**	**0.013**	0.299	0.065	**0.420**	**0.008**
Role Functioning	**0.16**	**(0.04 - 0.28)**	**0.012**	**0.19**	**(0.07 - 0.31)**	**0.002**	**0.393**	**0.017**	**0.413**	**0.012**
Cognitive Functioning	0.10	(−0.05 - 0.26)	0.181	0.13	(−0.02 - 0.29)	0.088	0.312	0.061	0.274	0.101
Physical Functioning	0.16	(−0.004 - 0.33)	0.055	**0.19**	**(0.003 - 0.37)**	**0.047**	0.307	0.078	0.324	0.062
Emotional Functioning	**0.25**	**(0.11 - 0.40)**	**0.001**	**0.27**	**(0.13 - 0.42)**	** < 0.001**	**0.559**	** < 0.001**	**0.547**	**0.001**
Social Functioning	**−4.72**	**(−8.16 - −1.28)**	**0.009**	**−4.75**	**(−8.17 - −1.33)**	**0.008**	**−0.441**	**0.007**	**−0.453**	**0.005**
FACIT-TS-G	0.31	(−0.01 - 0.62)	0.057	**0.81**	**(0.39 - 1.23)**	**0.001**	**0.291**	**0.037**	**0.640**	**0.001**
Pre-Treatment PRO CTCAE										
Pain										
Often	**−3.25**	**(−5.12 - −1.38)**	**0.001**	**−4.28**	**(−7.17 - −1.39)**	**0.005**	**−0.407**	** < 0.001**	**−0.460**	**0.005**
Severe	**−3.04**	**(−4.94 - −1.14)**	**0.002**	**−5.01**	**(−7.84 - −2.19)**	**0.001**	**−0.379**	**0.001**	**−0.544**	** < 0.001**
Interfere	**−3.78**	**(−5.74 - −1.81)**	** < 0.001**	**−4.55**	**(−7.88 - −1.22)**	**0.009**	**−0.445**	** < 0.001**	**−0.431**	**0.012**
Fatigue										
Severe	**−2.91**	**(−4.90 - −0.92)**	**0.005**	**−3.55**	**(−6.61 - −0.48)**	**0.025**	**−0.312**	**0.009**	**−0.381**	**0.025**
Interfere	**−2.97**	**(−4.70 - −1.24)**	**0.001**	**−3.37**	**(−6.06 - −0.67)**	**0.016**	**−0.366**	**0.002**	**−0.419**	**0.012**
Appetite										
Severe	−1.86	(−4.06 - 0.33)	0.095	−2.23	(−5.62 - 1.16)	0.190	−0.179	0.140	−0.301	0.079
Interfere	−2.37	(−4.78 - 0.04)	0.054	−2.14	(−5.55 - 1.27)	0.210	**−0.261**	**0.030**	**−0.333**	**0.048**
Nausea										
Often	**−3.80**	**(−6.93 - −0.67)**	**0.018**	−3.38	(−7.64 - 0.88)	0.116	**−0.245**	**0.042**	−0.275	0.104
Severe	**−3.38**	**(−6.11 - −0.64)**	**0.016**	−3.47	(−7.22 - 0.28)	0.069	−0.201	0.097	−0.325	0.054
Vomiting										
Often	−2.88	(−6.55 - 0.80)	0.124	−3.86	(−8.93 - 1.21)	0.131	−0.180	0.135	**−0.331**	**0.048**
Severe	−2.51	(−5.85 - 0.83)	0.139	−3.57	(−8.80 - 1.65)	0.174	−0.204	0.098	−0.284	0.099
Shortness of Breath										
Severe	−2.70	(−5.82 - 0.42)	0.089	−2.78	(−8.96 - 3.39)	0.365	**−0.255**	**0.036**	−0.168	0.340
Interfere	**−3.49**	**(−6.11 - −0.87)**	**0.010**	−3.23	(−7.46 - 0.99)	0.129	**−0.297**	**0.014**	−0.200	0.250
Cough										
Severe	**−3.01**	**(−5.35 - −0.66)**	**0.013**	−1.92	(−5.45 - 1.61)	0.277	**−0.320**	**0.008**	−0.259	0.133
Interfere	−2.17	(−5.11 - 0.78)	0.147	−1.87	(−6.32 - 2.58)	0.399	**−0.253**	**0.037**	−0.230	0.192
Wheezing										
Severe	−1.98	(−5.73 - 1.78)	0.296	−1.78	(−9.81 - 6.25)	0.654	−0.189	0.124	−0.139	0.429
Swallowing										
Severe	**−3.50**	**(−5.41 - −1.59)**	**0.001**	**−3.36**	**(−5.96 - −0.76)**	**0.013**	**−0.405**	** < 0.001**	**−0.526**	**0.001**
Voice Changes	**−5.93**	**(−10.33 - −1.53)**	**0.009**	**−13.2**	**(−21.29 - −5.12)**	**0.002**	**−0.387**	**0.001**	**−0.533**	**0.001**
Hoarseness										
Severe	**−2.94**	**(−4.87 - −1.02)**	**0.003**	**−4.94**	**(−7.72 - −2.15)**	**0.001**	**−0.412**	** < 0.001**	**0.571**	** < 0.001**
Post-Treatment PRO CTCAE										
Pain										
Often	**−4.82**	**(−7.88 - −1.75)**	**0.003**	**−4.52**	**(−7.66 - −1.38)**	**0.006**	**−0.487**	**0.003**	**−0.426**	**0.011**
Severe	−2.85	(−6.19 - 0.49)	0.092	−2.86	(−6.23 - 0.51)	0.094	−0.296	0.084	−0.233	0.177
Interfere	−2.56	(−6.44 - 1.32)	0.187	−3.46	(−7.46 - 0.55)	0.088	−0.295	0.128	−0.306	0.113
Fatigue										
Severe	**−4.13**	**(−7.49 - −0.77)**	**0.017**	**−3.76**	**(−7.14 - −0.38)**	**0.030**	**−0.404**	**0.015**	**−0.356**	**0.034**
Interfere	**−4.01**	**(−6.77 - −1.25)**	**0.006**	**−3.25**	**(−6.16 - −0.34)**	**0.030**	**−0.426**	**0.010**	**−0.335**	**0.046**
Appetite										
Severe	−2.02	(−5.15 - 1.11)	0.199	−2.31	(−5.37 - 0.75)	0.134	−0.232	0.192	−0.268	0.131
Interfere	**−3.17**	**(−6.22 - −0.12)**	**0.042**	−2.45	(−5.55 - 0.65)	0.117	**−0.362**	**0.033**	−0.252	0.144
Nausea										
Often	−3.67	(−8.47 - 1.12)	0.129	0.36	(−4.58 - 5.30)	0.884	−0.187	0.269	0.057	0.735
Severe	**−5.37**	**(−9.50 - −1.24)**	**0.012**	−1.28	(−5.76 - 3.19)	0.564	**−0.345**	**0.040**	−0.070	0.682
Vomiting										
Often	**−6.16**	**(−11.81 - −0.51)**	**0.033**	−1.41	(−7.44 - 4.61)	0.636	**−0.358**	**0.035**	−0.140	0.421
Severe	**−5.42**	**(−10.57 - −0.28)**	**0.039**	−2.12	(−7.50 - 3.27)	0.429	**−0.375**	**0.024**	−0.191	0.266
Shortness of Breath										
Severe	−5.12	(−10.48 - 0.25)	0.061	**−5.66**	**(−10.90 - −0.42)**	**0.035**	−0.216	0.211	−0.211	0.223
Interfere	−3.56	(−8.76 - 1.64)	0.173	−4.12	(−9.36 - 1.13)	0.120	−0.297	0.083	−0.279	0.104
Cough										
Severe	−1.02	(−4.51 - 2.47)	0.556	0.28	(−3.19- 3.76)	0.870	−0.159	0.360	−0.065	0.710
Interfere	−1.35	(−4.62 - 1.92)	0.407	0.14	(−3.22 - 3.50)	0.933	−0.148	0.396	−0.011	−0.951
Wheezing										
Severe	1.48	(−3.41 - 6.36)	0.543	−0.42	(−5.29 - 4.45)	0.862	−0.053	0.758	−0.174	0.309
Swallowing										
Severe	**−2.97**	**(−5.43 - −0.50)**	**0.020**	**−2.64**	**(−5.13 - −0.16)**	**0.038**	**−0.449**	**0.007**	**−0.440**	**0.008**
Voice Changes	−1.08	(−10.04 - 7.89)	0.808	−1.50	(−10.15 - 7.16)	0.727	−0.064	0.717	−0.100	0.573
Hoarseness										
Severe	−1.03	(−4.95 - 2.89)	0.596	−2.33	(−6.15 - 1.49)	0.223	−0.189	0.268	−0.328	0.051

Results for univariate linear regressions and Spearmann’s Rho tests are shown for associations between patient-reported outcomes with pre-radiation therapy (pre-RT) and post-radiation therapy (post-RT) COST scores. PRO CTCAE questionnaire items are assessed by quantifying how often a given symptom arises, interferes with daily life, or the severity over the past 7 days. Bolded values indicate statistical significance at *p* < 0.05.Coeff = Coefficient; CI = Confidence interval

### Post-RT financial toxicity

**Fig. 1:**
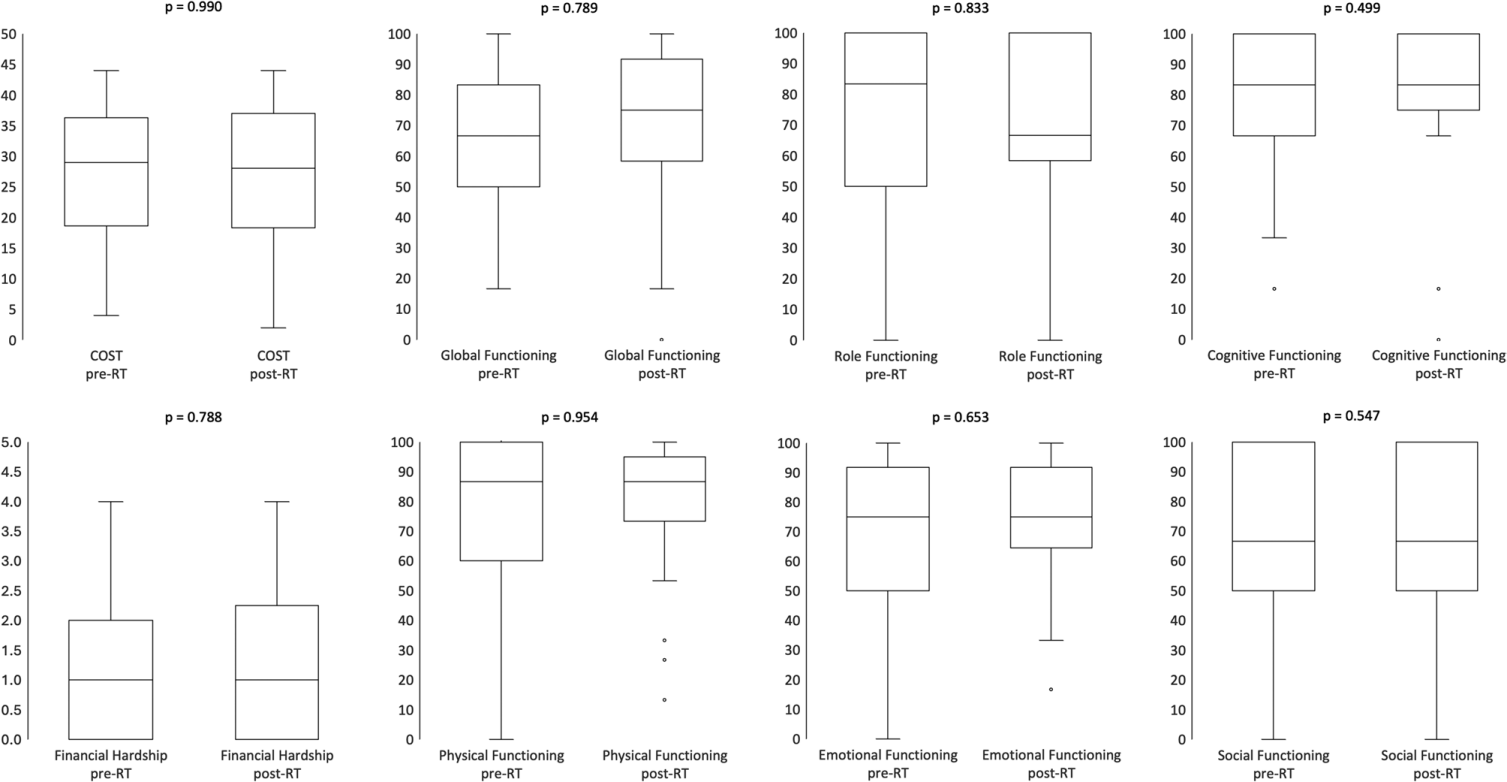
Distribution of pre-radiation therapy (pre-RT) and post-radiation therapy (post-RT) COST, financial hardship (COST) and QOL functioning scores (EORTC QLQ-C30). Lower COST indicates higher financial toxicity, higher financial hardship indicates higher toxicity, higher treatment satisfaction and EORTC functional scales indicate improved quality of life. P-values displayed indicate either two-sided t-tests or wilcoxon rank sum tests for parametric and non-parametric comparisons between pre- and post-RT questionnaires

Of the 39 patients that completed both pre-RT and post-RT questionnaires, no significant differences were found between post-RT and pre-RT PRO scores. Scores are shown in (Fig. 1) & (Supplemental Fig. 1). 16 patients had a decrease in COST, reflecting worsened financial toxicity, with a mean decrease of 7.5 (72% of baseline COST SD).

Lower post-RT COST scores were similarly seen in younger (*p* = 0.007) and unemployed patients (*p* = 0.012), but also included non-English speaking patients (*p* = 0.032) with distant metastatic disease recurrences (*p* = 0.037). In contrast to pre-RT COST, there was no longer any association with cutaneous head and neck malignancies or treatment dose and fractionation. (Table 2) Higher baseline (*p* = 0.005) and post-treatment physical function (*p* = 0.047) was associated with less reported financial toxicity. Similarly, those expressing greater treatment satisfaction on the FACIT-TS-G also had higher post-RT COST scores (*p* = 0.001) (Table 3).

### Post-treatment morbidity & survival outcomes

2-year overall survival, measured from time of RT start, was 68.2% for the entire group. 60.8% of patients missed at least 1 day of scheduled RT. 18.9% needed PEG tubes placed and 23.0% either presented to the ED or were hospitalized during or within 6 weeks after completion of RT. PEG tube placement (Coeff = −10.07; 95% CI = −16.09 – −4.06; *p* = 0.001) and ED visits or hospitalizations (Coeff = −9.86; 95% CI = −16.90 - −2.82; *p* = 0.007) were significantly associated with lower pre-RT COST scores. Further, there was a trend toward missing one or more scheduled days of RT with worsening post-treatment financial toxicity (Coeff = − 7.04; 95% CI = −14.38–0.30; *p* = 0.060).

Overall survival was observed to be worse with more severe pre- (HR = 0.95; 95% CI = 0.91–0.99; *p* = 0.015) and post-RT financial toxicity (HR = 0.92; 95% CI = 0.86–0.98; *p* = 0.012). Of the 31 patients identified with pre-RT grade 1 or higher financial toxicity (31/74 = 41.9%), 2-year OS was 41.5% while those without financial toxicity had a 2-year OS of 83.7% (*p* = 0.004). Similar findings were noted in the 18 patients with post-RT grade 1 or higher financial toxicity (18/39 = 46.2%) (*p* = 0.002). (Supplemental Figure 2) Of patients treated with curative intent, 85.0% (34/40) and 65.5% (19/29) of patients without and with grade 1 or higher financial toxicity remained alive across the study interval. On multivariate Cox regression, pre- and post-RT COST scores remained associated with OS despite controlling for palliative intent, advanced T-stage disease, patient age, and timing of survey administration (for post-RT COST only). Worse baseline and post-treatment patient-reported outcomes were also associated with an increased hazard for death and are shown in (Table 4).

**Table 4:** Cox proportional hazards regression analysis of baseline demographics, disease characteristics, treatment details, and financial toxicity with overall survival

Baseline Characteristics	HR	95% CI	p	Pre-Treatment Patient-Reported Outcomes	HR	95% CI	p	Post-Treatment Patient-Reported Outcomes	HR	95% CI	p
Age (years)	1.02	(0.98 - 1.05)	0.356	Pre-Treatment COST	**0.95**	**(0.91 - 0.99)**	**0.015**	Post-Treatment COST	**0.92**	**(0.86 - 0.98)**	**0.012**
Female (vs Male)	0.15	(0.02 - 1.09)	0.061								
Race				Pre-Treatment EORTC-QLQ-C30				Post-Treatment EORTC-QLQ-C30			
Asian	0.66	(0.09 - 5.11)	0.691	Global Functioning	**0.98**	**(0.96 - 0.99)**	**0.010**	Global Functioning	**0.97**	**(0.95 - 1.00)**	**0.045**
Black	1.09	(0.14 - 8.46)	0.934	Role Functioning*	**0.97**	**(0.95 - 1.00)**	**0.025**	Role Functioning	**0.96**	**(0.94 - 0.98)**	**0.001**
White	Ref			Cognitive Functioning*	**0.94**	**(0.89 - 0.98)**	**0.010**	Cognitive Functioning	0.99	(0.97 - 1.01)	0.200
Other/Unknown	2.01	(0.64 - 6.37)	0.233	Physical Functioning*	**0.97**	**(0.93 - 1.00)**	**0.034**	Physical Functioning	**0.97**	**(0.94 - 0.99)**	**0.016**
English Preferred Language (vs Non-English)	1.05	(0.34 - 3.22)	0.934	Emotional Functioning*	**0.96**	**(0.92 - 0.99)**	**0.015**	Emotional Functioning	**0.97**	**(0.95 - 1.00)**	**0.030**
Insurance				Social Functioning*	**0.95**	**(0.92 - 0.99)**	**0.008**	Social Functioning	**2.36**	**(1.08 - 5.16)**	**0.031**
Medicare	Ref										
Medicaid	1.3	(0.37 - 4.60)	0.688					FACIT-TS-G	**1.01**	**(0.95 - 1.09)**	**0.700**
Other Insurance	0.4	(0.09 - 1.77)	0.225								
Area income ($ per year)	1	(1.00 - 1.00)	0.317	Pre-Treatment PRO CTCAE				Post-Treatment PRO CTCAE			
Distance to treatment Center	0.99	(0.95 - 1.04)	0.732	Pain				Pain			
Performance Score				Often	**1.70**	**(1.16 - 2.48)**	**0.007**	Often	**2.20**	**(1.09 - 4.43)**	**0.028**
ECOG 0	Ref			Severe	**1.62**	**(1.13 - 2.33)**	**0.009**	Severe	1.87	(0.93 - 3.77)	0.080
ECOG 1	3.19	(0.42 - 24.39)	0.264	Interfere*	**2.66**	**(1.15 - 6.17)**	**0.023**	Interfere	1.98	(0.97 - 4.04)	0.062
ECOG 2	9.15	(0.93 - 90.35)	0.058	Fatigue				Fatigue			
Disease Characteristics				Severe*	**3.34**	**(1.35 - 8.28)**	**0.009**	Severe	**2.44**	**(1.33 - 4.48)**	**0.004**
Primary Treatment Site				Interfere*	**3.84**	**(1.44 - 10.25)**	**0.007**	Interfere	1.47	(0.88 - 2.46)	0.142
Oral Cavity	Ref			Appetite				Appetite			
Oropharynx	0.47	(0.06 - 3.78)	0.474	Severe	1.22	(0.83 - 1.80)	0.320	Severe	1.72	(0.96 - 3.07)	0.066
Larynx/Hypopharynx	-	-	-	Interfere	1.37	(0.95 - 1.98)	0.088	Interfere	1.42	(0.85 - 2.37)	0.176
Nasopharynx	0.47	(0.06 - 3.89)	0.487	Nausea				Nausea			
Sinonasal	0.91	(0.19 - 4.31)	0.902	Often	1.53	(0.98 - 2.38)	0.062	Often	1.96	(0.87 - 4.42)	0.104
Salivary Gland	-	-	-	Severe	1.44	(0.99 - 2.09)	0.054	Severe	**2.00**	**(1.04 - 3.84)**	**0.037**
Thyroid	0.83	(0.10 - 6.74)	0.859	Vomiting				Vomiting			
Orbit	-	-	-	Often	1.37	(0.89 - 2.10)	0.150	Often	1.74	(0.76 - 3.98)	0.187
Cutaneous	**0.17**	**(0.05 - 0.66)**	**0.010**	Severe	1.37	(0.92 - 2.05)	0.123	Severe	1.77	(0.81 - 3.87)	0.154
Neck	0.50	(0.06 - 4.06)	0.521	Shortness of Breath				Shortness of Breath			
T Stage				Severe	1.42	(0.84 - 2.40)	0.185	Severe	**3.19**	**(1.46 - 7.01)**	**0.004**
T3-4 (vs T1-2)	1.95	(0.61 - 6.24)	0.261	Interfere	**1.68**	**(1.13 - 2.49)**	**0.010**	Interfere	**3.55**	**(1.50 - 8.40)**	**0.004**
Stage				Cough				Cough			
Stage 1	Ref			Severe	1.19	(0.85 - 1.67)	0.301	Severe	0.89	(0.43 - 1.85)	0.754
Stage 2	1.35	(0.25 - 7.41)	0.727	Interfere	1.33	(0.91 - 1.96)	0.140	Interfere	0.87	(0.42 - 1.79)	0.708
Stage 3	1.24	(0.17 - 8.89)	0.831	Wheezing				Wheezing			
Stage 4	2.09	(0.45 - 9.65)	0.345	Severe	1.17	(0.69 - 1.97)	0.564	Severe	0.77	(0.23 - 2.57)	0.670
Recurrent Disease	2.29	(0.80 - 6.54)	0.123	Swallowing				Swallowing			
Distant Metastatic Recurrence	1.53	(0.35 - 6.72)	0.570	Severe	1.27	(0.93 - 1.74)	0.128	Severe	1.33	(0.88 - 2.00)	0.180
Prior Surgery	0.68	(0.26 - 1.76)	0.423	Voice Changes	1.92	(0.98 - 3.76)	0.057	Voice Changes	1.83	(0.42 - 7.95)	0.421
Prior Systemic Therapy	1.10	(0.76 - 1.57)	0.622	Hoarseness				Hoarseness			
Prior RT	2.40	(0.69 - 8.39)	0.171	Severe	1.31	(0.95 - 1.81)	0.106	Severe	1.42	(0.78 - 2.59)	0.252
Radiation Therapy											
Definitive Intent (vs Palliative)	0.53	(0.12 - 2.34)	0.403	Multivariate Cox Regression				Multivariate Cox Regression			
Concurrent Chemotherapy	1.08	(0.38 - 3.03)	0.889	Age*	**1.03**	**(1.00 - 1.05)**	**0.048**	Age*	1.00	(0.94 - 1.06)	0.953
Total Dose (Gy)	1.03	(0.99 - 1.08)	0.176	T3-4 (vs T1-2)	1.89	(0.54 - 6.57)	0.319	T3-4 (vs T1-2)*	1.24	(0.42 - 3.62)	0.687
No. of Fractions	1.02	(0.98 - 1.07)	0.268	Definitive Intent (vs Palliative)	1.08	(0.19 - 6.08)	0.931	Definitive Intent (vs Palliative)	0.11	(0.00 - 8.17)	0.313
BED10 Dose (Gy)	1.04	(0.99 - 1.10)	0.105	Pre-Treatment COST	**0.92**	**(0.87 - 0.98)**	**0.008**	Post-Treatment COST	**25.84**	**(1.02 - 654.36)**	**0.049**
3D Conformal Technique (vs IMRT)	0.45	(0.10 - 1.94)	0.281					Time to Survey Administration After RT (Days)	0.98	(0.96 - 1.01)	0.161

Results for univariate and multivariate Cox proportional hazards models for overall survival are displayed for all collected patient, disease, and patient-reported outcome characteristics. Proportional hazards assumptions were checked using Schoenfeld residuals, with non-proportional covariates interacted with the natural log of time. Bolded values indicate statistical significance at *p* < 0.05

* = Non-proportional hazard; Ref = Reference group; BED10 = Biologically effective dose using an α/β = 10 Gy; IMRT = Intensity modulated radiation therapy

## Discussion

Financial toxicity is an understudied concept as it relates to RT for head and neck malignancies. Here, measured financial toxicity before and after RT was associated with worse HRQoL, treatment-related, and survival outcomes. Such patients were typically those with larger primary disease or certain socioeconomic risk factors: younger age, Medicaid insurance coverage, and lacking employment. Moreover, most patients in this study were treated with curative intent, raising concerns over the long-term implications of financial toxicity. These findings highlight critical considerations of patient socioeconomic diversity and emphasizes a need to better identify financial hardship both before and after RT for head and neck cancers, and could provide a potentially modifiable risk factor for cancer-related outcomes.

Since initial publication of the COST score by de Souza et al., multiple studies have independently validated its use in radiation oncology populations [3, 6, 21–24]. While the burden of financial toxicity in the head and neck cancer population has been reported, data are lacking evaluating financial toxicity of these patients receiving radiation and potential care/outcomes implications [25]. Other studies measuring COST scores in radiation therapy cohorts site similarities in risk factors for financial toxicity, such as race, marital status, employment status, education level, income level, medication cost, insurance status, and previous treatment type (e.g. chemotherapy, surgery) [21, 24]. While these results make it clear that financial toxicity is multifactorial, nuances between cancer type and specific treatments may explain small differences in reported risk factors. Specifically, nearly all studies report potential burdens of patient age and loss or change of employment, while fewer cite factors of previous treatment or disease stage which may be more specific to a given diagnosis [3, 21, 24, 26–28]. Such differences may further drive or suggest increased costs of care for standard-of-care therapy or symptom management. For instance, in 2019, the annual patient cost of oral cavity cancer care was estimated to be $56,400 at time of diagnosis followed by $5,700 annually thereafter [7]. With costs of cancer care only projected to increase in the future, such results highlight the particular vulnerabilities of head and neck cancer patients to financial toxicity and stresses the importance of identifying means to mitigate expenses for these patients [8].

Optimization of financial toxicity may also be of benefit prior to initiation of RT for head and neck cancers, as patients with worse baseline financial toxicity were more likely to require PEG tube placement and require emergency room visits or hospitalizations. While previous studies have examined pre-treatment COST scores on cancer-related outcomes, none explicitly examined associations with radiation treatment morbidity [6, 29–32]. Although the mechanisms for these findings are unclear, previous studies noted patients suffering from financial burden may use cost-coping strategies such as decreasing spending on basic needs and delaying or avoiding medical care [21, 33–35]. When levying such scenarios in the setting of primary head and neck cancer RT, reduced spending may make adherence to specific dietary recommendations difficult and avoiding prompt medical care could lead to more admissions or emergent presentations for neglected symptoms. While speculative, this is supported by our observation of patients reporting more severe RT induced toxicities (fatigue, nausea, vomiting, swallowing), and could relate to worse survival outcomes as well. Future work is required to determine how baseline financial toxicity influences treatment morbidity, and to determine if attempts at addressing such burden a priori could improve patient outcomes.

Consistent with prior literature, baseline COST financial toxicity was associated with worse pre- and post-treatment HRQoL outcomes encompassing global health, role, and emotional functional scales [3, 23, 36–39]. Interestingly, financial toxicity as measured by COST has demonstrated similar correlations even in countries offering free or low-cost national healthcare systems [23, 37]. While this could be explained by common aspects of financial toxicity, such as time away from work, loss of productivity, or loss of income, it is unclear how variations in healthcare payor systems may modulate the relationship between financial well-being and patient-reported outcomes. Within the United States, out-of-pocket expenses are highly variable for insured patients, and further financial support efforts seem to do little to mitigate financial toxicity [40]. In a study of 254 cancer patients, 75% applied for medication copayment assistance programs, with 42% going on to report catastrophic financial burden and 20–25% taking less or stopping prescribed medications altogether [40]. Such cost reduction strategies provide a clear example of compromised care for those suffering from financial toxicity, and emphasizes a need to institute additional financial services, cost-conscious approaches, or policies to provide assistance for these patients. Such efforts can include programs tailored to reintegrate patients into the workforce after treatment, multidisciplinary clinics to consolidate patient visits, travel assistance or reimbursement programs, in addition to assistance with copayments or other insurance premiums [41–45]. Financial navigation is an intervention that has demonstrated particular promise, focusing on counseling to assist with medical and non-medical expenses, and could mitigate financial toxicity among patients with a COST score less than 14.5 [46]. Future work should investigate how such measures may improve financial toxicity and associated HRQoL and cancer-specific outcomes for these patients.

The identification of financial toxicity as an independent factor for worse survival after potentially curative treatment is of significant concern. While limited by a small sample size and acknowledged cohort heterogeneity, the present study suggests grade 1 or higher financial toxicity leads to an increased 20% absolute risk for death. While it is unclear if financial assistance designed to alleviate financial toxicity would effectively reduce this risk of death, it raises the question of whether treatment and associated costs may be inflicting harms that mitigate benefit for these patients. These findings likely quantify, to a degree, ingrained inequities in healthcare for historically disadvantaged populations, and raises some urgency for practitioners and institutions to make conscientious efforts to address such patient diversity. In light of the complex mechanisms leading to inequity for some socioeconomic group, our findings support screening for financial toxicity among patients seeking care at our facility. How such screening should be addressed remains controversial, however, as current approaches are heterogeneous and there is currently no standard for doing so in the United States [47, 48]. Future work should aim to standardize financial toxicity assessments in cancer patients, and determine potential benefit of longitudinal screening approaches within oncology and non-oncology encounters [49].

While this study is the first to assess COST financial toxicity in a head and neck RT cohort, there are inherent limitations related to patient selection and questionnaire administration that prevent wider generalization of our findings. This includes limitations related to the single-institution design such as limited sample size, selection bias, response bias, and measurement bias. Examples of this include administration of the PRO measures in an insured patient population and included a narrow scope of head and neck malignancies consisting mostly of advanced stage cutaneous or oral cavity disease. Further, given the small sample size and complexity of associated socioeconomic risk factors, multivariate assessments were purposely limited given potential for overfitting. Lastly, surveys were also administered solely in English, and while reading comprehension was screened for in the assessed cohort, patients who report a preference for non-English languages may have less reliable reported PRO measures. Given this, generalizability of these findings to other common head and neck cancers and/or the uninsured population is not advised, as financial toxicity implications could vary due to treatment morbidity or other socioeconomic driving factors, respectively.

## Conclusions

In conclusion, financial toxicity both before and after radiation therapy for head and neck cancers was associated with both worse overall survival, treatment morbidity, and patient-reported HRQoL outcomes. This included younger, single, unemployed, black patients with increased risk of hospitalization/emergency room visits and PEG tube placement. These findings highlight potential challenges faced by historically disadvantaged socioeconomic populations, and identifies a potentially modifiable risk factor for their cancer-related outcomes.

## Electronic supplementary material

Below is the link to the electronic supplementary material.

Supplementary material 1

Supplementary material 2

Supplementary material 3

Supplementary material 4

## Data Availability

Data generated and/or analyzed during the current study are not publicly available to protect patient confidentiality.

## References

[1] National Cancer institute. Cancer stat facts: laryngeal Cancer. Surveillance, epidemiology, and end results program. n.d. https://seer.cancer.gov/statfacts/html/laryn.html. accessed September 7, 2024.

[2] National Cancer Institute. Cancer stat facts: oral cavity and Pharynx. Surveillance, epidemiology, and end results program. n.d. https://seer.cancer.gov/statfacts/html/oralcav.html. accessed September 7, 2024.

[3] Harris JP, Ku E, Harada G, Hsu S, Chiao E, Rao P, et al. Severity of financial toxicity for patients receiving palliative radiation therapy. Am J Hosp Palliat Care. 2024;41:592–600. 10.1177/10499091231187999. 37406195 10.1177/10499091231187999 PMC10772523

[4] Longo CJ. Linking intermediate to final “real-world” outcomes: is financial toxicity a reliable predictor of poorer outcomes in cancer? Curr Oncol. 2022;29:2483–89. 10.3390/curroncol29040202. 35448176 10.3390/curroncol29040202 PMC9027087

[5] Mourad M, Jetmore T, Jategaonkar AA, Moubayed S, Moshier E, Urken ML. Epidemiological trends of head and neck cancer in the United States: a seer population study. J Oral Maxillofac Surg. 2017;75:2562–72. 10.1016/j.joms.2017.05.008. 28618252 10.1016/j.joms.2017.05.008 PMC6053274

[6] Ma SJ, Iovoli AJ, Attwood K, Wooten KE, Arshad H, Gupta V, et al. Association of significant financial burden with survival for head and neck cancer patients treated with radiation therapy. Oral Oncol. 2021;115:105196. 10.1016/j.oraloncology.2021.105196. 33578203 10.1016/j.oraloncology.2021.105196 PMC10353569

[7] Mariotto AB, Enewold L, Zhao J, Zeruto CA, Yabroff KR. Medical care costs associated with cancer survivorship in the United States. Cancer Epidemiol Biomarker Prev. 2020;29:1304–12. 10.1158/1055-9965.EPI-19-1534. 10.1158/1055-9965.EPI-19-1534 PMC9514601 32522832

[8] Chen S, Cao Z, Prettner K, Kuhn M, Yang J, Jiao L, et al. Estimates and projections of the global economic cost of 29 cancers in 204 countries and territories from 2020 to 2050. JAMA Oncol. 2023;9:465–72. 10.1001/jamaoncol.2022.7826. 36821107 10.1001/jamaoncol.2022.7826 PMC9951101

[9] Panzone J, Welch C, Morgans A, Bhanvadia SK, Mossanen M, Shenhav-Goldberg R, et al. Association of race with cancer-related financial toxicity. JCO Oncol Pract. 2022;18:e271–83. 10.1200/OP.21.00440. 34752150 10.1200/OP.21.00440

[10] de Souza Ja, Yap BJ, Wroblewski K, Blinder V, Araújo FS, Hlubocky FJ, et al. Measuring financial toxicity as a clinically relevant patient-reported outcome: the validation of the COmprehensive score for financial toxicity (COST). Cancer. 2017;123:476–84. 10.1002/cncr.30369. 27716900 10.1002/cncr.30369 PMC5298039

[11] Peipert JD, Beaumont JL, Bode R, Cella D, Garcia SF, Hahn EA. Development and validation of the functional assessment of chronic illness therapy treatment satisfaction (facit ts) measures. Qual Life Res. 2014;23:815–24. 10.1007/s11136-013-0520-8. 24062239 10.1007/s11136-013-0520-8

[12] Cocks K, Wells JR, Johnson C, Schmidt H, Koller M, Oerlemans S, et al. Content validity of the EORTC quality of life questionnaire QLQ-C30 for use in cancer. Eur J Cancer. 2023;178:128–38. 10.1016/j.ejca.2022.10.026. 36436330 10.1016/j.ejca.2022.10.026

[13] Dueck AC, Mendoza TR, Mitchell SA, Reeve BB, Castro KM, Rogak LJ, et al. Validity and reliability of the us National Cancer Institute’s patient-reported outcomes version of the common terminology criteria for adverse events (PRO-CTCAE). JAMA Oncol. 2015;1:1051–59. 10.1001/jamaoncol.2015.2639. 26270597 10.1001/jamaoncol.2015.2639 PMC4857599

[14] Census Bureau Data. n.d. https://data.census.gov/. accessed May 7, 2024.

[15] FACIT-TS-G. FACIT Group. n.d. https://www.facit.org/measures/facit-ts-g. accessed September 7, 2024.

[16] FACIT-COST. FACIT Group. n.d. https://www.facit.org/measures/facit-cost. accessed September 7, 2024.

[17] Quality of life group. EORTC QLQ-C30. EORTC – quality of life 2017. https://qol.eortc.org/questionnaires/core/eortc-qlq-c30/. accessed September 7, 2024.

[18] Patient-reported outcomes version of the common terminology criteria for adverse events (PRO-CTCAE). n.d. https://healthcaredelivery.cancer.gov/pro-ctcae/. accessed September 7, 2024. 10.1186/s41687-023-00598-4 PMC10260717 37306774

[19] De Souza JA, Wroblewski K, Proussaloglou E, Nicholson L, Hantel A, Wang Y. Validation of a financial toxicity (ft) grading system. JCO. 2017;35:6615–6615. 10.1200/.JCO.2017.35.15_suppl.6615.

[20] De Souza JA, Aschebrook-Kilfoy B, Grogan R, Yap BJ, Daugherty C, Cella D. Grading financial toxicity based upon its impact on health-related quality of life (HRQol). JCO. 2016;34:16–16. 10.1200/jco.2016.34.3_suppl.16.

[21] Esselen KM, Baig RA, Gompers A, Stack-Dunnbier H, Hacker MR, Jang JW. Factors associated with increased financial toxicity after the completion of radiation treatment for gynecologic cancer. Support Care Cancer. 2023;31:388. 10.1007/s00520-023-07849-6. 37300721 10.1007/s00520-023-07849-6 PMC10256970

[22] de Souza Ja, Yap BJ, Hlubocky FJ, Wroblewski K, Ratain MJ, Cella D, et al. The development of a financial toxicity patient-reported outcome in cancer: the COST measure. Cancer. 2014;120:3245–53. 10.1002/cncr.28814. 24954526 10.1002/cncr.28814

[23] Shim S, Kang D, Kim N, Han G, Lim J, Kim H, et al. Validation of Korean version of the COmprehensive score for financial toxicity (COST) among breast cancer survivors. Cancer Res Treat. 2021;54:834–41. 10.4143/crt.2021.784. 34645130 10.4143/crt.2021.784 PMC9296937

[24] Yusuf M, Pan J, Rai SN, Eldredge-Hindy H. Financial toxicity in women with breast cancer receiving radiation therapy: final results of a prospective observational study. Pract Radiat Oncol. 2022;12:e79–89. 10.1016/j.prro.2021.11.003. 34896597 10.1016/j.prro.2021.11.003

[25] Raggini E, Mattavelli D, Zigliani G, Bossi P, Piazza C. Measuring financial toxicity in head and neck cancer: a systematic review. Acta Otorhinolaryngol Ital. 2024;44:1–12. 10.14639/0392-100X-N2762. 38420716 10.14639/0392-100X-N2762 PMC10914354

[26] Yabroff KR, Dowling EC, Guy GP, Banegas MP, Davidoff A, Han X, et al. Financial hardship associated with cancer in the United States: findings from a population-based sample of adult cancer survivors. J Clin Oncol. 2016;34:259–67. 10.1200/JCO.2015.62.0468. 26644532 10.1200/JCO.2015.62.0468 PMC4872019

[27] Ramsey S, Blough D, Kirchhoff A, Kreizenbeck K, Fedorenko C, Snell K, et al. Washington State cancer patients found to be at greater risk for bankruptcy than people without a cancer diagnosis. Health Aff (millwood). 2013;32:1143–52. 10.1377/hlthaff.2012.1263. 23676531 10.1377/hlthaff.2012.1263 PMC4240626

[28] Meeker CR, Wong Y-N, Egleston BL, Hall MJ, Plimack ER, Martin LP, et al. Distress and financial distress in adults with cancer: an age-based analysis. J Natl Compr Canc Netw. 2017;15:1224–33. 10.6004/jnccn.2017.0161. 28982748 10.6004/jnccn.2017.0161 PMC7569506

[29] Kircher S, Duan F, An N, Gareen IF, Sicks JD, Sadigh G, et al. Patient-reported financial burden of treatment for colon or rectal cancer. JAMA Netw Open Open. 2024;7:e2350844. 10.1001/jamanetworkopen.2023.50844. 10.1001/jamanetworkopen.2023.50844 PMC10777253 38194233

[30] Perrone F, Jommi C, Di Maio M, Gimigliano A, Gridelli C, Pignata S, et al. The association of financial difficulties with clinical outcomes in cancer patients: secondary analysis of 16 academic prospective clinical trials conducted in Italy. Ann Oncol. 2016;27:2224–29. 10.1093/annonc/mdw433. 27789469 10.1093/annonc/mdw433

[31] Klein J, Bodner W, Garg M, Kalnicki S, Ohri N. Pretreatment financial toxicity predicts progression-free survival following concurrent chemoradiotherapy for locally advanced non-small-cell lung cancer. Future Oncol. 2019;15:1697–705. 10.2217/fon-2018-0874. 30977688 10.2217/fon-2018-0874

[32] Ramsey SD, Bansal A, Fedorenko CR, Blough DK, Overstreet KA, Shankaran V, et al. Financial insolvency as a risk factor for early mortality among patients with cancer. J Clin Oncol. 2016;34:980–86. 10.1200/JCO.2015.64.6620. 26811521 10.1200/JCO.2015.64.6620 PMC4933128

[33] Esselen KM, Stack-Dunnbier H, Gompers A, Hacker MR. Crowdsourcing to measure financial toxicity in gynecologic oncology. Gynecologic Oncol. 2021;161:595–600. 10.1016/j.ygyno.2021.01.040. 10.1016/j.ygyno.2021.01.040 PMC10029746 33551197

[34] Bouberhan S, Shea M, Kennedy A, Erlinger A, Stack-Dunnbier H, Buss MK, et al. Financial toxicity in gynecologic oncology. Gynecologic Oncol. 2019;154:8–12. 10.1016/j.ygyno.2019.04.003. 10.1016/j.ygyno.2019.04.003 PMC7001853 31053404

[35] Sadigh G, Switchenko J, Weaver KE, Elchoufi D, Meisel J, Bilen MA, et al. Correlates of financial toxicity in adult cancer patients and their informal caregivers. Support Care Cancer. 2022;30:217–25. 10.1007/s00520-021-06424-1. 34255179 10.1007/s00520-021-06424-1 PMC8639637

[36] de Souza Ja, Yap BJ, Wroblewski K, Blinder V, Araújo FS, Hlubocky FJ, et al. Measuring financial toxicity as a clinically relevant patient-reported outcome: the validation of the Comprehensive score for financial toxicity (COST). Cancer. 2017;123:476–84. 10.1002/cncr.30369. 27716900 10.1002/cncr.30369 PMC5298039

[37] Ripamonti CI, Chiesi F, Di Pede P, Guglielmo M, Toffolatti L, Gangeri L, et al. The validation of the Italian version of the comprehensive score for financial toxicity (COST). Support Care Cancer. 2020;28:4477–85. 10.1007/s00520-019-05286-y. 31925533 10.1007/s00520-019-05286-y

[38] Thom B, Mamoor M, Lavery JA, Baxi SS, Khan N, Rogak LJ, et al. The experience of financial toxicity among advanced melanoma patients treated with immunotherapy. J Psychosoc Oncol. 2021;39:285–93. 10.1080/07347332.2020.1836547. 33103948 10.1080/07347332.2020.1836547 PMC8437327

[39] Pangestu S, Rencz F. Comprehensive score for financial toxicity and health-related quality of life in patients with cancer and survivors: a systematic review and meta-analysis. Value Health. 2023;26:300–16. 10.1016/j.jval.2022.07.017. 36064514 10.1016/j.jval.2022.07.017

[40] Zafar SY, Peppercorn JM, Schrag D, Taylor DH, Goetzinger AM, Zhong X, et al. The financial toxicity of cancer treatment: a pilot study assessing out-of-pocket expenses and the insured cancer Patient’s experience. Oncologist. 2013;18:381–90. 10.1634/theoncologist.2012-0279. 23442307 10.1634/theoncologist.2012-0279 PMC3639525

[41] Lamore K, Dubois T, Rothe U, Leonardi M, Girard I, Manuwald U, et al. Return to work interventions for cancer survivors: a systematic review and a methodological critique. Int J Environ Res Public Health. 2019;16:1343. 10.3390/ijerph16081343. 31014004 10.3390/ijerph16081343 PMC6518012

[42] Parekh KD, Wong WB, Zullig LL. Impact of co-pay assistance on patient, clinical, and economic outcomes. Am J Manag Care. 2022;28:e189–97. 10.37765/ajmc.2022.89151. 35546593 10.37765/ajmc.2022.89151

[43] Voong KR, Liang OS, Dugan P, Torto D, Padula WV, Senter JP, et al. Thoracic oncology multidisciplinary clinic reduces unnecessary health care expenditure used in the workup of patients with non-small-cell lung cancer. Clin Lung Cancer. 2019;20:e430–41. 10.1016/j.cllc.2019.02.010. 30956040 10.1016/j.cllc.2019.02.010

[44] Adams A, Kluender R, Mahoney N, Wang J, Wong F, Yin W. The Impact of financial assistance programs on health care utilization: evidence from Kaiser Permanente. Am Econ Rev Insights. 2022;4:389–407. 10.1257/aeri.20210515. 36338144 10.1257/aeri.20210515 PMC9634821

[45] Khan G, Karabon P, Lerchenfeldt S. Use of prescription assistance programs after the affordable health care act. J Manag Care Spec Pharm. 2018;24. 10.18553/jmcp.2018.24.3.247. 10.18553/jmcp.2018.24.3.247. 10.18553/jmcp.2018.24.3.247 PMC10397826 29485949

[46] Rashidi A, Jung J, Kao R, Nguyen EL, Le T, Ton B, et al. Interventions to mitigate cancer-related medical financial hardship: a systematic review and meta-analysis. Cancer. 2024;130:3198–209. 10.1002/cncr.35367. 38758809 10.1002/cncr.35367 PMC11347103

[47] Samaha NL, Mady LJ, Armache M, Hearn M, Stemme R, Jagsi R, et al. Screening for financial toxicity among patients with cancer: a systematic review. J Am Coll Radiol. 2024;21:1380–97. 10.1016/j.jacr.2024.04.024. 38762031 10.1016/j.jacr.2024.04.024

[48] Khera N, Sugalski J, Krause D, Butterfield R, Zhang N, Stewart FM, et al. Current practices for screening and management of financial distress at NCCN member institutions. J Natl Compr Canc Netw. 2020;18:825–31. 10.6004/jnccn.2020.7538. 32634772 10.6004/jnccn.2020.7538

[49] Bansal R, Anderson D, Cuyegkeng A, Tran T-N, Aijaz A, Dhillon J, et al. Feasibility of screening for financial hardship and health-related social needs at radiology encounters. J Am Coll Radiol. 2024;21:1362–70. 10.1016/j.jacr.2023.12.025. 38159833 10.1016/j.jacr.2023.12.025

