# Supplementary material for: Financial toxicity on treatment outcomes in head & neck cancer patients undergoing radiation therapy
Source: Radiat Oncol. 2025 Nov 25;20:176. doi: 10.1186/s13014-025-02749-x (PMC12648789; doi:10.1186/s13014-025-02749-x)
Supplement: Supplementary file 1 — Supplementary material 1 [file 13014_2025_2749_MOESM1_ESM.pdf]

COST – FACIT (Version 2)

Below is a list of statements that other people with your illness have said are important. **Please circle or mark one number per line to indicate your response as it applies to the <u>past 7 days</u>.**

		Not at all	A little bit	Somewhat	Quite a bit	Very much
FT1	I know that I have enough money in savings, retirement, or assets to cover the costs of my treatment	0	1	2	3	4
FT2	My out-of-pocket medical expenses are more than I thought they would be	0	1	2	3	4
FT3	I worry about the financial problems I will have in the future as a result of my illness or treatment	0	1	2	3	4
FT4	I feel I have no choice about the amount of money I spend on care	0	1	2	3	4
FT5	I am frustrated that I cannot work or contribute as much as I usually do	0	1	2	3	4
FT6	I am satisfied with my current financial situation	0	1	2	3	4
FT7	I am able to meet my monthly expenses	0	1	2	3	4
FT8	I feel financially stressed	0	1	2	3	4
FT9	I am concerned about keeping my job and income, including paid work at home	0	1	2	3	4
FT10	My cancer or treatment has reduced my satisfaction with my present financial situation	0	1	2	3	4
FT11	I feel in control of my financial situation	0	1	2	3	4
FT12	My illness has been a financial hardship to my family and me	0	1	2	3	4

English (Universal)
Copyright 2014, FACIT and The University of Chicago

COST – FACIT (Version 2)

COmprehensive Score for financial Toxicity (COST) Scoring Guidelines (Version 2)

Instructions:*
1. Record answers in "item response" column. If missing, mark with an X
2. Perform reversals as indicated, and sum individual items to obtain a score.
3. Multiply the sum of the item scores by the number of items in the scale, then divide by the number of items answered. This produces the scale score.
4. **The higher the score, the better the Financial Well-Being.**

Subscale	Item Code	Reverse item?		Item response	Item Score
**FINANCIAL**	FT1	0	+	________	=________
**TOXICITY**	FT2	4	-	________	=________
**SCALE**	FT3	4	-	________	=________
	FT4	4	-	________	=________
Score range: 0-44	FT5	4	-	________	=________
	FT6	0	+	________	=________
	FT7	0	+	________	=________
	FT8	4	-	________	=________
	FT9	4	-	________	=________
	FT10	4	-	________	=________
	FT11	0	+	________	=________
	FT12	**Not scored (summary item)**			

Sum individual item scores: ________
Multiply by 11: ________
Divide by number of items answered: ________= Financial Toxicity Score

Copyright 2003 by David Cella, Ph.D.

FACIT-TS-G (Version 4)

Please evaluate your experience on this treatment: ______________________________________
(*name of treatment*)

If you have not completed your treatment, please answer the questions the best you can. All of your answers will be kept confidential.

Please mark one answer for each of the following questions.

		A lot worse	A little worse	About the same	A little better	A lot better
TS1	Compared to what you expected, how do you rate the <u>effectiveness of the treatment</u> so far?	0	1	2	3	4
TS2	Compared to what you expected, how do you rate the <u>side effects of treatment</u> so far?	0	1	2	3	4

		No, not at all	Yes, to some extent	Yes, for the most part	Yes, completely
TS3	Did your doctor(s) help you evaluate the effects of your treatment so far?	0	1	2	3
TS4	Do you feel you received the treatment that was right for you?	0	1	2	3
TS5	Are you satisfied with the effects of this treatment so far?	0	1	2	3

		No	Maybe	Yes
TS6	Would you recommend this treatment to others with your illness?	0	1	2
TS7	Would you choose this treatment again?	0	1	2

		Poor	Fair	Good	Very Good	Excellent
TS8	How do you rate this treatment overall?	0	1	2	3	4

Thank you! Do you have any comments? ______________________________________

____

Copyright 2003 by David Cella, Ph.D.

*For guidelines on handling missing data and scoring options, please refer to the Administration and Scoring Guidelines in the manual or on-line at www.facit.org.

EORTC QLQ-C30 (version 3)

We are interested in some things about you and your health. Please answer all of the questions yourself by circling the number that best applies to you. There are no "right" or "wrong" answers. The information that you provide will remain strictly confidential.

Today's date (Day, Month, Year): 31 □□□□□□□□

	Not at All	A Little	Quite a Bit	Very Much
1. Do you have any trouble doing strenuous activities, like carrying a heavy shopping bag or a suitcase?	1	2	3	4
2. Do you have any trouble taking a long walk?	1	2	3	4
3. Do you have any trouble taking a short walk outside of the house?	1	2	3	4
4. Do you need to stay in bed or a chair during the day?	1	2	3	4
5. Do you need help with eating, dressing, washing yourself or using the toilet?	1	2	3	4

**During the past week:**	Not at All	A Little	Quite a Bit	Very Much
6. Were you limited in doing either your work or other daily activities?	1	2	3	4
7. Were you limited in pursuing your hobbies or other leisure time activities?	1	2	3	4
8. Were you short of breath?	1	2	3	4
9. Have you had pain?	1	2	3	4
10. Did you need to rest?	1	2	3	4
11. Have you had trouble sleeping?	1	2	3	4
12. Have you felt weak?	1	2	3	4
13. Have you lacked appetite?	1	2	3	4
14. Have you felt nauseated?	1	2	3	4
15. Have you vomited?	1	2	3	4
16. Have you been constipated?	1	2	3	4

Please go on to the next page

During the past week:	Not at All	A Little	Quite a Bit	Very Much
17. Have you had diarrhea?	1	2	3	4
18. Were you tired?	1	2	3	4
19. Did pain interfere with your daily activities?	1	2	3	4
20. Have you had difficulty in concentrating on things, like reading a newspaper or watching television?	1	2	3	4
21. Did you feel tense?	1	2	3	4
22. Did you worry?	1	2	3	4
23. Did you feel irritable?	1	2	3	4
24. Did you feel depressed?	1	2	3	4
25. Have you had difficulty remembering things?	1	2	3	4
26. Has your physical condition or medical treatment interfered with your family life?	1	2	3	4
27. Has your physical condition or medical treatment interfered with your social activities?	1	2	3	4
28. Has your physical condition or medical treatment caused you financial difficulties?	1	2	3	4

For the following questions please circle the number between 1 and 7 that best applies to you

29. How would you rate your overall health during the past week?

1	2	3	4	5	6	7
Very poor						Excellent

30. How would you rate your overall quality of life during the past week?

1	2	3	4	5	6	7
Very poor						Excellent

© Copyright 1995 EORTC Quality of Life Group. All rights reserved. Version 3.0
