# Supplementary material for: Financial toxicity on treatment outcomes in head & neck cancer patients undergoing radiation therapy
Source: Radiat Oncol. 2025 Nov 25;20:176. doi: 10.1186/s13014-025-02749-x (PMC12648789; doi:10.1186/s13014-025-02749-x)
Supplement: Supplementary file 2 — Supplementary material 2 [file 13014_2025_2749_MOESM2_ESM.pdf]

All rights reserved. No part of this manual covered by copyrights hereon may be reproduced or transmitted in any form or by any means without prior permission of the copyright holder.

The EORTC QLQ-C30 (in all versions), and the modules which supplement it, are copyrighted and may not be used without prior written consent of the EORTC Data Center.

Requests for permission to use the EORTC QLQ-C30 and the modules, or to reproduce or quote materials contained in this manual, should be addressed to:

QL Coordinator
Quality of Life Unit,
EORTC Data Center,
Avenue E Mounier 83 - B11,
1200 Brussels,
BELGIUM

Tel: +32 2 774 1611
Fax: +32 2 779 4568
Email: abo@eortc.be

Copyright © 1995, 1999, 2001 EORTC, Brussels.
D/2001/6136/001
ISBN 2-9300 64-22-6

Third edition, 2001

EORTC QLQ-C30 Scoring Manual

The EORTC QLQ-C30

Introduction

The EORTC quality of life questionnaire (QLQ) is an integrated system for assessing the health-related quality of life (QoL) of cancer patients participating in international clinical trials. The core questionnaire, the QLQ-C30, is the product of more than a decade of collaborative research. Following its general release in 1993, the QLQ-C30 has been used in a wide range of cancer clinical trials, by a large number of research groups; it has additionally been used in various other, non-trial studies.

This manual contains scoring procedures for the QLQ-C30 versions 1.0, (+3), 2.0 and 3.0; it also contains summary information about supplementary modules.

All publications relating to the QLQ should use the scoring procedures described in this manual.

This manual will be updated at regular intervals, to reflect future changes to the QLQ and to incorporate new supplementary modules.

Background

The EORTC

The European Organisation for Research and Treatment of Cancer (EORTC) was founded in 1962, as an international non-profit organisation. The aims of the EORTC are to conduct, develop, co-ordinate and stimulate cancer research in Europe by multidisciplinary groups of oncologists and basic scientists. Research is accomplished mainly through the execution of large, prospective, randomised, multicentre, cancer clinical trials.

The EORTC Central Office Data Center, created in 1974, is concerned with all aspects of phase II and phase III cancer clinical trials, from their design to the publication of the final results. Since its inception, over 80,000 patients have been entered in trials handled by the EORTC Data Center.

In 1980, the EORTC created the Quality of Life Group, which in 1986 initiated a research programme to develop an integrated, modular approach for evaluating the QoL of patients participating in cancer clinical trials. This led to the development of the EORTC QLQ-C30, a quality of life instrument for cancer patients. To date, more than 2200 studies using the QLQ-C30 have been registered.

EORTC QLQ-C36

A first generation core questionnaire, the EORTC QLQ-C36, was developed in 1987. This 36-item questionnaire was designed to be (1) cancer specific, (2) multidimensional in structure, (3) appropriate for self-administration (i.e. brief and easy to complete), and (4) applicable across a range of cultural settings. Detailed results of the international field-testing of the EORTC QLQ-C36 have been reported (Aaronson *et al.*, 1991). While the overall psychometric results were promising, they also pointed to some areas in which the questionnaire could benefit from further development. Most of the revision involved only minor changes in the wording of items. A few items were found to be non-informative, and were discarded. The only scale requiring substantial revision, because of inadequate reliability, was the eight-item emotional functioning scale. In the next generation of the instrument, this scale was substituted by a four-item emotional functioning scale that had been used previously in EORTC clinical trials.

EORTC QLQ-C30 version 1.0

A second generation core questionnaire, the first version of the 30-item EORTC QLQ-C30 (Appendix 1a), was subsequently developed. The content areas covered by the questionnaire reflect the multi-dimensionality of the QoL construct. This questionnaire was field tested in a cross-cultural sample of lung cancer patients in 13 countries to confirm the hypothesised scale structure, to establish reliability and to evaluate validity (Aaronson *et al.*, 1993).

The QLQ-C30 version 1.0 (QLQ-C30$_{(V1)}$) incorporates five functional scales (physical, role, cognitive, emotional, and social), three symptom scales (fatigue, pain, and nausea and vomiting), a global health status / QoL scale, and a number of single items assessing additional symptoms commonly reported by cancer patients (dyspnoea, loss of appetite, insomnia, constipation and diarrhoea) and perceived financial impact of the disease.

EORTC QLQ-C30 (+3)

The third generation core questionnaire, the 33-item EORTC QLQ-$C30_{(+3)}$ (Appendix 1b), arose following international testing of the QLQ-$C30_{(V1)}$, when refinement of the questionnaire by adding three new test items was recommended. Two of these test items (QLQ-$C30_{(+3)}$/Q_{26}, and QLQ-$C30_{(+3)}$/Q_{27}) were introduced as possible alternatives to the two-item role functioning scale (QLQ-$C30_{(V1)}$/Q_6, QLQ-$C30_{(V1)}$/Q_7), which was found to have sub-optimal internal consistency in previous studies. The third new test item, overall health (QLQ-$C30_{(+3)}$/Q_{32}), was evaluated as a possible replacement for the overall physical condition item (QLQ-$C30_{(V1)}$/Q_{29}) in the global health status / QoL scale, and employed the same 7-point response scale as the other two questions in that scale.

EORTC QLQ-C30 version 2.0

The QLQ-$C30_{(+3)}$ was an interim version, which retained all the original questions of the QLQ-C30 version 1.0 while evaluating the additional three items. There was a marked improvement in the internal consistency of the new role functioning scale. The new overall health item places less emphasis upon physical functioning, and did not alter the internal consistency. Having formally validated these new items, the older questions were replaced by the new ones (Osoba *et al*., 1997). The result was the 30-item version 2.0 of the QLQ, the QLQ-$C30_{(V2)}$ (Appendix 1c).

EORTC QLQ-C30 version 3.0

Version 3.0 of the QLQ-C30 differs from version 2.0 in that it has four-point scales for the first five items (QLQ-$C30_{(V3)}$, Appendix 1d). These are coded with the same response categories as items 6 to 28, namely “Not at all”, “A little”, “Quite a bit” and “Very much.” To allow for these categories, question 4 has been re-worded as “Do you have to stay in a bed or a chair during the day?” Version 3.0 has been tested in EORTC field studies (Bjordal *et al*., 2000).

Version 3.0 is currently the standard version of the QLQ-C30, and should be used for all new studies unless investigators wish to maintain compatibility with previous studies, which used an earlier version of the QLQ-C30.

Latest information about development of the QLQ-C30 and its modules may be found on the EORTC Quality of Life web pages, at:

http://www.eortc.be/home/qol/

Citation and Availability

Citation in published reports

Any publications which describe the use of the EORTC QLQ-C30 or its modules, or which describe analyses of data arising from application of these questionnaires, should explicitly cite the following reference:

> Aaronson NK, Ahmedzai S, Bergman B, Bullinger M, Cull A, Duez NJ, Filiberti A, Flechtner H, Fleishman SB, de Haes JCJM, Kaasa S, Klee MC, Osoba D, Razavi D, Rofe PB, Schraub S, Sneeuw KCA, Sullivan M, Takeda F.
> The European Organisation for Research and Treatment of Cancer QLQ-C30: A quality-of-life instrument for use in international clinical trials in oncology.
> *Journal of the National Cancer Institute* 1993; **85:** 365-376.

For details of the scoring procedure, a suggested format of citation for this manual is:

> Fayers PM, Aaronson NK, Bjordal K, Groenvold M, Curran D, Bottomley A, on behalf of the EORTC Quality of Life Group.
> *The EORTC QLQ-C30 Scoring Manual (3rd Edition).*
> Published by: European Organisation for Research and Treatment of Cancer, Brussels 2001.

Contact address

For information about terms and conditions for using the questionnaire, please contact the Quality of Life Unit, EORTC Data Center.

QL Coordinator,
Quality of Life Unit,
EORTC Data Center,
Avenue E Mounier 83 - B11,
1200 Brussels,
BELGIUM

Tel: +32 2 774 1611
Fax: +32 2 779 4568
Email: abo@eortc.be

Scoring procedures

General principles of scoring

The QLQ-C30 is composed of both multi-item scales and single-item measures. These include five functional scales, three symptom scales, a global health status / QoL scale, and six single items. Each of the multi-item scales includes a different set of items - no item occurs in more than one scale.

All of the scales and single-item measures range in score from 0 to 100. A high scale score represents a higher response level.
Thus a **high score for a functional scale** represents a *high / healthy level of functioning,*
a **high score for the global health status / QoL** represents a *high QoL,*
but a **high score for a symptom scale / item** represents a *high level of symptomatology / problems.*

The principle for scoring these scales is the same in all cases:

1. Estimate the average of the items that contribute to the scale; this is the *raw score*.

2. Use a linear transformation to standardise the raw score, so that scores range from 0 to 100; a higher score represents a higher ("better") level of functioning, or a higher ("worse") level of symptoms.

Coding of the scoring procedure is presented in Appendix 3 for three major statistical packages.

Technical Summary

In practical terms, if items $I_1, I_2, \ldots I_n$ are included in a scale, the procedure is as follows:

Raw score
Calculate the raw score
$RawScore = RS = (I_1 + I_2 + \ldots + I_n)/n$

Linear transformation
Apply the linear transformation to 0-100 to obtain the score *S*,

Functional scales: $S = \left\{1 - \frac{(RS-1)}{range}\right\} \times 100$

Symptom scales / items: $S = \{(RS-1)/range\} \times 100$

Global health status / QoL: $S = \{(RS-1)/range\} \times 100$

Range is the difference between the maximum possible value of *RS* and the minimum possible value. The QLQ-C30 has been designed so that all items in any scale take the same range of values. Therefore, the range of *RS* equals the range of the item values. Most items are scored 1 to 4, giving *range* = 3. The exceptions are the items contributing to the global health status / QoL, which are 7-point questions with *range* = 6, and the initial yes/no items on the earlier versions of the QLQ-C30 which have *range* = 1.

Scoring the EORTC QLQ-C30 version 3.0

Table 1: Scoring the QLQ-C30 version 3.0

	Scale	Number of items	Item range*	**Version 3.0** Item numbers	Function scales
**Global health status / QoL**					
Global health status/QoL (revised)†	QL2	2	6	29, 30	
**Functional scales**					
Physical functioning (revised)†	PF2	5	3	1 to 5	F
Role functioning (revised)†	RF2	2	3	6, 7	F
Emotional functioning	EF	4	3	21 to 24	F
Cognitive functioning	CF	2	3	20, 25	F
Social functioning	SF	2	3	26, 27	F
**Symptom scales / items**					
Fatigue	FA	3	3	10, 12, 18	
Nausea and vomiting	NV	2	3	14, 15	
Pain	PA	2	3	9, 19	
Dyspnoea	DY	1	3	8	
Insomnia	SL	1	3	11	
Appetite loss	AP	1	3	13	
Constipation	CO	1	3	16	
Diarrhoea	DI	1	3	17	
Financial difficulties	FI	1	3	28	

* *Item range* is the difference between the possible maximum and the minimum response to individual items; most items take values from 1 to 4, giving *range* = 3.

† (revised) scales are those that have been changed since version 1.0, and their short names are indicated in this manual by a suffix "2" – for example, PF2.

For all scales, the *RawScore*, *RS*, is the mean of the component items:

$$RawScore = RS = (I_1 + I_2 + ... + I_n)/n$$

Then for **Functional scales**:

$$Score = \left\{1 - \frac{(RS - 1)}{range}\right\} \times 100$$

and for **Symptom scales / items** and **Global health status / QoL:**

$$Score = \{(RS - 1)/range\} \times 100$$

Examples:

Emotional functioning

$$RawScore = (Q_{21} + Q_{22} + Q_{23} + Q_{24})/4$$
$$\text{EF } Score = \{1 - (RawScore - 1)/3\} \times 100$$

Fatigue

$$RawScore = (Q_{10} + Q_{12} + Q_{18})/3$$
$$\text{FA } Score = \{(RawScore - 1)/3\} \times 100$$

Scoring earlier versions of the EORTC QLQ-C30

Table 2: Scoring the QLQ-C30 version 2.0

For the QLQ-C30(V2), the only difference is that Q_1 to Q_5 are coded yes/no, with *range* = 1. The following should be added to Table 1, and the revised scale for PF2 deleted.

	Scale	Number of items	Item range*	**Version 2.0** Item numbers	Function scales
Physical functioning (original scale)	PF	5	1	1 to 5	F
Delete PF2					

* *Item range* is the difference between the possible maximum and the minimum response to individual items; most items take values from 1 to 4, giving *range* = 3.

Table 3: Scoring the QLQ-C30 (+3)

The QLQ-C30(+3) used the earlier scale for PF, and included both the original and revised versions of QL and RF. The items for SF and FI were placed after the new items 26 and 27, and are hence numbered 28 to 30. The following changes should be made to Table 1, and the revised scale for PF2 deleted.

	Scale	Number of items	Item range*	**Version (+3)** Item numbers	Function scales
Global health status/QoL	QL	2	6	31, 33	
Global health status/QoL (revised)	QL2	2	6	32, 33	
Physical functioning	PF	5	1	1 to 5	F
Role functioning	RF	2	1	6, 7	F
Role functioning (revised)	RF2	2	3	26, 27	F
Social functioning	SF	2	3	28, 29	F
Financial difficulties	FI	1	3	30	
Delete PF2					

* *Item range* is the difference between the possible maximum and the minimum response to individual items; most items take values from 1 to 4, giving *range* = 3.

Table 4: Scoring the QLQ-C30 version 1.0

The QLQ-C30(V1) used the original scales for QL, PF and RF, and so QL2, RF2 and PF2 should be deleted and the following changes made to Table 1.

	Scale	Number of items	Item range*	**Version 1.0** Item numbers	Function scales
Global health status/QoL	QL	2	6	29, 30	
Physical functioning	PF	5	1	1 to 5	F
Role functioning	RF	2	1	6, 7	F
Delete QL2, RF2 and PF2					

* *Item range* is the difference between the possible maximum and the minimum response to individual items; most items take values from 1 to 4, giving *range* = 3.

Missing data

Missing data may be classified as either missing items (one or more missing answers to questions within a questionnaire), or missing forms (the whole questionnaire is missing for a patient). Fayers and Machin (2000) describe methods of analysis for use when data are missing, including imputation techniques.

Missing items

Sometimes a patient will fail to answer a few questions on the QLQ-C30. Our experience to date suggests that less than 2% of patient data will be missing for the QLQ core questionnaire. However, supplementary modules addressing, for example, sexuality issues may have more serious problems with patient compliance. In theory it is important to distinguish between items, which are accidentally missing (commonly described as "missing completely at random"), and items, which are missing for a particular reason. For example, if patients feel very poorly with respect to one item they might wish to avoid answering that question. In practice, however, there is likely to be no way of deciding whether there was a specific reason for the missing values and, in general, it would seem likely that most missing items occur completely at random. In such cases the investigator may wish to calculate the scores based upon those items that were completed, possibly by "imputing" or estimating the missing item.

Various statistical methods exist for imputing values. One might, for example, use multivariate techniques that attempt to estimate the most likely value given information about (a) that patient's previous responses to the same item, (b) other patients' responses at a similar stage in their disease progression and therapy, or (c) the inter-relations and covariance structure with other items.

A simple method for imputing items from multi-item scales, which has been used by many QoL instruments, is the following: if at least half of the items from the scale have been answered, assume that the missing items have values equal to the average of those items which *are* present for that respondent. However, this rule is not always appropriate, and caution should be exercised. Application of this method of imputation is simpler than it perhaps seems; it can be shown that this is algebraically equivalent to using all items, which were completed, and applying the equations already given under "Scoring procedures" for calculating the scale scores; the missing items are simply ignored when making the calculations. Hence the above equations for multi-item scales can be used whenever at least half the items are completed.

Example:

Emotional functioning if Q_{23} *is missing*
(3 items not missing)

$$RawScore = (Q_{21} + Q_{22} + Q_{24})/3$$

$$\text{EF } Score = \{1 - (RawScore - 1)/3\} \times 100$$

For example, role functioning (RF) and cognitive functioning (CF) each contain 2 items, and so these scales can be estimated whenever one of their constituent items is present; physical functioning contains 5 items, and so at least 3 need to have been completed. Using this method, none of the single-item measures can be imputed.

Summary – Missing items

- Have at least half of the items from the scale been answered?
- If *Yes,* use all the items that were completed, and apply the standard equations given on the previous pages for calculating the scale scores; ignore any items with missing values when making the calculations.
- If *No,* set scale score to missing.
- For single-item measures, set score to missing.

Missing forms

As experience with QoL research has increased among the cancer treatment community, so has the need for innovative strategies to prevent, identify and deal with the problem of missing data. In addition to the potential for incomplete data that can occur when introducing researchers to a relatively new type of patient outcome, QoL assessments are likely to be missed because of negative events that are experienced by patients, such as treatment toxicities, and patients dropping out due to disease progression or even death. Successful integration of QoL endpoints into clinical trials, therefore, involves a comprehensive approach encompassing issues of research design, study implementation, and statistical analysis methods.

When initiating a clinical trial it is essential that an adequate infrastructure be in place to ensure that the study is managed properly and efficiently. The EORTC Quality of Life Group has developed a manual *"Guidelines for Assessing Quality of Life in EORTC Clinical Trials"* which details the issues which need to be addressed in the design of a clinical trial to ensure that QoL is adequately addressed in the study (Young *et al*., 1999). Education and training of clinical staff regarding the importance of QoL endpoints, combined with centralised quality assurance strategies, are crucial to the successful integration of these endpoints into clinical trials. Even with such efforts, however, there will be incomplete observations. Although the problem of missing forms is not unique to QoL research, it presents an unusual challenge in that the information is provided by patient self-report at a particular point in time, and thus cannot be retrieved at a later date from medical charts, as is often possible with other types of clinical data.

It is useful to document and report the extent of and reasons for missing data. By identifying the reasons for missing questionnaires it may be possible to learn more about the problems of collecting QoL questionnaires, e.g. institution-related factors, and circumvent these problems in future research. The reasons why questionnaires have not been completed may also provide useful information to take into account at the time of analysis. For example, if the main reasons for observing missing questionnaires is administrative failure, then provided the extent of missingness is not too large, the missing forms may not pose too much of a problem. However, if the main reason for missingness is due to patients feeling too ill to fill out the questionnaire, care has to be taken, as there may be a bias in terms of reporting of results. Fayers and Machin (2000) discuss the issue of bias and suggest ways in which it may be possible to reduce the bias by taking covariates into account. Logistic regression models can also be used to evaluate the association between compliance and selected factors.

Troxel *et al.* (1998) described some statistical techniques for assessing and analysing QoL data in the presence of incomplete observations.

Various approaches have been suggested for imputing values, such as mean imputation, regression imputation and last observation carried forward. A major advantage of imputation is that, once the values have been filled in, standard complete data methods of analysis can be used. Some problems do exist using single imputation, e.g. an imputed value is treated as if it were an observed value. This can cause problems, as summary statistics such as percentiles, variances and confidence intervals may have incorrect estimates and hence any inferences that are drawn may be misleading.

Summary – Missing forms

Sufficient care and attention should be taken at the design stage of a study to ensure that an adequate infrastructure, including appropriate personnel and material, is available to carry out the study. No matter how well the analysis is thought out and how accurate the assumptions are about the missing data process, inferences based on incomplete data are not as convincing as inferences based on a complete dataset.

Theory of scaling

The scaling technique described above is based upon the widely applied Likert method of summated scales, in which the constituent items within each scale are simply summed. This makes several assumptions about the nature of the items, the most important of which are (a) that it is appropriate to give equal weight to each item, and (b) that each item is graded on a linear or equal-interval scale. Both these assumptions are questionable, and it might be thought that more sophisticated scaling and scoring procedures would be preferable. Fortunately, however, it has been shown that simple linear scoring systems are surprisingly robust (Dawes, 1979). This has led Cox *et al.* (1992) to propose that "simple integer scoring is likely to be enough for many purposes."

Linearity of items

Methods for assessing the adequacy of linear scores have been reviewed by Cox and Wermuth (1994). At present we have no grounds to believe that the EORTC QLQ items are sufficiently non-linear to warrant any correction before using them in summated scales.

Weighting of items

The use of weights is a far more complex question. Alternative methods for assigning weights include:

- Analysis techniques such as factor analysis or other data-orientated weights (e.g. Gorsuch, 1983; Olschewski and Schumacher, 1990). However, many authors have noted the inherent instability of factor scores, and recommend that the use of factor analysis should be confined to exploring factor structures and testing hypotheses.
- Techniques which attempt to elicit patients' personal utilities or preferences. Drummond *et al.* (1997) describe some of the principal methods that are based upon either the "standard gamble" or the "time trade-off" method. Cox *et al.* (1992) offer a critique of the use of utility methods, and in particular time trade-off, in the context of clinical trials.
- Assignment of arbitrary weights according to opinions of the patients, the investigators, or similar groups (e.g. Simes, 1986).

Cox *et al.* (1992), Olschewski and Schumacher (1990) discuss alternative scaling systems and the problems of choosing, applying and evaluating scaling methods. The inherent difficulties, together with the relative robustness of simple methods, have led many QoL instruments to employ the Likert summated score method.

The EORTC Quality of Life Group is currently exploring alternative scoring procedures, including the use of Rasch models and item response theory (IRT). Structural equation modelling is also being used to investigate higher order factors. At the present time we recommend using scales based upon unweighted summed scores. Also, it should be noted that we caution strongly against the use of a total, global score based upon the sum of all items. The Global health status / QoL scale (based upon Q_{29} and Q_{30} in the QLQ-C30(V3)) should be used as the overall summary measure.

Summary

We currently advocate (unweighted) summated scales. However, work is in progress to explore alternative techniques for evaluating QLQ-C30 scores.

Interpretation of scores

As described in this manual, the raw QLQ-C30 scores can be transformed to scores ranging from 0 to 100. The use of these transformed scores has several advantages, but transformed scores may be difficult to interpret. For example, what does an emotional function score of 60 or a difference of 15 mean? Also, there are no grounds for regarding, say, an emotional function score of 60 as being equally good or bad as scores of 60 on the other functioning scales. However, there are a number of ways to ease the interpretation of QLQ-C30 results.

- One can *report the raw scores* in addition to the transformed scores. For example, it may be clinically relevant to know the proportion of patients that are 'Quite a bit' or 'Very much' constipated. This also applies to results from multi-item scales when the responses to the individual items are of interest. In some cases it may be useful to dichotomise scores, for example by grouping scores into 'Not at all' vs. 'Any extent'.

- The scores can be *compared against published data*, e.g. by using the data for comparable groups of patients published in the *EORTC QLQ-C30 Reference Values* manual (Fayers *et al.*, 1998). In the *Reference Values* manual, data are shown for the main cancer sites, divided by stage of disease. General population data based on large random samples from the general population in Norway and Sweden, and females in Denmark are also published (Hjermstad *et al.*, 1998; Klee *et al.*, 1997; Michelson *et al*., 2000).

Changes in scores over time and *differences between groups* may be more difficult to interpret than absolute scores. The fact that a change is statistically significant does not necessarily imply that it also has clinical significance. Lydick and Epstein (1993) reviewed the different approaches used to define the 'Minimal Clinically Important Difference' and grouped these into *anchor-based* and *distribution-based interpretations*.

Anchor-based interpretations compare the changes seen in QoL scores ('anchored') against other clinical changes or results. Examples of such approaches used with the QLQ-C30 include:

- Osoba *et al.* developed the *Subjective Significance Questionnaire* (SSQ) (Osoba *et al.*, 1998). The SSQ asks patients about *perceived changes* in physical, emotional, and social functioning and in global QL, using a 7-point scale ranging from 'much worse' over 'no change' to 'much better'. Patients filled in the QLQ-C30 at two occasions. At the second completion they also filled in the SSQ. Patients who reported 'a little' change for better or worse on a particular scale (function or symptom) had QLQ-C30 changes about 5 to 10. Those reporting 'moderate' change had changed about 10 to 20, and 'very much' change corresponded to a change greater than 20.

- King (1996) used data from 14 published studies employing the QLQ-C30 or the QLQ-C36, grouping patients according to performance status, weight loss, toxicity, and extent or severity of disease. For each QLQ-C30 scale, the article shows differences in mean scores found between groups differing with respect to the clinical criteria.

Distribution-based interpretations are based on the statistical distributions of results. The most commonly used statistics are Cohen's *effect size* (ES), which relates the observed change to the baseline standard deviation (Fayers and Machin, 2000), or the *standardised response mean* (SRM), which uses the standard deviation of the change. In both Osoba's and King's articles, effect sizes were found to increase in concordance with increasing changes in QLQ-C30 scores and SSQ ratings.

Use of statistical packages

Complete examples of coding for SAS, SPSS and STATA are included in Appendix 3.

Here, however, annotated examples describe the method of coding the transformations and allowance for missing values, using SAS and SPSS.

SAS Commands for scoring the QLQ-C30

SAS statements (SAS for Windows, release 6.08) to calculate the score for the EF scale could be written as follows; XNUM is used to count the number of non-missing items, which should be at least half the total NITEMS items in the scale. Thus this code calculates the average of the non-missing values, and transforms this average to range from 0 to 100 provided that the patient has completed at least half the necessary items.

For emotional functioning (EF) there are 4 items, each with a range of 3:

```
NITEMS              =  4;
XNUM                =  N(OF Q21,Q22,Q23,Q24);
XMEAN               =  MEAN(OF Q21,Q22,Q23,Q24);
IF  XNUM  GE  NITEMS / 2  THEN
        EF          =  (1 - (XMEAN-1)/3 ) * 100;
```

For dyspnoea (DY), a symptom comprising a single item with a range of 3:

```
DY                  =  ((Q8-1)/3) * 100;
```

SPSS Commands for scoring the QLQ-C30

SPSS statements (SPSS for Windows, release 7.5) to calculate the score for the EF scale could be written as follows; XNUM is used to count the number of non-missing items, which should be at least half the total NITEMS items in the scale. Thus this code calculates the average of the non-missing values, and transforms this average to range from 0 to 100 provided that the patient has completed at least half the necessary items.

For emotional functioning (EF) there are 4 items, each with a range of 3:

```
COMPUTE NITEMS   =  4.
COMPUTE XMEAN    =  MEAN (Q21,Q22,Q23,Q24).
COMPUTE XNUM     =  NVALID (Q21,Q22,Q23,Q24).
IF  (XNUM  GE  NITEMS / 2)
        EF       =  (1 - (XMEAN-1)/3 ) * 100.
```

For dyspnoea (DY), a symptom comprising a single item with a range of 3:

```
COMPUTE DY       =  ((Q8-1)/3) * 100.
```

QLQ Supplementary modules

QLQ supplementary modules

An essential component of the EORTC QLQ development strategy involves the use of supplementary questionnaire modules which, when employed in conjunction with the QLQ-C30, can provide more detailed information relevant to evaluating the QoL in specific patient populations. A module may be developed to assess: (1) symptoms related to a specific tumour site (e.g. urinary symptoms in prostate cancer); (2) side effects associated with a given treatment (e.g. chemotherapy-induced neuropathy); or (3) additional QoL domains affected by the disease or treatment (e.g. sexuality, body-image, fear of disease recurrence, etc.).

Modules are developed according to formal guidelines, which have been published as an EORTC Quality of Life Group manual: *Guidelines for Developing Questionnaire Modules*, Sprangers *et al*., 1998a, 1998b. These guidelines outline four *phases* in the module development process:
(1) Generation of relevant QoL issues
(2) Development of the questionnaire items and scales
(3) Module pre-testing
(4) Large scale, international, field studies.

All modules being developed by the EORTC Quality of Life Group are subjected to the rigorous development procedures specified in the guidelines, culminating in large scale international field-testing. The development process for each of the modules is documented in internal reports to the Group, and modules are approved only after formal review of these reports.

All modules are copyrighted in the same way as the core questionnaire. The modules may not be used without prior written consent of the EORTC Data Center or the module developers.

The following modules are currently available for general use, to supplement the core EORTC QLQ-C30. At the time of writing, these are the only modules that have been or are currently being validated in a large-scale international field study.

- **Breast cancer module: QLQ-BR23**
- **Head & neck cancer module: QLQ-H&N35**
- **Lung cancer module: QLQ-LC13**
- **Oesophageal cancer module: QLQ-OES24**
- **Ovarian cancer module: QLQ-OV28**

The contact address for these modules is: QL Coordinator, Quality of Life Unit, EORTC Data Center, Avenue E Mounier 83 - B11, 1200 Brussels, Belgium. Tel: +32 2 774 1611.
Fax: +32 2 779 4568, Email: abo@eortc.be.

Latest information about development of the QLQ supplementary modules may be found on the EORTC Quality of Life web pages, at:
http://www.eortc.be/home/qol/

Breast cancer module: QLQ-BR23

The breast cancer module is meant for use among patients varying in disease stage and treatment modality (i.e. surgery, chemotherapy, radiotherapy and hormonal treatment) (Sprangers *et al.*, 1996). The module comprises 23 questions assessing disease symptoms, side effects of treatment (surgery, chemotherapy, radiotherapy and hormonal treatment), body image, sexual functioning and future perspective (Appendix 2a). The module has been developed according to the guidelines, and approved after formal review. Validation studies in The Netherlands, Spain and the United States have been completed. It has been field tested in a larger cross-cultural study involving 12 countries (EORTC Protocol 15931).

Scoring of the breast cancer module

The breast cancer module incorporates five multi-item scales to assess systemic therapy side effects, arm symptoms, breast symptoms, body image and sexual functioning. In addition, single items assess sexual enjoyment, hair loss and future perspective.

The scoring approach for the QLQ-BR23 is identical in principle to that for the function and symptom scales / single items of the QLQ-C30.[†]

	Scale	Number of items	Item range*	QLQ-BR23 Item numbers	†
**Functional scales**					
Body image	BRBI	4	3	9 – 12	F
Sexual functioning †	BRSEF	2	3	14,15	†
Sexual enjoyment †	BRSEE	1	3	16	†
Future perspective	BRFU	1	3	13	F
**Symptom scales / items**					
Systemic therapy side effects	BRST	7	3	1 – 4,6,7,8	
Breast symptoms	BRBS	4	3	20 – 23	
Arm symptoms	BRAS	3	3	17,18,19	
Upset by hair loss	BRHL	1	3	5	

* "Item range" is the difference between the possible maximum and the minimum response to individual items.

† Items for the scales marked † are scored positively (i.e. "very much" is best) and therefore use the same algebraic equation as for symptom scales; however, the Body Image scale uses the algebraic equation for functioning scales.

BRSEE, sexual enjoyment, is not applicable if item 15 is "not at all."

BRHL, upset by hair loss, is not applicable if item 4 is "not at all."

Head & Neck cancer module: QLQ-H&N35

The head & neck cancer module is meant for use among a wide range of patients with head & neck cancer, varying in disease stage and treatment modality (i.e. surgery, radiotherapy and chemotherapy) (Bjordal and Kaasa, 1992; Bjordal *et al.*, 1994, 1999, 2000). The module comprises 35 questions assessing symptoms and side effects of treatment, social function and body image/sexuality (Appendix 2b). The module has been developed according to the guidelines, and pretested on patients from Norway, Sweden, Denmark, the UK and French-speaking Belgium. It has been field tested in Norway, Sweden and The Netherlands, and in a large cross-cultural study involving more than ten countries (EORTC Protocol 15941).

Scoring of the head & neck cancer module

The head & neck cancer module incorporates seven multi-item scales that assess pain, swallowing, senses (taste and smell), speech, social eating, social contact and sexuality. There are also eleven single items. For all items and scales, high scores indicate more problems (i.e. there are no function scales in which high scores would mean better functioning).

The scoring approach for the QLQ-H&N35 is identical in principle to that for the symptom scales / single items of the QLQ-C30.

Scale name	Scale	Number of items	Item range*	QLQ-H&N35 Item numbers
**Symptom scales / items**				
Pain	HNPA	4	3	1 – 4
Swallowing	HNSW	4	3	5 – 8
Senses problems	HNSE	2	3	13,14
Speech problems	HNSP	3	3	16,23,24
Trouble with social eating	HNSO	4	3	19 – 22
Trouble with social contact	HNSC	5	3	18,25 – 28
Less sexuality	HNSX	2	3	29,30
Teeth	HNTE	1	3	9
Opening mouth	HNOM	1	3	10
Dry mouth	HNDR	1	3	11
Sticky saliva	HNSS	1	3	12
Coughing	HNCO	1	3	15
Felt ill	HNFI	1	3	17
Pain killers	HNPK	1	1	31
Nutritional supplements	HNNU	1	1	32
Feeding tube	HNFE	1	1	33
Weight loss	HNWL	1	1	34
Weight gain	HNWG	1	1	35

* “Item range” is the difference between the possible maximum and the minimum response to individual items.

Lung cancer module: QLQ-LC13

The lung cancer module is meant for use among a wide range of lung cancer patients varying in disease stage and treatment modality (Bergman *et al.*, 1994). The module comprises 13 questions (Appendix 2c). This module was constructed in parallel with the core QLQ-C30, before the guidelines on module development had been established. It was field tested together with the previous versions of the core questionnaire (QLQ-C36, QLQ-C30(V1)). The module is designed for use among patients receiving treatment with chemotherapy and / or radiotherapy. The QLQ-LC13 includes questions assessing lung cancer-associated symptoms (cough, haemoptysis, dyspnoea and site specific pain), treatment-related side effects (sore mouth, dysphagia, peripheral neuropathy and alopecia) and pain medication. The module was field tested together with the previous versions of the core questionnaire.

Scoring of the lung cancer module

The lung cancer module incorporates one multi-item scale to assess dyspnoea, and a series of single items assessing pain, coughing, sore mouth, dysphagia, peripheral neuropathy, alopecia, and haemoptysis.

The scoring approach for the QLQ-LC13 is identical in principle to that for the symptom scales / single items of the QLQ-C30.

Scale name	Scale	Number of items	Item range*	QLQ-LC13 Item numbers	†
**Symptom scales / items**					
Dyspnoea†	LCDY	3†	3	3,4,5	X
Coughing	LCCO	1	3	1	
Haemoptysis	LCHA	1	3	2	
Sore mouth	LCSM	1	3	6	
Dysphagia	LCDS	1	3	7	
Peripheral neuropathy	LCPN	1	3	8	
Alopecia	LCHR	1	3	9	
Pain in chest	LCPC	1	3	10	
Pain in arm or shoulder	LCPA	1	3	11	
Pain in other parts	LCPO	1	3	12	

* "Item range" is the difference between the possible maximum and the minimum response to individual items.

† The dyspnoea scale should only be used if all three items have been answered. Some respondents ignore question 5 because they never climb stairs; in this case, the score for the dyspnoea scale would be biased if it were based upon the other two items. Hence if item 5 is missing then items 3 and 4 should be used as single-item measures.

Oesophageal cancer module: QLQ-OES24

The oesophageal cancer module is designed for patients with local, locally advanced or metastatic disease treated with single or combination treatment including surgery, chemotherapy, radiotherapy or endoscopic palliation. It is also designed for patients receiving treatment with palliative intent (endoscopic stenting, laser, alcohol or diathermy ablation or palliative chemotherapy or radiotherapy). It was developed according to the EORTC Guidelines. It consists of 24 items assessing dysphagia, deglutition, abdominal/GI symptoms, eating difficulties, pain, emotional problems relating to oesophageal cancer and to side effects of chemotherapy/radiotherapy. The provisional module has been translated into Chinese, Danish, Dutch, Finnish, French, German, Italian, Norwegian, Polish, Spanish and Swedish.

Information about the development of the module (in 3 countries) is published (Blazeby *et al*., 1996, 2000). An international field study (EORTC Protocol 15961/40973) is underway to enable the psychometric properties of the instrument to be fully tested in a cross-cultural context.

Scoring of the oesophageal cancer module

The current scoring approach for the oesophageal cancer module is identical in principle to that used for the scales/items of the EORTC QLQ-C30. The scale structure proposed in the table below is based on a hypothesised scale structure. This will be confirmed after completion of the field study (2001).

Scale name	Number of items	Item range*	QLQ-OES24 Item numbers
**Scales / items**			
Dysphagia	3	3	1,2,3
Deglutition	2	3	4,5
Eating	4	3	6 – 9
GI symptoms	3	3	14,15,16
Pain	3	3	17,18,19
Emotional problems	4	3	20 – 23
Single items:	5	3	10 – 13,24

* "Item range" is the difference between the possible maximum and the minimum response to individual items.

Ovarian cancer module: QLQ-OV28

The ovarian cancer module is designed for patients with local or advanced disease who receive treatment by surgery with or without chemotherapy. It was developed according to the EORTC Guidelines. It consists of 28 items assessing abdominal/GI symptoms, peripheral neuropathy, other chemotherapy side effects, hormonal symptoms, body image, attitudes to disease/treatment, and sexuality. The provisional module has been translated into Danish, Dutch, French, German, Italian, Portuguese, Spanish and Swedish. Norwegian, Chinese and Russian translations are in preparation.

Preliminary scaling analysis has been conducted on the first 24 items of the module (i.e. excluding items on sexuality) using data from a multicentre chemotherapy trial in the UK (Cull *et al*., European Journal of Cancer in press). This report also includes information about the development of the module (in 6 countries) and preliminary evidence of its validity. An international field study (EORTC Protocol 15982) is underway to enable the psychometric properties of the full 28-item instrument to be more fully tested in a cross-cultural context.

Scoring of the ovarian cancer module

The scoring approach for the ovarian cancer module is identical in principle to that used for the scales/items of the EORTC QLQ-C30. The scale structure proposed in the table below is based on preliminary scaling analysis of the first 24 items of the module, i.e. excluding items 25-28 on sexuality (Cull *et al*., European Journal of Cancer in press).

Scale name	Number of items	Item range*	QLQ-OV28 Item numbers
**Symptom scales / items**			
Abdominal/GI	6	3	1 – 6
Peripheral neuropathy	2	3	11,12
Hormonal	2	3	18,19
Body image	2	3	20,21
Attitude to disease/treatment	3	3	22,23,24
Chemotherapy side effects	5	3	13 – 17
Other single items	4	3	7 – 10
(Sexuality – see below)			25 – 28

* "Item range" is the difference between the possible maximum and the minimum response to individual items.

The scaling performance of items 25-28 has yet to be established.

QLQ modules under development - phase III

The following modules are currently at an advanced stage of development (Phase III) by the EORTC Quality of Life Group. Those interested in using them should contact the: QL Coordinator, Quality of Life Unit, EORTC Data Center, Avenue E Mounier 83 - B11, 1200 Brussels, Belgium. Tel: +32 2 774 1611. Fax: +32 2 779 4568, Email: abo@eortc.be.

- **Bladder cancer modules: QLQ-BLsup24, QLQ-BLmi30**
  Two bladder cancer modules have been developed; a 24-item questionnaire for patients with superficial bladder cancer (Ta, T1, CIS), and a 30-item questionnaire for patients with muscle invasive bladder cancer (T2, T3, T4a and T4b). The two modules share a number of common items and scales, including those assessing urinary symptoms, bowel symptoms, and sexual functioning. The superficial bladder cancer module contains additional items assessing side effects of intravesical treatment (fever, malaise, convenience of and worry due to repeated cystoscopies). The muscle-invasive bladder cancer module contains additional items assessing urostomy problems, problems associated with the use of a catheter, and body image. These two modules are currently being translated into the major European languages. The field-testing of these modules will take place in the context of clinical trials of the EORTC Genito-Urinary Tract Cancer Cooperative Group, and other collaborating investigators.

- **Brain cancer module: QLQ-BN20**
  The brain cancer module is intended for patients undergoing chemotherapy or radiotherapy. It includes 20 items assessing future uncertainty, visual disorder, motor dysfunction, communication deficit and other disease symptoms (e.g. headaches and seizures) and treatment toxicities (e.g. hair loss) (Osoba *et al.*, 1996). A validation study has been performed with English-speaking patients from Canada, the UK and the USA.

- **Colorectal cancer module: QLQ-CR38**
  The colorectal cancer module is meant for use among a wide range of patients with colorectal cancer, varying in disease stage and treatment modality. The module comprises 38 questions assessing disease symptoms, side effects of treatment (sphincter-saving resection, rectum extirpation, radiotherapy and chemotherapy), body image, sexuality, and future perspective. All patients complete 19 questions, while the remaining questions are completed by subsamples of patients (males or females; patients with or without a stoma). The module has been developed according to the guidelines, and approved after formal review. A validation study in the Netherlands has been completed (Sprangers *et al.*, 1999). The module is currently being tested in a range of international phase III trials.

- **Gastric cancer module: QLQ-STO22**
  The gastric cancer module is meant for use among a wide range of patients with adenocarcinoma of the stomach. The module includes 22 items concerning disease and treatment-related symptoms and side effects, dysphagia, nutritional aspects and items about the emotional problems of gastric cancer. The module has been developed according to the guidelines, and approved after formal review. Pre-testing of the provisional module was performed on patients from the UK, France, Germany and Spain. An international field study is being launched.

- **Multiple myeloma module: QLQ-MY24**
  The myeloma module is designed for patients with multiple myeloma to assess the symptoms and side effects of treatment and their impact on everyday life (Stead *et al.*, 1999). The module comprises 24 questions addressing four domains of QoL important in myeloma: a pain scale, treatment side effects, social support and future perspective. The module was developed according to the guidelines, and approved after formal review. Pre-testing was carried out in the UK, Norway, Sweden, Denmark and Germany. The field-testing of the module is currently being organised.

- **Ophthalmic cancer module: QLQ-OPT37**
  The ophthalmic module is a 37-item questionnaire for use among patients with uveal melanoma. It includes subscales assessing vision impairment (9 items), functional problems due to vision impairment (8 items), eye symptoms (7 items), worry about recurrent disease (6 items), problems with appearance (3 items), problems driving (2 items), headache (1 item), problems reading (1 item). The pre-testing of the module will be conducted in UK, Finland and Sweden.

- **Pancreatic cancer module: QLQ-PAN26**
  The pancreatic cancer module is designed for patients at all disease stages undergoing surgical resection, palliative surgical intervention, endoscopic palliation or palliative chemotherapy (Fitzsimmons *et al.*, 1999a,b). The module comprises 26 questions assessing pain, dietary changes, jaundice, altered bowel habit, emotional problems related to pancreatic cancer, and other symptoms (cachexia, indigestion, flatulence, dry mouth, taste changes). Pre-testing of the provisional module was conducted in the UK, Sweden, Spain, Germany, Switzerland, France, Greece, Italy and Hungary. The module is currently being tested in international clinical trials and an international field study (EORTC protocol 15981) is due to be launched in 2001.

- **Prostate cancer module: QLQ-PR25**
  The prostate cancer module is a 25-item questionnaire designed for use among patients with localized and metastatic prostate cancer. It includes subscales assessing urinary symptoms (9 items), bowel symptoms (4 items), treatment-related symptoms (6 items) and sexual functioning (6 items). The module is currently available in English, Dutch and French, and is being translated into 15 European languages. The field-testing of the prostate cancer module is currently being organised.

- **Satisfaction with care module: QLQ-SAT32**
  The patient satisfaction module is intended to assess cancer patients' perception of the quality of care received during a hospital stay. It is composed of 32 items assessing doctors' and nurses' technical and interpersonal skills, information provision and availability; other hospital personnel kindness, helpfulness, and information giving, exchange of information between caregivers, waiting time (for performing medical tests/treatment, receiving medical tests results), access, comfort/cleanness, and general satisfaction. A validation study is planned with patients from oncology hospitals in France, Italy, The Netherlands, Sweden and the UK.

QLQ modules under development - phases I and II

The following modules are at earlier stages of development (Phases I and II). Those interested in obtaining information about their current status should contact the person named under the specific module.

- **Carcinoid module**
  Contact address: John Ramage, North Hampshire Hospital, Aldermaston Road, Basingstoke, Hampshire RG24 9NA, U.K. Tel: +44 1256 313 637; Fax: +44 1256 313 634; Email JohnRamage@compuserve.com
- **Choices and decision-making module**
  Contact address: Martin Eisemann, Unit of Medical Psychology, Dept. of Psychiatry & WHO Collaborative Center, Umea University, S-901 85 Umea, Sweden. Tel: +46 90 785 6320; Fax: +46 90 135 324; Email Martin.Eisemann@psychiat.umu.se
- **Fatigue module**
  Contact address: Joachim Weis, Tumor Biology Center, Breisacher Str. 117, D-79106 Freiburg, Germany. Tel: +49 761 206 2220; Fax: +49 761 206 2258; Email jowe@tumorbio.uni-freiburg.de
- **High-dose chemotherapy module**
  Contact address: Galina Velikova, ICRF Cancer Medicine Research Unit, St. James' University Hospital, Beckett Street, Leeds LS9 7TF, U.K. Tel: +44 113 20 66111; Fax: +44 113 20 66108; Email csjgv@leeds.ac.uk
- **Information module**
  Contact address: Juan Ignacio Arraras, Hospital of Navarre, Department of Oncology, Irunlarrea 3, 31008 Pamploma, Spain. Tel: +34 948 238 645; Fax: +34 948 422 303; Email jiarraras@correo.cop.es
- **Liver module**
  Contact address: Jane M Blazeby, Department of Surgery, Bristol Royal Infirmary, Bristol BS2 8HW, U.K. Tel: +44 117 9283153; Fax: +44 117 9252736; Email: jmblazeby@hotmail.com
- **Peripheral neuropathy module**
  Contact address: Neil Aaronson, Netherlands Cancer Institute, Department of Psychosocial Research and Epidemiology, Plesmanlaan 121, 1066 CX Amsterdam, The Netherlands. Tel: +31 20 5122480; Fax: +31 20 6172625; Email: naaron@nki.nl
- **Symptom check list**
  Contact address: Sam Ahmedzai, Palliative Medicine Dept., Division of Surgery & Anaesth. Sciences, University of Sheffield, Floor K, Royal Hallamshire Hospital, Sheffield S10 2JF, U.K. Tel: +44 114 271 2950; Fax: +44 271 3991; Email s.ahmedzai@sheffield.ac.uk

Translations

Translations

The translation of the core questionnaire and modules into other languages follows a procedure that has been documented in detail in the EORTC Quality of Life Group manual: *Translation Procedure*, Cull *et al*., 1998. Any modules not initially developed in English are first translated into English, and then to other languages. All translations involve two native speakers of the target language who are also fluent in the original language; these independently translate the questionnaire. The resultant translation is then "back translated" by two native speakers of the original language, with iterations between the forward and backward translation as necessary. There is then a pilot test of the translated questionnaire. A formal report is submitted to the Quality of Life Group, and this is reviewed by at least two members before approval is given.

The core questionnaire is available in many languages in addition to English, and translations of the QLQ supplementary modules are also available in various languages.

Currently available translations of QLQ-C30

At the time of going to press, the following translations of the EORTC QLQ-C30 exist. The original English version has been tested for use with British, American, Australian and Canadian speakers of English. An up to date list of translations of the QLQ-C30 and its modules may be obtained from the Quality of Life Unit at the EORTC Data Center.

Language	Gender	C30 (V1)	C30 (+3)	C30 (V2)	C30 (V3)
African (Sotho)					√
African (Xhosa)					√
African (Zulu)					√
Arabic					√
Bulgarian		√	√	√	√
Chinese			√	√	√
Chinese (Taiwanese)					+
Croatian				√	√
Czech	mixed	√	√	√	√
Danish		√	√	√	√
Dutch		√	√	√	√
Dutch (Afrikaans)				√	√
English		√	√	√	√
Finnish		√	√	√	√
French	m/f/mix	√	√	√	√
French (Canadian)					√
German		√	√	√	√
Greek	mixed	√	√	√	√
Hebrew		√		√	√
Hindi				+	
Hungarian		√	√	√	√
Indian (Gujarathi)					√
Indian (Marathi)					√
Iranian					√
Italian	m/f	√	√	√	√
Japanese				√	√
Korean					√
Lithuanian					√
Macedonian					√
Norwegian		√	√	√	√
Polish	mixed	√	√	√	√
Portuguese	mixed	√	√	√	√
Portuguese (Brazilian)	mixed			√	√
Romanian				√	
Russian		√	√	√	√
Serbian				√	√
Slovakian	mixed	√	√	√	√
Slovenian			√	√	√
Spanish	m/f	√	√	√	√
Spanish (American)	m/f	√	√	√	+
Spanish (Argentinian)	mixed			√	√
Swedish		√	√	√	√
Turkish		√	√	√	√

'm/f' indicates that separate male and female versions exist,
and 'mixed' indicates a version suitable for both sexes (neutral).
√ = available, + = pending or in preparation.

References

References

Aaronson NK, Ahmedzai S, Bergman B, *et al.* The European Organization for Research and Treatment of Cancer QLQ-C30: A quality-of-life instrument for use in international clinical trials in oncology. *Journal of the National Cancer Institute*. **85:** 365-76, 1993.

Aaronson NK, Ahmedzai S, Bullinger M, *et al.* The EORTC core quality of life questionnaire: interim results of an international field study. In: Osoba, D. (Ed.) *Effect of Cancer on Quality of Life*. CRC Press Ltd., Boca Raton :185-203, 1991.

Bergman B, Aaronson NK, Ahmedzai S, Kaasa S, Sullivan M. The EORTC QLQ-LC13: A modular supplement to the EORTC core quality of life questionnaire (QLQ-C30) for use in lung cancer clinical trials. *European Journal of Cancer*. **30A:** 635-42, 1994.

Bjordal K, Ahlner-Elmqvist M, Tollesson E, *et al.* Development of a European Organization for Research and Treatment of Cancer (EORTC) questionnaire module to be used in quality of life assessments in head and neck cancer patients. *Acta Oncologica*. **33:** 879-85, 1994.

Bjordal K, de Graeff A, Fayers PM, *et al.* A 12 country field study of the EORTC QLQ-C30 (version 3.0) and the head and neck cancer specific module (The EORTC QLQ-H&N35). *European Journal of Cancer*. **36:** 213-9, 2000.

Bjordal K, Hammerlid E, Ahlner-Elmqvist M, *et al.* Quality of life in head and neck cancer patients: validation of the European Organization for Research and Treatment of Cancer Quality of Life Questionnaire-H&N35. *Journal of Clinical Oncology*. **17:** 1008-19, 1999.

Bjordal K, Kaasa S. Psychometric validation of the EORTC core quality of life questionnaire, 30-item version and a diagnosis-specific module for head and neck cancer patients. *Acta Oncologica*. **31:** 311-21, 1992.

Blazeby JM, Alderson D, Farndon JR. Quality of life in patients with oesophageal cancer. *Recent Results in Cancer Research*. **155:** 193-204, 2000.

Blazeby JM, Alderson D, Winstone K, *et al.* Development of an EORTC questionnaire module to be used in quality of life assessment for patients with oesophageal cancer. The EORTC Quality of Life Study Group. *European Journal of Cancer*. **32A:** 1912-7, 1996.

Cox DR, Fitzpatrick R, Fletcher AE, Gore SM, Spiegelhalter DJ, Jones DR. Quality-of-life assessment: can we keep it simple? *Journal of the Royal Statistical Society*. *Series A*, **155:** 353-93, 1992.

Cox DR, Wermuth N. Tests of linearity, multivariate normality and the adequacy of linear scores. *Applied Statistics*. **43:** 347-55, 1994.

Cull A, Sprangers M, Bjordal K, Aaronson N, on behalf of the EORTC Quality of Life Study Group. *EORTC Quality of Life Study Group Translation Procedure*. EORTC, Brussels, 1998. ISBN: 2-930064-15-3.

Dawes RM. The robust beauty of improper linear models. *American Psychologist*. **34:** 571-82, 1979.

Drummond MF, O'Brien B, Stoddart GL. *Methods for the Economic Evaluation of Health Care Programmes*. Oxford University Press, Oxford, 1997. ISBN: 0-19262-774-0.

Fayers PM, Machin D. *Quality of Life: Assessment, Analysis and Interpretation*. J Wiley & Sons Ltd, Chichester, 2000. ISBN: 0-471-96861-7.

Fayers PM, Weeden S, Curran D, on behalf of the EORTC Quality of Life Study Group. *EORTC QLQ-C30 Reference Values*. EORTC, Brussels, 1998. ISBN: 2-930064-11-0.

Fitzsimmons D, George S, Payne S, Johnson CD. Differences in perception of quality of life issues between health professionals and patients with pancreatic cancer. *Psycho Oncology*. **8:** 135-43, 1999b.

Fitzsimmons D, Johnson CD, George S, *et al.* Development of a disease specific quality of life (QoL) questionnaire module to supplement the EORTC core cancer QoL questionnaire, the QLQ-C30 in patients with pancreatic cancer. *European Journal of Cancer*. **35:** 939-41, 1999a.

Gorsuch RL. *Factor Analysis*. 2nd ed. Lawrence Erlbaum, Hillsdale, N.J., 1983. ISBN: 0-89859-202-X.

Hjermstad MJ, Fayers PM, Bjordal K, Kaasa S. Health-related quality of life in the general Norwegian population assessed by the European Organization for Research and Treatment of Cancer Core Quality-of-Life Questionnaire: The QLQ-C30 (+3). *Journal of Clinical Oncology*. **16:** 1188-96, 1998.

King MT. The interpretation of scores from the EORTC quality of life questionnaire QLQ-C30. *Quality of Life Research*. **5:** 555-67, 1996.

Klee M, Groenvold M, Machin D. Quality of life of Danish women: Population-based norms for the EORTC QLQ-C30. *Quality of Life Research*. **6:** 27-34, 1997.

Lydick E, Epstein RS. Interpretation of quality of life changes. *Quality of Life Research*. **2:** 221-6, 1993.

Michelson H, Bolund C, Nilsson B, Brandburg Y. Health-related quality of life measured by the EORTC QLQ-C30. Reference values from a large sample of the Sweedish population. *Acta Oncologica*. **39:** 477-84, 2000.

Olschewski M, Schumacher M. Statistical analysis of quality of life data in cancer clinical trials. *Statistics in Medicine*. **9:** 749-63, 1990.

Osoba D, Aaronson NK, Muller M, *et al.* The development and psychometric validation of a brain cancer quality of life questionnaire for use in combination with general cancer-specific questionnaires. *Quality of Life Research*. **5:** 139-50, 1996.

Osoba D, Aaronson NK, Zee B, Sprangers MAG, te Velde A. Modification of the EORTC QLQ-C30 (version 2.0) based upon content validity and reliability testing in large samples of patients with cancer. *Quality of Life Research*. **6:** 103-8, 1997.

Osoba D, Rodrigues G, Myles J, Zee B, Pater J. Interpreting the significance of changes in health-related quality-of-life scores. *Journal of Clinical Oncology*. **16:** 139-44, 1998.

Simes RJ. Application of statistical decision theory to treatment choices: implications for the design and analysis of clinical trials. *Statistics in Medicine*. **5:** 411-20, 1986.

Sprangers MA, Cull A, Groenvold M, Bjordal K, Blazeby J, Aaronson NK. The European Organization for Research and Treatment of Cancer approach to developing questionnaire modules: an update and overview. EORTC Quality of Life Study Group. *Quality of Life Research*. **7:** 291-300, 1998a.

Sprangers M, Cull A, Groenvold M, on behalf of the EORTC Quality of Life Study Group. *EORTC Quality of Life Study Group Guidelines for Developing Questionnaire Modules*. EORTC, Brussels, 1998b. ISBN: 2-930064-10-2.

Sprangers MAG, Groenvold M, Arraras JI, *et al.* The EORTC breast cancer-specific quality-of-life questionnaire module: first results from a three-country field study. *Journal of Clinical Oncology*. **14:** 2756-68, 1996.

Sprangers MAG, Te Velde A, Aaronson NK. The construction and testing of the EORTC colorectal cancer-specific quality of life questionnaire module (QLQ-CR38). *European Journal of Cancer*. **35:** 238-47, 1999.

Stead ML, Brown JM, Velikova G, *et al.* Development of an EORTC questionnaire module to be used in health-related quality-of-life assessment for patients with multiple myeloma. European Organization for Research and Treatment of Cancer Study Group on Quality of Life. *British Journal of Haematology*. **104:** 605-11, 1999.

Troxel AB, Fairclough DL, Curran D, Hahn EA. Statistical analysis of quality of life data in cancer clinical trials. *Statistics in Medicine*. **17:** 653-66, 1998.

Young T, de Haes JCJM, Curran D, Fayers PM, Brandberg Y, on behalf of the EORTC Quality of Life Study Group. *Guidelines for Assessing Quality of Life in EORTC Clinical Trials*. EORTC, Brussels, 1999. ISBN: 2-930064-17-X.

Glossary

Glossary

Core questionnaire:

The QLQ-C30 is a "core questionnaire" which incorporates a range of physical, emotional and social health issues relevant to a broad spectrum of cancer patients; the core questionnaire should be used, unmodified, in all QLQ assessments.

Items:

The individual questions on the QLQ-C30 are called items, and are described as Q_1, Q_2, Q_3, etc., where the suffix corresponds to the question-number on the QLQ questionnaire.

Module:

The core questionnaire may be supplemented by diagnosis-specific and/or treatment-specific questionnaire modules. Modules should be used unmodified and in conjunction with the core questionnaire.

Raw score:

The score formed by averaging the items that are included in a particular function or symptom scale.

Scales:

The QLQ comprises distinct scales, each of which represents a different aspect of QoL.

Scale score:

The raw score transformed to a standardised 0 - 100 final "scale score".

Appendix 1

QLQ-C30

1a: QLQ-C30 version 1.0

1b: QLQ-C30 (+3)

1c: QLQ-C30 version 2.0

1d: QLQ-C30 version 3.0

Appendix 1a: QLQ-C30 version 1.0

Error! Not a valid link.

Appendix 1a: QLQ-C30 version 1.0

Error! Not a valid link.

Appendix 1b: QLQ-C30 (+3)

Error! Not a valid link.

Appendix 1b: QLQ-C30 (+3)

Error! Not a valid link.

Appendix 1c: QLQ-C30 version 2.0

Error! Not a valid link.

Appendix 1c: QLQ-C30 version 2.0

Error! Not a valid link.

Appendix 1d: QLQ-C30 version 3.0

Error! Not a valid link.

Appendix 1d: QLQ-C30 version 3.0

Error! Not a valid link.

Appendix 2

QLQ Modules

2a: Breast cancer module

2b: Head & Neck cancer module

2c: Lung cancer module

2d: Oesophageal cancer module

2e: Ovarian cancer module

Appendix 2a: Breast cancer module QLQ-BR23

Error! Not a valid link.

Appendix 2a: Breast cancer module QLQ-BR23

Error! Not a valid link.

Appendix 2b: Head & Neck cancer module QLQ-H&N35

Error! Not a valid link.

Appendix 2b: Head & Neck cancer module   QLQ-H&N35

Error! Not a valid link.

Appendix 2c: Lung cancer module   QLQ-LC13

Error! Not a valid link.

Appendix 2d: Oesophageal cancer module QLQ-OES24

Error! Not a valid link.

Appendix 2d: Oesophageal cancer module QLQ-OES24

Error! Not a valid link.

Appendix 2e: Ovarian cancer module QLQ-OV28

Error! Not a valid link.

Appendix 2e: Ovarian cancer module QLQ-OV28

Error! Not a valid link.

Appendix 3

Coding for Statistical Packages

3a: SAS

3b: SPSS

3c: STATA

Appendix 3a: SAS program for scoring the QLQ-C30 and supplementary modules

Installation

To run the commands, first load your data set into SAS.

Then copy these SAS syntax files to the "sas\core\sasmacro" directory and use the "%include" statement before running the SAS macros, e.g.

```
%include 'c:\sas\core\sasmacro\score.sas';
%include 'c:\sas\core\sasmacro\qlscore.sas';
```

Variable names

The QLQ-C30 variables should be named q1, q2, q3, ... q30 (or up to q33 if using the QLQ-C30+3). If analysing data for the QLQ-BR23, H&N35 or QLQ-LC13, their variables should be named br1, br2, ...; hn1, hn2, ...; or lc1, lc2, ... respectively.

How to use

A display screen will appear in SAS asking you to provide the name of your data set.
Type in the name of your data set without specifying its location, e.g. mydata.
A second screen will ask you to type in the version of the QLQ-C30 that you used.
A third display screen allows you to specify if you would like to score an additional validated EORTC module. Finally, in a 4th screen you can specify the name of the data set containing the data for the BR23, H&N35 and LC13 modules.

What you get

Function scales, symptom scales and single-item scales are calculated and labelled in accordance with the descriptions in the QLQ-C30 Scoring Manual. For example, the emotional functioning scale is calculated from items q21, q22, q23, q24. It is called EF, and is labelled "Emotional functioning". It is scaled to range from 0 to 100, with a high score indicating high emotional functioning.

Note that if you have an existing variable in your data set called EF, it will be automatically over-written. If this is not what you want, you should copy the original data set and store it under a different name for this application: your revised data set will automatically be copied into your original SAS data set(s).

Out-of-range values

This program checks for items with out-of-range values and treats them as missing.

Missing items

The default action of these programs is to impute missing items provided at least half the scale items are present; the method is described in the Scoring Manual.

Writing your own modules

If you wish to adapt this file for handling items and scales in new QoL items and scales that you develop, it is very easy. Modules %add1, %add2, %add3, were developed for the LC13, BR23 and H&N35, respectively. Thus, all you need to do is prepare a similar macro for your items and scales. This macro can then be run separately, e.g. %add4 (data=mydata)

SAS program for scoring the QLQ-C30 and Modules

```
/* SAS COMMANDS to generate QLQ scales. */
/* ==================================== */

/* Kristel Van Steen and Desmond Curran, 30 November, 1998                */

/*======================================================================*/
/* This code is provided as is without warranty of any kind, either       */
/* expressed or implied.  It is not guaranteed to be error free, and in   */
/* no event will we accept liability for any damages arising out of the   */
/* use of this code.                                                      */
/*======================================================================*/

/**********************/
/* Cleaning the Data */
/**********************/

%macro clean(data,version);

proc format;
value item2_ 1='No'
             2='Yes';
value item4_ 1='Not at All'
             2='A Little'
             3='Quite a Bit'
             4='Very Much';
value item7_ 1='Very Poor'
             7='Excellent';

data &data;
        set &data;
        %if (&version=1 or &version=33 or &version=2) %then %do;
           %do I=1 %to 5;
              if (q&i<1 or q&i>2) then q&i=.;
              format q&i item2_.;
           %end;
        %end;
        %if (&version=3) %then %do;
          %do I=1 %to 5;
            if (q&i<1 or q&i>4) then q&i=.;
            format q&i item4_.;
          %end;
        %end;
        %if (&version=1 or &version=33) %then %do;
          %do I=6 %to 7;
            if (q&i<1 or q&i>2) then q&i=.;
            format q&i item2_.;
          %end;
        %end;
        %if (&version=2 or &version=3) %then %do;
          %do I=6 %to 7;
            if (q&i<1 or q&i>4) then q&i=.;
            format q&i item4_.;
          %end;
        %end;
        %do I=8 %to 28;
          if (q&i<1 or q&i>4) then q&i=.;
          format q&i item4_.;
        %end;
        %if (&version=1 or &version=2 or &version=3) %then %do;
          %do I=29 %to 30;
            if (q&i<1 or q&i>7) then q&i=.;
            format q&i item7_.;
          %end;
        %end;
        %if (&version=33) %then %do;
          %do I=29 %to 30;
            if (q&i<1 or q&i>4) then q&i=.;
            format q&i item4_.;
          %end;
        %end;
        %if (&version=33) %then %do;
          %do I=31 %to 33;
            if (q&i<1 or q&i>7) then q&i=.;
            format q&i item7_.;
          %end;
        %end;
run;

%mend clean;


/*************************************/
/* Definitions for Score Calculation */
/*************************************/

%macro score(data,type,scale,items);
%let i=1;
  %do %while(%length(%scan(&items,&i)) ^= 0);
      %let i=%eval(&i+1);
  %end;
%let i=%eval(&i-1) ;

data &data;
     set &data;
       XMEAN = MEAN(OF &items);
       XNUM    = N (of &items);
       %if (%upcase(&type)=F) %then %do ;
        If (XNUM*2 GE &i) THEN &scale = ((1-(XMEAN-1)/3)*100);
       %end ;
       %if (%upcase(&type)=G) %then %do ;
        If (XNUM*2 GE &i) THEN &scale = (((XMEAN-1)/6)*100);
       %end ;
       %if (%upcase(&type)=S) %then %do ;
       %if (&items= hn31 or &items= hn32 or &items= hn33 or
             &items= hn34 or &items= hn35) %then %do ;
             If (XNUM*2 GE &i) THEN &scale = ((XMEAN-1)*100);
          %end ;
          %else %do ;
             If (XNUM*2 GE &i) THEN &scale = (((XMEAN-1)/3)*100);
          %end ;
       %end ;

       %if (%upcase(&scale)=PF) %then %do ;
        if ((&version=1 or &version=33 or &version=2)
        and (XNUM*2 GE &i)) THEN &scale = ((1-(XMEAN-1))*100);
       %end;
       %if (%upcase(&scale)=RF) %then %do ;
```

```
      if ((&version=1 or &version=33) and (XNUM*2 GE &i))
         THEN &scale = ((1-(XMEAN-1))*100);
     %end;

run;

%mend score;


/**************************************************************/
/* Returning a Data Set with Calculated Scores for Each Scale:*/
/* Basic Macro                                                */
/**************************************************************/

%macro qcscore(data,version);

        %clean(&data,&version) ;

        %score(data=&data , type=f, scale=pf, items= q1 q2 q3 q4 q5);
        %score(data=&data , type=f, scale=rf, items= q6 q7);
        %if (&version=33) %then %do;
          %score(data=&data , type=f, scale=rf2, items= q26 q27);
        %end;
        %score(data=&data , type=f, scale=ef, items= q21 q22 q23 q24);
        %score(data=&data , type=f, scale=cf, items= q20 q25);
        %if (&version=1 or &version=2 or &version=3) %then %do;
          %score(data=&data , type=f, scale=sf, items= q26 q27);
        %end;
        %if (&version=33) %then %do;
          %score(data=&data , type=f, scale=sf, items= q28 q29);
        %end;
        %if (&version=1 or &version=2 or &version=3) %then %do;
          %score(data=&data , type=g, scale=ql, items= q29 q30);
        %end;
        %if (&version=33) %then %do;
          %score(data=&data , type=g, scale=ql, items= q31 q33);
          %score(data=&data , type=g, scale=ql2, items= q32 q33);
        %end;
        %score(data=&data , type=s, scale=fa, items= q10 q12 q18);
        %score(data=&data , type=s, scale=nv, items= q14 q15);
        %score(data=&data , type=s, scale=pa, items= q9 q19);
        %score(data=&data , type=s, scale=dy, items= q8);
        %score(data=&data , type=s, scale=sl, items= q11);
        %score(data=&data , type=s, scale=ap, items= q13);
        %score(data=&data , type=s, scale=co, items= q16);
        %score(data=&data , type=s, scale=di, items= q17);
        %if (&version=1 or &version=2 or &version=3) %then %do;
        %score(data=&data , type=s, scale=fi, items= q28);
        %end;
        %if (&version=33) %then %do;
          %score(data=&data , type=s, scale=fi, items= q30);
        %end;


        Data &data;
        set &data ;
        LABEL q1='STRENUOUS ACTIVITIES';
        LABEL q2='LONG WALK';
        LABEL q3='SHORT WALK';
        LABEL q4='IN BED';
        LABEL q5='NEED HELP';

        if  &version=1 or &version=33 then do;
          LABEL q6='LIMITED JOBS';
          LABEL q7='UNABLE TO WORK';
        end;
        if  &version=2 or &version=3 then do;
          LABEL q6='LIMITED ACTIVITY';
          LABEL q7='LIMITED HOBBIES';
        end;
        LABEL q8='SHORT OF BREATH';
        LABEL q9='PAIN';
        LABEL q10='NEED REST';
        LABEL q11='TROUBLE SLEEPING';
        LABEL q12='FELT WEAK';
        LABEL q13='LACKED APPETITE';
        LABEL q14='FELT NAUSEATED';
        LABEL q15='VOMITED';
        LABEL q16='BEEN CONSTIPATED';
        LABEL q17='HAD DIARRHEA';
        LABEL q18='BEEN TIRED';
        LABEL q19='PAIN INTERFERENCE';
        LABEL q20='DIFFICULTY CONCENTRATING';
        LABEL q21='FELT TENSE';
        LABEL q22='WORRIED';
        LABEL q23='FELT IRRITABLE';
        LABEL q24='FELT DEPRESSED';
        LABEL q25='MEMORY';
        if (&version=1) then do;
          LABEL q26='FAMILY LIFE';
          LABEL q27='SOCIAL ACTIVITIES';
          LABEL q28='FINANCIAL DIFFICULTIES';
          LABEL q29='PHYSICAL CONDITION';
          LABEL q30='QUALITY OF LIFE';
        end;
        if (&version=33) then do;
          LABEL q26='LIMITED ACTIVITIES';
          LABEL q27='LIMITED HOBBIES';
          LABEL q28='FAMILY LIFE';
          LABEL q29='SOCIAL ACTIVITIES';
          LABEL q30='FINANCIAL DIFFICULTIES';
          LABEL q31='PHYSICAL CONDITION';
          LABEL q32='OVERALL HEALTH';
          LABEL q33='QUALITY OF LIFE';
        end;
        if (&version=2 or &version=3) then do;
          LABEL q26='FAMILY LIFE';
          LABEL q27='SOCIAL ACTIVITIES';
          LABEL q28='FINANCIAL DIFFICULTIES';
          LABEL q29='OVERALL HEALTH';
          LABEL q30='QUALITY OF LIFE';
        end;

        LABEL pf='Physical Functioning';
        LABEL rf='Role Functioning';
        LABEL rf2='Role Functioning';
        LABEL ef='Emotional Functioning';
        LABEL cf='Cognitive Functioning';
        LABEL sf='Social Functioning';
        LABEL ql='Global health status / QoL';
```

```
        LABEL ql2='Global health status / QoL';
        LABEL fa='Fatigue';
        LABEL nv='Nausea / Vomiting';
        LABEL pa='Pain';
        LABEL dy='Dyspnoea';
        LABEL sl='Insomnia';
        LABEL ap='Appetite loss';
        LABEL co='Constipation';
        LABEL di='Diarhoea';
        LABEL fi='Financial Problems';

        drop xnum xmean ;
        run ;

%mend qcscore;


/***********************/
/* Lung Cancer module */
/***********************/


%macro add1(mdata);

        data &mdata ;
          set &mdata ;
          %do i=1 %to 12 ;
             if (lc&i<1 or lc&i>4) then lc&i=. ;
             format lc&i item4_. ;
          %end;
        run;

        %score(data=&mdata,type=S,scale=lcco,items = lc1) ;
        %score(data=&mdata,type=S,scale=lcha,items = lc2) ;
        %score(data=&mdata,type=S,scale=lcsm,items = lc6) ;
        %score(data=&mdata,type=S,scale=lcds,items = lc7) ;
        %score(data=&mdata,type=S,scale=lcpn,items = lc8) ;
        %score(data=&mdata,type=S,scale=lchr,items = lc9) ;
        %score(data=&mdata,type=S,scale=lcpc,items = lc10) ;
        %score(data=&mdata,type=S,scale=lcpa,items = lc11) ;
        %score(data=&mdata,type=S,scale=lcpo,items = lc12) ;

    data &mdata ;
          set &mdata ;
        if (lc3^=. and lc4^=. and lc5^=.) then do;
        xmean = mean(of lc3 lc4 lc5);
        lcdy= (((xmean-1)/3)*100);
    end;

          *Symptom scales
          label lcdy= 'LC DYSPNOEA' ;
          label lcco= 'LC COUGHING' ;
          label lcha= 'LC HAEMOPTYSIS' ;
          label lcsm= 'LC SORE MOUTH' ;
          label lcds= 'LC DYSPHAGIA' ;
          label lcpn= 'LC PERIPH NEUROPATHY' ;
          label lchr= 'LC ALOPECIA' ;
          label lcpc= 'LC PAIN IN CHEST' ;
          label lcpa= 'LC PAIN IN ARM' ;
          label lcpo= 'LC PAIN OTHER' ;

          *Additional variables
          label lc1='LC COUGHING' ;
          label lc2='LC HAEMOPTYSIS' ;
          label lc3='LC DYSPNOEA/RESTED' ;
          label lc4='LC DYSPNOEA/WALKED' ;
          label lc5='LC DYSPNOEA/STAIRS' ;
          label lc6='LC SORE MOUTH' ;
          label lc7='LC DYSPHAGIA' ;
          label lc8='LC PERIPH NEUROPATHY' ;
          label lc9='LC ALOPECIA' ;
          label lc10='LC PAIN IN CHEST' ;
          label lc11='LC PAIN IN ARM' ;
          label lc12='LC PAIN OTHER' ;

          drop xnum xmean ;
        run ;

%mend add1 ;


/************************/
/* Breast Cancer module */
/************************/


%macro add2(mdata);

        data &mdata ;
          set &mdata ;
          %do i=1 %to 23 ;
             if (br&i<1 or br&i>4) then br&i=. ;
             format br&i item4_. ;
          %end;
        run;

        %score(data=&mdata,type=F,scale=brbi,items = br9 br10 br11 br12) ;
        %score(data=&mdata,type=S,scale=brsef,items = br14 br15) ;
        %score(data=&mdata,type=F,scale=brfu,items = br13) ;
   %score(data=&mdata,type=S,scale=brct,items = br1 br2 br3 br4 br6 br7 br8);
        %score(data=&mdata,type=S,scale=brrt,items = br20 br21 br22 br23) ;
        %score(data=&mdata,type=S,scale=brsy,items = br17 br18 br19 ) ;

        data &mdata ;
          set &mdata ;

          %if (br15 ^= 1 and br15 ^= .)
                %then %score(data=&mdata,type=S,scale=brsee,items = br16) ;
                %else br16 = . ;
        %score(data=&mdata,type=S,scale=brhl,items = br5) ;
        %if (br4 = 1) %then brhl = 0 ;

          *Function scales;
          label brbi=  'BR BODY IMAGE' ;
          label brsef= 'BR SEXUAL FUNCTIONING' ;
          label brsee= 'BR SEXUAL ENJOYMENT' ;
          label brfu= 'BR FUTURE PERSPECTIVE' ;

          *Symptom scales;
          label brct= 'BR SYSTEMATIC THERAPY' ;
```

```
        label brrt= 'BR BREAST SYMPTOMS' ;
        label brsy= 'BR ARM SYMPTOMS' ;
        label brhl= 'BR HAIR LOSS' ;

        *Additional variables;
        label br9= 'BR ATTRACTIVENESS' ;
        label br10='BR LESS FEMININE' ;
        label br11='BR NAKEDNESS' ;
        label br12='BR BODY DISSATISF' ;
        label br14='BR INTERESTED IN SEX' ;
        label br15='BR SEXUAL ACTIVITY' ;
        label br16='BR SEXUAL ENJOYMENT' ;
        label br1= 'BR DRY MOUTH' ;
        label br2= 'BR TASTE' ;
        label br3= 'BR EYES' ;
        label br4= 'BR LOST HAIR' ;
        label br6= 'BR FELT ILL/UNWELL' ;
        label br7= 'BR HOT FLUSHES' ;
        label br8= 'BR HEADACHES' ;
        label br20='BR PAINFUL AREA' ;
        label br21='BR SWOLLEN AREA' ;
        label br22='BR OVERSENSITIVE AREA' ;
        label br23='BR SKIN PROBLEMS' ;
        label br17='BR PAIN IN ARM' ;
        label br18='BR SWOLLEN ARM' ;
        label br19='BR ARM MOVEMENTS' ;
        label br5= 'BR UPSET/HAIR LOSS' ;
        label br13='BR FUTURE PERSPECTIVE' ;

        drop xnum xmean ;
      run ;

%mend add2 ;


/********************************/
/* Head and Neck Cancer module */
/********************************/

%macro add3(mdata);

      data &mdata ;
        set &mdata ;
        %do i=1 %to 30 ;
          if (hn&i<1 or hn&i>4) then hn&i=. ;
          format hn&i item4_. ;
        %end;
        %do j=31 %to 35 ;
          if (hn&j<1 or hn&j>2) then hn&j=. ;
          format hn&j item2_. ;
        %end;
      run;

    %score(data=&mdata,type=S,scale=hnpa,items = hn1 hn2 hn3 hn4) ;
    %score(data=&mdata,type=S,scale=hnsw,items = hn5 hn6 hn7 hn8) ;
    %score(data=&mdata,type=S,scale=hnse,items = hn13 hn14) ;
    %score(data=&mdata,type=S,scale=hnsp,items = hn16 hn23 hn24) ;
    %score(data=&mdata,type=S,scale=hnso,items = hn19 hn20 hn21 hn22) ;
    %score(data=&mdata,type=S,scale=hnsc,items = hn18 hn25 hn26 hn27 hn28)
;
    %score(data=&mdata,type=S,scale=hnsx,items = hn29 hn30) ;

    %score(data=&mdata,type=S,scale=hnte,items = hn9) ;
    %score(data=&mdata,type=S,scale=hnom,items = hn10) ;
    %score(data=&mdata,type=S,scale=hndr,items = hn11) ;
    %score(data=&mdata,type=S,scale=hnss,items = hn12) ;
    %score(data=&mdata,type=S,scale=hnco,items = hn15) ;
    %score(data=&mdata,type=S,scale=hnfi,items = hn17) ;

    %score(data=&mdata,type=S,scale=hnpk,items = hn31) ;
    %score(data=&mdata,type=S,scale=hnnu,items = hn32) ;
    %score(data=&mdata,type=S,scale=hnfe,items = hn33) ;
    %score(data=&mdata,type=S,scale=hnwl,items = hn34) ;
    %score(data=&mdata,type=S,scale=hnwg,items = hn35) ;


      data &mdata ;
        set &mdata ;

        *Symptom scales;
        label hnpa= 'HN PAIN' ;
        label hnsw= 'HN SWALLOWING' ;
        label hnse= 'HN SENSES' ;
        label hnsp= 'HN SPEECH' ;
        label hnso= 'HN SOCIAL EATING' ;
        label hnsc= 'HN SOCIAL CONTACT' ;
        label hnsx= 'HN SEXUALITY' ;
        label hnte= 'HN TEETH' ;
        label hnom= 'HN OPENING MOUTH' ;
        label hndr= 'HN DRY MOUTH' ;
        label hnss= 'HN STICKY SALIVA' ;
        label hnco= 'HN COUGHED' ;
        label hnfi= 'HN FELT ILL' ;
        label hnpk= 'HN PAIN KILLERS' ;
        label hnnu= 'HN NUTRITIONAL SUPP' ;
        label hnfe= 'HN FEEDING TUBE' ;
        label hnwl= 'HN WEIGHT LOSS'  ;
        label hnwg= 'HN WEIGHT GAIN'  ;

        *Additional variables;
        label hn1=  'HN PAIN IN MOUTH' ;
        label hn2=  'HN PAIN IN JAW' ;
        label hn3=  'HN SORE MOUTH' ;
        label hn4=  'HN PAINFUL THROAT' ;
        label hn5=  'HN SWALLOW LIQUIDS' ;
        label hn6=  'HN SWALLOW PUREED FOOD' ;
        label hn7=  'HN SWALLOW SOLID FOOD' ;
        label hn8=  'HN SWALLOW AND CHOKE' ;
        label hn13= 'HN SENSE OF SMELL' ;
        label hn14= 'HN SENSE OF TASTE' ;
        label hn16= 'HN HOARSE' ;
        label hn23= 'HN TALKING TO PEOPLE' ;
        label hn24= 'HN TALKING ON PHONE' ;
        label hn19= 'HN EATING' ;
        label hn20= 'HN EATING WITH FAMILY' ;
        label hn21= 'HN EATING WITH OTHERS' ;
        label hn22= 'HN ENJOYED MEAL' ;
        label hn18= 'HN APPEARANCE' ;
        label hn25= 'HN HOUSEHOLD' ;
        label hn26= 'HN HOBBIES' ;
```

```
   label hn27= 'HN SOCIALIZING/FAMILY';
   label hn28= 'HN SOCIALIZING/FRIENDS' ;
   label hn29= 'HN GOING OUT' ;
   label hn30= 'HN PHYSICAL CONTACT' ;
   label hn9=  'HN TEETH' ;
   label hn10= 'HN OPENING MOUTH' ;
   label hn11= 'HN DRY MOUTH' ;
   label hn12= 'HN STICKY SALIVA' ;
   label hn15= 'HN COUGHED' ;
   label hn17= 'HN FELT ILL' ;
   label hn31= 'HN PAIN KILLERS' ;
   label hn32= 'HN NUTRITIAL SUPP' ;
   label hn33= 'HN FEEDING TUBE' ;
   label hn34= 'HN WEIGHT LOSS' ;
   label hn35= 'HN WEIGHT GAIN' ;

   drop xnum xmean ;
run ;

%mend add3 ;

/***************************************/
/* Basic macro for additional modules */
/***************************************/

%macro addendum(mdata,mod) ;

        %if &mod=LC %then %add1(&mdata) ;
        %if &mod=BR %then %add2(&mdata) ;
        %if &mod=HN %then %add3(&mdata) ;

%mend addendum ;
```

Appendix 3b: SPSS program for scoring the QLQ-C30 and supplementary modules

Installation

The easiest way to use this SPSS syntax file is to copy it into the same directory as your SPSS data set (for example, the same directory as your `SPSS .SAV` file).

How to use

The QLQ-C30 variables should be named `q1`, `q2`, `q3`, ... `q30` (or up to `q33` if using the QLQ-C30(+3)).
If analysing data for the QLQ-BR23, QLQ-H&N35 or QLQ-LC13, their variables should be named `br1`, `br2`, ...; `hn1`, `hn2`, ...; or `lc1`, `lc2`, ... respectively.

To run the commands, first load your data set into SPSS.
Either use the Windows interface to do this, or type for example:

```
GET FILE <your .SAV data set>.
```

Then load this SPSS syntax file into the syntax editor window.
At the end of this file, type one of the following commands as appropriate, according to the version of the QLQ-C30 that you used (V3, V2, V1, or (+3) respectively):

```
qlqscal 3 .
qlqscal 2.
qlqscal 1.
qlqscal 303.
```

For the BR23, H&N35 and LC13 modules the corresponding commands are:

```
brscal.
hnscal.
lcscal.
```

Finally, terminate with `EXECUTE.` and run the commands.

An alternative method is to load the `QLQ.sps` syntax file into the syntax editor, run all the commands, and then in a new syntax editor window type and run, for example,

`GET FILE TEST.SAV.` (this example assumes your data set is called `TEST.SAV`)

```
qlqscal 1.
EXECUTE.
```

What you get

Function scales, symptom scales and single-item scales are calculated and labelled in accordance with the descriptions in the QLQ-C30 Scoring Manual. For example, the emotional functioning scale is calculated from items q21, q22, q23, q24. It is called EF, and is labelled "Emotional functioning". It is scaled to range from 0 to 100, with a high score indicating high emotional functioning.

Note that if you have an existing variable in your data set called EF, it will be automatically over-written. If this is not what you want, you should take care ***not*** to save your revised data set into your original file on disk when you quit from SPSS.

Out-of-range values

This program sets out-of-range values to SPSS "user-missing".
If you have used special codes to indicate not applicable or other values,
you can cancel the user-missing codes by entering

MISSING VALUES q1 TO q30 ().

(and similarly for the QLQ supplementary modules).

Missing items

The default action of these programs is to impute missing items provided at least half the scale items are present; the method is described in the Scoring Manual. This imputation algorithm accounts for part of the complexity of the code in `qlqsub`.

You can over-ride this default action. To cause the presence of any missing values in the scale items to result in their scale score being missing, enter `XMISS` after the command. For example,

qlqscal 1 XMISS.

Writing your own modules

If you wish to adapt this file for handling items and scales in new QoL items or scales that you develop, it is very easy.

The files programs brscal, hnscal and lcscal all correspond directly to the tables given in the QLQ-C30 Scoring Manual, and serve as examples. Thus, all you need to do is prepare a similar table for your own items or scales, and copy the contents of the table into your own file. This can then be edited to use the identical syntax to the examples.

Make sure that the header and final "`!enddefine .`" lines also follow the example, with the name on the "`define`" command being the name that you call to run the program.

That's all – it’s simple!

SPSS program for scoring the QLQ-C30 and Modules

```
* .
* SPSS COMMANDS to generate QLQ scales.
* ===================================.
* .
* SPSS version 7.5 .
/* Peter Fayers,  30 August, 1998                                     */

/*====================================================================*/.
/* This code is provided as is without warranty of any kind, either   */
/* expressed or implied.  It is not guaranteed to be error free, and in   */
/* no event will we accept liability for any damages arising out of the   */
/* use of this code.                                                  */
/*====================================================================*/.

define  qlqsub ( !POSITIONAL !TOKENS(1)
               /!POSITIONAL !TOKENS(1)
               /!POSITIONAL !TOKENS(1)
               /!POSITIONAL !TOKENS(1)
               /!POSITIONAL !ENCLOSE('(',')')
               /!POSITIONAL !CMDEND).
/*====================================================================*/
/*   qlqsub is the utility routine called by the SPSS programs that   */
/*        calculate scores for the QLQ-C30 and its modules.           */
/*                                                                    */
/* ARGUMENTS                                                          */
/*  Name of scale           !SCALE                                    */
/*  Description of scale    !SCALNAM                                  */
/*  Number of items in scale !NITEMS                                   */
/*  Range of each item in the scale !IRANGE                           */
/*  List of items in the scale (!QLQVARS)                             */
/*  (Optional) string FSCALE - indicates function scales (highscore=good) */
/*  (Optional) string XMISS - to stop missing values being imputed    */
/*                                                                    */
/*====================================================================*/.
 !LET !SCALE       = !1.
 !LET !SCALNAM     = !2.
 !LET !NITEMS      = !3.
 !LET !IRANGE      = !4
 !LET !QLQVARS     = !5.
 COMPUTE  #SMEAN   =  MEAN   ( !QLQVARS ) .
 COMPUTE  #SNUMR   =  NVALID ( !QLQVARS ) .
 !LET !FSCALE = (!INDEX(!6,FSCALE)>0).
 !LET !XMISS = (!INDEX(!6,XMISS)>0).
 IF (#SNUMR   GE  !NITEMS/2)      !SCALE = ((#SMEAN-1.0)/(!IRANGE))*100 .
 IF (!FSCALE  EQ  1)                     !SCALE = 100.0 - !SCALE .
 IF (!XMISS EQ  1  AND  #SNUMR LT !NITEMS)    !SCALE = $SYSMIS .
 VARIABLE LABEL !SCALE  !SCALNAM .
!enddefine .
```

```
/*=====================================================================*/
/*                                                                     */
/* Program for the QLQ-C30 (all versions)                              */
/*                                                                     */
/*=====================================================================*/
.

define  qlqscal ( !POSITIONAL !TOKENS(1)
                 /!POSITIONAL !CMDEND).
!LET !QLQ = !1.
!LET !USEROP = !2.

!IF (!QLQ = 3) !THEN.
MISSING VALUES      q1, q2, q3, q4, q5, q6, q7, q8, q9,
               q10,q11,q12,q13,q14,q15,q16,q17,q18,q19,
               q20,q21,q22,q23,q24,q25,q26,q27,q28 (0,5 thru HIGHEST).
MISSING VALUES q29,q30 (0,8 thru HIGHEST).
qlqsub  QL2 'Global health status/QoL'  2 6 (q29,q30)          !USEROP.
qlqsub  PF2 'Physical Function'         5 3 (q1,q2,q3,q4,q5)  FSCALE !USEROP.
qlqsub  RF2 'Role Function'             2 3 (q6,q7)           FSCALE !USEROP.
qlqsub  EF  'Emotional Function'        4 3 (q21,q22,q23,q24) FSCALE !USEROP.
qlqsub  CF  'Cognitive Function'        2 3 (q20,q25)         FSCALE !USEROP.
qlqsub  SF  'Social Function'           2 3 (q26,q27)         FSCALE !USEROP.
qlqsub  FA  'Fatigue'                   3 3 (q10,q12,q18)     !USEROP.
qlqsub  NV  'Nausea / vomiting'         2 3 (q14,q15)         !USEROP.
qlqsub  PA  'Pain'                      2 3 (q9,q19)          !USEROP.
qlqsub  DY  'Dyspnoea'                  1 3 (q8)              !USEROP.
qlqsub  SL  'Insomnia'                  1 3 (q11)             !USEROP.
qlqsub  AP  'Appetite loss'             1 3 (q13)             !USEROP.
qlqsub  CO  'Constipation'              1 3 (q16)             !USEROP.
qlqsub  DI  'Diarrhoea'                 1 3 (q17)             !USEROP.
qlqsub  FI  'Financial problems'        1 3 (q28).
!IFEND.

!IF (!QLQ = 2) !THEN .
MISSING VALUES q1, q2, q3, q4, q5 (0,3 thru HIGHEST).
MISSING VALUES q6, q7, q8, q9,
               q10,q11,q12,q13,q14,q15,q16,q17,q18,q19,
               q20,q21,q22,q23,q24,q25,q26,q27,q28 (0,5 thru HIGHEST).
MISSING VALUES q29,q30 (0,8 thru HIGHEST).
qlqsub  QL2 'Global health status/QoL'  2 6 (q29,q30)          !USEROP.
qlqsub  PF  'Physical Function'         5 1 (q1,q2,q3,q4,q5)  FSCALE !USEROP.
qlqsub  RF2 'Role Function'             2 3 (q6,q7)           FSCALE !USEROP.
qlqsub  EF  'Emotional Function'        4 3 (q21,q22,q23,q24) FSCALE !USEROP.
qlqsub  CF  'Cognitive Function'        2 3 (q20,q25)         FSCALE !USEROP.
qlqsub  SF  'Social Function'           2 3 (q26,q27)         FSCALE !USEROP.
qlqsub  FA  'Fatigue'                   3 3 (q10,q12,q18)     !USEROP.
qlqsub  NV  'Nausea / vomiting'         2 3 (q14,q15)         !USEROP.
qlqsub  PA  'Pain'                      2 3 (q9,q19)          !USEROP.
qlqsub  DY  'Dyspnoea'                  1 3 (q8)              !USEROP.
qlqsub  SL  'Insomnia'                  1 3 (q11)             !USEROP.
qlqsub  AP  'Appetite loss'             1 3 (q13)             !USEROP.
qlqsub  CO  'Constipation'              1 3 (q16)             !USEROP.
qlqsub  DI  'Diarrhoea'                 1 3 (q17)             !USEROP.
qlqsub  FI  'Financial problems'        1 3 (q28)             !USEROP.
!IFEND.

!IF (!QLQ = 303) !THEN .
```

```
MISSING VALUES q1, q2, q3, q4, q5, q6, q7 (0,3 thru HIGHEST).
MISSING VALUES q8, q9,
               q10,q11,q12,q13,q14,q15,q16,q17,q18,q19,
               q20,q21,q22,q23,q24,q25,q26,q27,q28, q29, q30
               (0,5 thru HIGHEST).
MISSING VALUES q31,q32,q33 (0,8 thru HIGHEST).
qlqsub  QL2 'Global health status/QoL'  2 6 (q32,q33)          !USEROP.
qlqsub  QL  'Global health status/QoL'  2 6 (q31,q33)          !USEROP.
qlqsub  PF  'Physical Function'         5 1 (q1,q2,q3,q4,q5)  FSCALE !USEROP.
qlqsub  RF2 'Role Function'             2 3 (q26,q27)         FSCALE !USEROP.
qlqsub  RF  'Role Function'             2 1 (q6,q7)           FSCALE !USEROP.
qlqsub  EF  'Emotional Function'        4 3 (q21,q22,q23,q24) FSCALE !USEROP.
qlqsub  CF  'Cognitive Function'        2 3 (q20,q25)         FSCALE !USEROP.
qlqsub  SF  'Social Function'           2 3 (q28,q29)         FSCALE !USEROP.
qlqsub  FA  'Fatigue'                   3 3 (q10,q12,q18)      !USEROP.
qlqsub  NV  'Nausea / vomiting'         2 3 (q14,q15)          !USEROP.
qlqsub  PA  'Pain'                      2 3 (q9,q19)           !USEROP.
qlqsub  DY  'Dyspnoea'                  1 3 (q8)               !USEROP.
qlqsub  SL  'Insomnia'                  1 3 (q11)              !USEROP.
qlqsub  AP  'Appetite loss'             1 3 (q13)              !USEROP.
qlqsub  CO  'Constipation'              1 3 (q16)              !USEROP.
qlqsub  DI  'Diarrhoea'                 1 3 (q17)              !USEROP.
qlqsub  FI  'Financial problems'        1 3 (q30)              !USEROP.
!IFEND.

!IF (!QLQ = 1) !THEN .
MISSING VALUES q1, q2, q3, q4, q5, q6, q7 (0,3 thru HIGHEST).
MISSING VALUES q8, q9,
               q10,q11,q12,q13,q14,q15,q16,q17,q18,q19,
               q20,q21,q22,q23,q24,q25,q26,q27,q28 (0,5 thru HIGHEST).
MISSING VALUES q29,q30 (0,8 thru HIGHEST).
qlqsub  QL  'Global health status/QoL'  2 6 (q29,q30)          !USEROP.
qlqsub  PF  'Physical Function'         5 1 (q1,q2,q3,q4,q5)  FSCALE !USEROP.
qlqsub  RF  'Role Function'             2 1 (q6,q7)           FSCALE !USEROP.
qlqsub  EF  'Emotional Function'        4 3 (q21,q22,q23,q24) FSCALE !USEROP.
qlqsub  CF  'Cognitive Function'        2 3 (q20,q25)         FSCALE !USEROP.
qlqsub  SF  'Social Function'           2 3 (q26,q27)         FSCALE !USEROP.
qlqsub  FA  'Fatigue'                   3 3 (q10,q12,q18)      !USEROP.
qlqsub  NV  'Nausea / vomiting'         2 3 (q14,q15)          !USEROP.
qlqsub  PA  'Pain'                      2 3 (q9,q19)           !USEROP.
qlqsub  DY  'Dyspnoea'                  1 3 (q8)               !USEROP.
qlqsub  SL  'Insomnia'                  1 3 (q11)              !USEROP.
qlqsub  AP  'Appetite loss'             1 3 (q13)              !USEROP.
qlqsub  CO  'Constipation'              1 3 (q16)              !USEROP.
qlqsub  DI  'Diarrhoea'                 1 3 (q17)              !USEROP.
qlqsub  FI  'Financial problems'        1 3 (q28)              !USEROP.
!IFEND.
execute.
!enddefine .
```

```
/*=======================================================================*/
/*                                                                       */
/* Program for the Breast cancer module, QLQ-BR23                        */
/*                                                                       */
/*=======================================================================*/
.
define  brscal (!POSITIONAL !CMDEND).
!LET !USEROP = !1.

MISSING VALUES br1, br2, br3, br4, br5, br6, br7, br8, br9,
               br10,br11,br12,br13,br14,br15,br16,br17,br18,br19,
               br20,br21,br22,br23 (0,5 thru HIGHEST).
*Function scales.
qlqsub BRBI  'Body image'          4 3 (br9,br10,br11,br12)    FSCALE
!USEROP.
qlqsub BRSEF 'Sexual functioning'  2 3 (br14,br15)             !USEROP.
if (br15=1 or br15= $SYSMIS ) br16 = $SYSMIS .
qlqsub BRSEE 'Sexual enjoyment'    1 3 (br16)                  !USEROP.
qlqsub BRFU  'Future perspective'  1 3 (br13)                  FSCALE
!USEROP.
*Symptom scales.
qlqsub BRCT  'Systemic therapy'    7 3 (br1,br2,br3,br4,br6,br7,br8) !USEROP.
qlqsub BRRT  'Breast symptoms'     4 3 (br20,br21,br22,br23)   !USEROP.
qlqsub BRSY  'Arm symptoms'        3 3 (br17,br18,br19)        !USEROP.
qlqsub BRHL  'Hair loss'           1 3 (br5)                   !USEROP.
if (br4=1) BRHL=0 .
execute.
!enddefine .

/*=======================================================================*/
/*                                                                       */
/* Program for the Head & Neck cancer module, QLQ-H&N35                  */
/*                                                                       */
/*=======================================================================*/
.

define  hnscal (!POSITIONAL !CMDEND).
!LET !USEROP = !1.
MISSING VALUES hn1, hn2, hn3, hn4, hn5, hn6, hn7, hn8, hn9,
               hn10,hn11,hn12,hn13,hn14,hn15,hn16,hn17,hn18,hn19,
               hn20,hn21,hn22,hn23,hn24,hn25,hn26,hn27,hn28,hn29,
               hn30 (0,5 thru HIGHEST).
MISSING VALUES hn31,hn32,hn33,hn34,hn35 (0,3 thru HIGHEST).
*Function scales.
*  none.
*Symptom scales.
qlqsub HNPA 'HN Pain'             4 3 (hn1,hn2,hn3,hn4)       !USEROP.
qlqsub HNSW 'HN Swallowing'       4 3 (hn5,hn6,hn7,hn8)       !USEROP.
qlqsub HNSE 'HN Senses'           2 3 (hn13,hn14)             !USEROP.
qlqsub HNSP 'HN Speech'           3 3 (hn16,hn23,hn24)        !USEROP.
qlqsub HNSO 'HN Social eating'    4 3 (hn19,hn20,hn21,hn22)   !USEROP.
qlqsub HNSC 'HN Social contact'   5 3 (hn18,hn25,hn26,hn27,hn28) !USEROP.
qlqsub HNSX 'HN Sexuality'        2 3 (hn29,hn30)             !USEROP.
qlqsub HNTE 'HN Teeth'            1 3 (hn9)                   !USEROP.
qlqsub HNOM 'HN Opening mouth'    1 3 (hn10)                  !USEROP.
qlqsub HNDR 'HN Dry mouth'        1 3 (hn11)                  !USEROP.
qlqsub HNSS 'HN Sticky saliva'    1 3 (hn12)                  !USEROP.
qlqsub HNCO 'HN Coughed'          1 3 (hn15)                  !USEROP.
qlqsub HNFI 'HN Felt ill'         1 3 (hn17)                  !USEROP.
qlqsub HNPK 'HN Pain killers'     1 1 (hn31)                  !USEROP.
```

```
qlqsub HNNU 'HN Nutritional supp.' 1 1 (hn32)                  !USEROP.
qlqsub HNFE 'HN Feeding tube'      1 1 (hn33)                  !USEROP.
qlqsub HNWL 'HN Weight loss'       1 1 (hn34)                  !USEROP.
qlqsub HNWG 'HN Weight gain'       1 1 (hn35)                  !USEROP.
execute.
!enddefine .

/*========================================================================*/
/*                                                                        */
/* Program for the Lung cancer module, QLQ-LC13                           */
/*                                                                        */
/*========================================================================*/
.
define  lcscal (!POSITIONAL !CMDEND).
!LET !USEROP = !1.

MISSING VALUES lc1, lc2, lc3, lc4, lc5, lc6, lc7, lc8, lc9,
               lc10,lc11,lc12,lc13 (0,5 thru HIGHEST).
*Function scales.
*  none.
*Symptom scales.
qlqsub LCDY 'LC Dyspnoea'          3 3 (lc3,lc4,lc5) XMISS     !USEROP.
qlqsub LCCO 'LC Coughing'          1 3 (lc1)                   !USEROP.
qlqsub LCHA 'LC Haemoptysis'       1 3 (lc2)                   !USEROP.
qlqsub LCSM 'LC Sore mouth'        1 3 (lc6)                   !USEROP.
qlqsub LCDS 'LC Dysphagia'         1 3 (lc7)                   !USEROP.
qlqsub LCPN 'LC Periph. neuropathy' 1 3 (lc8)                  !USEROP.
qlqsub LCHR 'LC Alopecia'          1 3 (lc9)                   !USEROP.
qlqsub LCPC 'LC Pain in chest'     1 3 (lc10)                  !USEROP.
qlqsub LCPA 'LC Pain in arm'       1 3 (lc11)                  !USEROP.
qlqsub LCPO 'LC Pain other'        1 3 (lc12)                  !USEROP.
execute.
!enddefine.

/*========================================================================*/
/*                                                                        */
/*  For QLQ-C30(V3)  enter   "qlqscal 3. "                                */
/*  For QLQ-C30(V2)  enter   "qlqscal 2. "                                */
/*  For QLQ-C30(V1)  enter   "qlqscal 1. "                                */
/*  For QLQ-C30(+3)  enter   "qlqscal 303. "                              */
/*  For QLQ-BR23     enter   "brscal. "                                   */
/*  For QLQ-H&N35    enter   "hnscal. "                                   */
/*  For QLQ-LC13     enter   "lcscal. "                                   */
/*                                                                        */
/*  Usually, if less than half the scale items are missing, imputation is */
/*   used; the method is described in the Scoring Manual.                 */
/*  To cause missing values to result in the scale score being missing,   */
/*  enter  XMISS  after the command.  For example,  "qlqscal  1 XMISS."   */
/*                                                                        */
/*========================================================================*/
.
```

Appendix 3c: STATA ado files for scoring the QLQ-C30 and supplementary modules

Installation

The .ADO files for STATA should be copied to your ADO directory; usually this will be a directory such as `C:\ADO`. To check for existing ADO directories, enter STATA and type

```
. adopath
```

Details are given in the STATA manuals (e.g. Section U23.5, U23.6 in the Release 5 User's guide)

How to use

The QLQ-C30 variables should be named `q1`, `q2`, `q3`, ... `q30` (or up to `q33` if using the QLQ-C30 (+3)).
If analysing data for the QLQ-BR23, QLQ-H&N35 or QLQ-LC13, their variables should be named br1, `br2`, ...; `hn1`, `hn2`, ...; or `lc1`, `lc2`, ... respectively.

To run the commands, first load your data set into STATA. Typically, type

```
. use <your data set>
```

Then enter one of the following commands as appropriate, according to the version of the QLQ-C30 that you used (V3, V2, V1, or (+3) respectively):

```
. qlqscal 3
. qlqscal 2
. qlqscal 1
. qlqscal 303
```

For the BR23, H&N35 and LC13 modules the corresponding commands are:

```
. brscal
. hnscal
. lcscal
```

What you get

Function scales, symptom scales and single-item scales are calculated and labelled in accordance with the descriptions in the QLQ-C30 Scoring Manual. For example, the emotional functioning scale is calculated from items q21, q22, q23, q24. It is called EF, and is labelled "Emotional functioning". It is scaled to range from 0 to 100, with a high score indicating high emotional functioning.

Note that if you have an existing variable in your data set called EF, it will be automatically over-written. If this is not what you want, you should take care ***not*** to save your revised data set into your original file on disk when you quit from STATA.

Out-of-range values

The ado files automatically check for items with out-of-range values, and will set these to missing. Any changes are notified on the screen. These changes affect the working data set, and it is up to you to decide whether to save this revised data set to disk, possibly overwriting your original disk file. When you quit from STATA, it will ask you whether you wish to save (overwrite) your disk file.

Missing items

The default action of these programs is to impute missing items provided at least half the scale items are present; the method is described in the Scoring Manual. This imputation algorithm accounts for part of the complexity of the code in qlqsub.ado.

You can over-ride this default action. To cause the presence of any missing values in the scale items to result in their scale score being missing, enter `XMISS` after the command. For example,

```
. qlqscal  1 XMISS
```

Writing your own modules

If you wish to adapt these ado files for handling items and scales in new QoL items or scales that you develop, it is very easy.

The files brscal.ado, hnscal.ado and lcscal.ado all correspond directly to the tables given in the QLQ-C30 Scoring Manual, and serve as examples. Thus, all you need to do is prepare a similar table for your own items or scales, and copy the contents of the table into your own file called `myitems.ado`. This can then be edited to use the identical syntax to the examples. Make sure that the header and final "`end`;" lines also follow the example, with the name on the "`program define`" command being identical to the name of your ado file.

That's all – it's simple!

STATA program for scoring the QLQ-C30 and Modules

```
* STATA COMMANDS to generate QLQ scales.
* ===================================.
* .
* STATA release 5.
*
/*=====================================================================*/
/* This code is provided as is without warranty of any kind, either       */
/* expressed or implied.  It is not guaranteed to be error free, and in   */
/* no event will we accept liability for any damages arising out of the   */
/* use of this code.                                                       */
/*=====================================================================*/
* .
/*=====================================================================*/
/*   qlqsub is the utility routine called by the STATA programs that      */
/*         calculate scores for the QLQ-C30 and its modules.              */
/*                                                                         */
/* ARGUMENTS                                                               */
/*  Name of scale                    scale       e.g.  EF                  */
/*  Description of scale             scalnam     e.g.  'Emotional func'   */
/*  Number of items in scale         nitems      e.g.  4                   */
/*  Range of each item in the scale  irange      e.g.  3                   */
/*  List of items in the scale       qlqvars     e.g.  (q21,q22,q23,q24) */
/*  (Optional) string FSCALE - indicates function scales (highscore=good) */
/*  (Optional) string XMISS - to stop missing values being imputed        */
/*                                                                         */
/* Peter Fayers,  30 August, 1998                                          */
/*=====================================================================*/
* .
cap prog drop qlqsub
program define qlqsub
version 5.0
parse "`*'",parse("'()")

local scale="`1'"
capture confirm new v `1'
if _rc!=0 {
    drop `1'
    disp "Replacing existing variable `1'"
}
local i1=index("`1'"," ")+1
if substr("`1'",`i1',1)~="" {disp in red "Syntax error in code: scale
<`scale'>" }

if ("`2'" =="'" & "`4'"=="'" ) { local scalnam="`3'" }
else disp in red "Syntax error in code:  <`2'`3'`4'> in scale `scale'"

if ("`6'" =="(" & "`8'"==")" ) { local varlist="`7'" }
else disp in red "Syntax error in code:  QLQ varlist in scale `scale'"
local scr1="`5'"
local fscale=index(upper("`9'"),"FSCALE")>0
local xmiss=index(upper("`9'"),"XMISS")>0 | index(upper("$userop"),"XMISS")>0
if `xmiss'==1 {
 disp "Scales with missing items are scored as missing (`scale')"}
parse "`scr1'",parse(" ")
confirm integer n `1'
local nitems=`1'
confirm integer n `2'
local irange=`2'
local maxval=`irange'+1

parse "`varlist'",parse(", ")
local nn=0
while "`1'"~="" {
    if "`1'"~=","{
    local nn=`nn'+1
    local qlqvars="`qlqvars'"+" "+"`1'"
    }
macro shift }
if `nn'~=`nitems' {
 disp in red "Syntax error - not `nitems' items in scale `scale'"}
confirm v `qlqvars'

/* =============== end of syntax parsing and checking ========== */

* Check data for out of range values.
local rhs="`qlqvars'"
while "`rhs'"~="" {
       parse "`rhs'",parse(" ")
    local var1="`1'"
    macro shift
    local rhs="`*'"
    quietly summ `var1'
    if _result(5)<1|_result(6)>`maxval' {
               disp "Out-of-range values of `var1': set to missing"
            replace `var1'=. if !( `var1'>0 & `var1'<=`maxval' )
    }
 }

* Calculate scale values
quietly {
tempvar xmean, xnum fscalev
egen `xnum'=robs(`qlqvars')
if `nitems'==1 {gen `xmean'=`qlqvars' }
else            egen `xmean'=rmean(`qlqvars')

gen `fscalev'=((`xmean'-1.0)/`irange')*100. if `xnum'>=`nitems'/2
replace `fscalev' =. if `xmiss'==1 & `xnum'<`nitems'

if `fscale'==1 {replace `fscalev' = 100.0 - `fscalev'}
gen `scale' = `fscalev'
format `scale' %6.2f
label var `scale' "`scalnam'"
}
end
* QLQSUB finished.
```

```
/*=====================================================================*/
/*                                                                     */
/* STATA ado file for the QLQ-C30 (all versions)                       */
/* Calls ado file qlqsub                                               */
/*                                                                     */
/*  For QLQ-C30(V3)  enter   ". qlqscal 3 "                            */
/*  For QLQ-C30(V2)  enter   ". qlqscal 2 "                            */
/* scales and items that are common to all versions                    */
/*  For QLQ-C30(V1)  enter   ". qlqscal 1 "                            */
/*  For QLQ-C30(+3)  enter   ". qlqscal 303 "                          */
/*                                                                     */
/*  Usually, if less than half the scale items are missing, imputation is */
/*  is used; the method is described in the Scoring Manual.           */
/*  To cause missing values to result in the scale score being missing,  */
/*  enter XMISS after the command.  For example,  "qlqscal  1 XMISS"    */
/*                                                                     */
/*  Peter Fayers,  30 August, 1998                                     */
/*=====================================================================*/
```

program define qlqscal

```
version 5.0
parse "`*'",parse(" '()")
#delimit ;
local  qlq = `1';
global  userop = "`2'";

if (`qlq' == 3) {
qlqsub  QL2 'Global health status/QoL'  2 6 (q29,q30)          ;
qlqsub  PF2 'Physical Function'         5 3 (q1,q2,q3,q4,q5)  FSCALE ;
qlqsub  RF2 'Role Function'             2 3 (q6,q7)            FSCALE ;
qlqsub  EF 'Emotional Function'         4 3 (q21,q22,q23,q24) FSCALE ;
qlqsub  CF 'Cognitive Function'         2 3 (q20,q25)          FSCALE ;
qlqsub  SF 'Social Function'            2 3 (q26,q27)          FSCALE ;
qlqsub  FA 'Fatigue'                    3 3 (q10,q12,q18)      ;
qlqsub  NV 'Nausea / vomiting'          2 3 (q14,q15)          ;
qlqsub  PA 'Pain'                       2 3 (q9,q19)           ;
qlqsub  DY 'Dyspnoea'                   1 3 (q8)               ;
qlqsub  SL 'Insomnia'                   1 3 (q11)              ;
qlqsub  AP 'Appetite loss'              1 3 (q13)              ;
qlqsub  CO 'Constipation'               1 3 (q16)              ;
qlqsub  DI 'Diarrhoea'                  1 3 (q17)              ;
qlqsub  FI 'Financial problems'         1 3 (q28)              ;
};


if (`qlq' == 2) {
qlqsub  QL2 'Global health status/QoL'  2 6 (q29,q30)          ;
qlqsub  PF  'Physical Function'         5 1 (q1,q2,q3,q4,q5)  FSCALE ;
qlqsub  RF2 'Role Function'             2 3 (q6,q7)            FSCALE ;
qlqsub  EF 'Emotional Function'         4 3 (q21,q22,q23,q24) FSCALE ;
qlqsub  CF 'Cognitive Function'         2 3 (q20,q25)          FSCALE ;
qlqsub  SF 'Social Function'            2 3 (q26,q27)          FSCALE ;
qlqsub  FA 'Fatigue'                    3 3 (q10,q12,q18)      ;
qlqsub  NV 'Nausea / vomiting'          2 3 (q14,q15)          ;
qlqsub  PA 'Pain'                       2 3 (q9,q19)           ;
qlqsub  DY 'Dyspnoea'                   1 3 (q8)               ;
qlqsub  SL 'Insomnia'                   1 3 (q11)              ;
qlqsub  AP 'Appetite loss'              1 3 (q13)              ;
qlqsub  CO 'Constipation'               1 3 (q16)              ;
qlqsub  DI 'Diarrhoea'                  1 3 (q17)              ;
qlqsub  FI 'Financial problems'         1 3 (q28)              ;
};

if (`qlq' == 303) {
qlqsub  QL2 'Global health status/QoL'  2 6 (q32,q33)          ;
qlqsub  QL  'Global health status/QoL'  2 6 (q31,q33)          ;
qlqsub  PF  'Physical Function'         5 1 (q1,q2,q3,q4,q5)  FSCALE ;
qlqsub  RF2 'Role Function'             2 3 (q26,q27)          FSCALE ;
qlqsub  RF  'Role Function'             2 1 (q6,q7)            FSCALE ;
qlqsub  EF 'Emotional Function'         4 3 (q21,q22,q23,q24) FSCALE ;
qlqsub  CF 'Cognitive Function'         2 3 (q20,q25)          FSCALE ;
qlqsub  SF  'Social Function'           2 3 (q28,q29)          FSCALE ;
qlqsub  FA 'Fatigue'                    3 3 (q10,q12,q18)      ;
qlqsub  NV 'Nausea / vomiting'          2 3 (q14,q15)          ;
qlqsub  PA 'Pain'                       2 3 (q9,q19)           ;
qlqsub  DY 'Dyspnoea'                   1 3 (q8)               ;
qlqsub  SL 'Insomnia'                   1 3 (q11)              ;
qlqsub  AP 'Appetite loss'              1 3 (q13)              ;
qlqsub  CO 'Constipation'               1 3 (q16)              ;
qlqsub  DI 'Diarrhoea'                  1 3 (q17)              ;
qlqsub FI  'Financial problems'         1 3 (q30)              ;
};

if (`qlq' == 1) {
qlqsub  QL  'Global health status/QoL'  2 6 (q29,q30)          ;
qlqsub  PF  'Physical Function'         5 1 (q1,q2,q3,q4,q5)  FSCALE ;
qlqsub  RF  'Role Function'             2 1 (q6,q7)            FSCALE ;
qlqsub  EF 'Emotional Function'         4 3 (q21,q22,q23,q24) FSCALE ;
qlqsub  CF 'Cognitive Function'         2 3 (q20,q25)          FSCALE ;
qlqsub  SF  'Social Function'           2 3 (q26,q27)          FSCALE ;
qlqsub  FA 'Fatigue'                    3 3 (q10,q12,q18)      ;
qlqsub  NV 'Nausea / vomiting'          2 3 (q14,q15)          ;
qlqsub  PA 'Pain'                       2 3 (q9,q19)           ;
qlqsub  DY 'Dyspnoea'                   1 3 (q8)               ;
qlqsub  SL 'Insomnia'                   1 3 (q11)              ;
qlqsub  AP 'Appetite loss'              1 3 (q13)              ;
qlqsub  CO 'Constipation'               1 3 (q16)              ;
qlqsub  DI 'Diarrhoea'                  1 3 (q17)              ;
qlqsub  FI  'Financial problems'        1 3 (q28)              ;
};
end;
```

```
/*=======================================================================*/
/*                                                                       */
/* STATA ado file for the Breast cancer module, QLQ-BR23                 */
/* Calls ado file qlqsub                                                 */
/*                                                                       */
/*  For QLQ-BR23     enter   ". brscal "                                 */
/*                                                                       */
/*  Usually, if less than half the scale items are missing, imputation is */
/*  is used; the method is described in the Scoring Manual.              */
/*  To cause missing values to result in the scale score being missing,  */
/*  enter XMISS after the command.  For example,  "brscal  XMISS"        */
/*                                                                       */
/*  Peter Fayers,  30 August, 1998                                       */
/*=======================================================================*/
```

program define brscal

```
version 5.0
parse "`*'",parse(" '()")
#delimit ;
global  userop = "`1'";

*Function scales;
qlqsub BRBI  'Body image'           4 3 (br9,br10,br11,br12)          FSCALE ;
qlqsub BRSEF 'Sexual functioning'  2 3 (br14,br15)                    ;
quietly replace br16=. if br15==1 | br15==.                           ;
qlqsub BRSEE 'Sexual enjoyment'    1 3 (br16)                         ;
qlqsub BRFU  'Future perspective'  1 3 (br13)                         FSCALE ;
*Symptom scales;
qlqsub BRCT  'Systemic therapy'    7 3 (br1,br2,br3,br4,br6,br7,br8) ;
qlqsub BRRT  'Breast symptoms'     4 3 (br20,br21,br22,br23)          ;
qlqsub BRSY  'Arm symptoms'        3 3 (br17,br18,br19)               ;
qlqsub BRHL  'Hair loss'           1 3 (br5)                          ;
quietly replace BRHL=0 if br4==1                                      ;
end ;
```

```
/*=======================================================================*/
/*                                                                       */
/* STATA ado file for the Head & Neck cancer module, QLQ-H&N35         */
/* Calls ado file qlqsub                                                 */
/*                                                                       */
/*  For QLQ-H&N35    enter   ". hnscal "                                 */
/*                                                                       */
/*  Usually, if less than half the scale items are missing, imputation is */
/*  is used; the method is described in the Scoring Manual.              */
/*  To cause missing values to result in the scale score being missing,  */
/*  enter XMISS after the command.  For example,  "hnscal  XMISS"        */
/*                                                                       */
/*  Peter Fayers,  30 August, 1998                                       */
/*=======================================================================*/
```

program define hnscal

```
version 5.0
parse "`*'",parse(" '()")
#delimit ;
global  userop = "`1'";

*Function scales;
*  none;
*Symptom scales;
qlqsub HNPA 'HN Pain'              4 3 (hn1,hn2,hn3,hn4)              ;
qlqsub HNSW 'HN Swallowing'        4 3 (hn5,hn6,hn7,hn8)              ;
qlqsub HNSE 'HN Senses'            2 3 (hn13,hn14)                    ;
qlqsub HNSP 'HN Speech'            3 3 (hn16,hn23,hn24)               ;
qlqsub HNSO 'HN Social eating'     4 3 (hn19,hn20,hn21,hn22)          ;
qlqsub HNSC 'HN Social contact'    5 3 (hn18,hn25,hn26,hn27,hn28) ;
qlqsub HNSX 'HN Sexuality'         2 3 (hn29,hn30)                    ;
qlqsub HNTE 'HN Teeth'             1 3 (hn9)                          ;
qlqsub HNOM 'HN Opening mouth'     1 3 (hn10)                         ;
qlqsub HNDR 'HN Dry mouth'         1 3 (hn11)                         ;
qlqsub HNSS 'HN Sticky saliva'     1 3 (hn12)                         ;
qlqsub HNCO 'HN Coughed'           1 3 (hn15)                         ;
qlqsub HNFI 'HN Felt ill'          1 3 (hn17)                         ;
qlqsub HNPK 'HN Pain killers'      1 1 (hn31)                         ;
qlqsub HNNU 'HN Nutritional supp'  1 1 (hn32)                         ;
qlqsub HNFE 'HN Feeding tube'      1 1 (hn33)                         ;
qlqsub HNWL 'HN Weight loss'       1 1 (hn34)                         ;
qlqsub HNWG 'HN Weight gain'       1 1 (hn35)                         ;
end;
```

```
/*======================================================================*/
/*                                                                      */
/* STATA ado file for the Lung cancer module, QLQ-LC13                  */
/* Calls ado file qlqsub                                                */
/*                                                                      */
/*  For QLQ-LC13     enter   ". lcscal "                                */
/*                                                                      */
/*  Usually, if less than half the scale items are missing, imputation is */
/*  is used; the method is described in the Scoring Manual.             */
/*  To cause missing values to result in the scale score being missing,  */
/*  enter XMISS after the command.  For example,  "lcscal  XMISS"       */
/*                                                                      */
/*  Peter Fayers,  30 August, 1998                                      */
/*======================================================================*/
```

program define lcscal

```
version 5.0
parse "`*'",parse(" '()")
#delimit ;
global  userop = "`1'";

*Function scales;
*  none;
*Symptom scales;
qlqsub LCDY 'LC Dyspnoea'          3 3 (lc3,lc4,lc5) XMISS     ;
qlqsub LCCO 'LC Coughing'          1 3 (lc1)                   ;
qlqsub LCHA 'LC Haemoptysis'       1 3 (lc2)                   ;
qlqsub LCSM 'LC Sore mouth'        1 3 (lc6)                   ;
qlqsub LCDS 'LC Dysphagia'         1 3 (lc7)                   ;
qlqsub LCPN 'LC Periph neuropathy' 1 3 (lc8)                   ;
qlqsub LCHR 'LC Alopecia'          1 3 (lc9)                   ;
qlqsub LCPC 'LC Pain in chest'     1 3 (lc10)                  ;
qlqsub LCPA 'LC Pain in arm'       1 3 (lc11)                  ;
qlqsub LCPO 'LC Pain other'        1 3 (lc12)                  ;
end;
```

```
* ======================================================== .
* STATA COMMANDS to label QLQ scales                        .
*                                                           .
* QLQLABL.ado                                               .
*                                                           .
* It is assumed that the variables are  q1, q2, q3 etc      .
*                                                           .
* Call with argument equal to QLQ version number -          .
*  i.e.  1, 303, 2, or 3.                                   .
*  E.g.                                                     .
*     qlqlabl  303                                          .
*                                                           .
*                                                           .
* Peter Fayers,  30 August, 1998                            .
* ======================================================== .

/*  Install as QLQLABL.ado   */


capture program drop qlqlabl
```

program define qlqlabl

```
version 5.0
parse "`*'",parse(" ")
cap local qlq=`1'

capture confirm integer number `1'
if _rc~=0 {
   disp in red "QLQ version number is missing"
   exit
}

label variable q1   "Carrying"
label variable q2   "Long_walk"
label variable q3   "Short_walk"
label variable q4   "In_bed"
label variable q5   "Need_help"

if `qlq'==1 | `qlq'==303 {
label variable q6   "Limited_jobs"
label variable q7   "Unable_to_work"
}

if `qlq'==2 | `qlq'==3 {
label variable q6   "Limited_activity"
label variable q7   "Limited_hobbies"
}

label variable q8   "SoB"
label variable q9   "Pain"
label variable q10  "Need_rest"
label variable q11  "Insomnia"
label variable q12  "Weakness"
label variable q13  "Appetite"
label variable q14  "Nausea"
label variable q15  "Vomiting"
label variable q16  "Constipation"
label variable q17  "Diarrhoea"
label variable q18  "Tiredness"
label variable q19  "Pain_interference"
```

```
label variable q20  "Lack_concentration"
label variable q21  "Tense"
label variable q22  "Worried"
label variable q23  "Irritable"
label variable q24  "Depressed"
label variable q25  "Memory"

if `qlq'==1 {
label variable q26  "Family_life"
label variable q27  "Social_activities"
label variable q28  "Financial_problems"
label variable q29  "Physical_cond"
label variable q30  "Global_QoL"
}

if `qlq'==303 {
label variable q26  "Limited_activity"
label variable q27  "Limited_hobbies"
label variable q28  "Financial_problems"
label variable q29  "Social_activities"
label variable q30  "Financial_problems"
label variable q31  "Physical_cond"
label variable q32  "Overall_health"
label variable q33  "Global_QoL"
}

if `qlq'==2 | `qlq'==3 {
label variable q26  "Family_life"
label variable q27  "Social_activities"
label variable q28  "Financial_problems"
label variable q29  "Overall_health"
label variable q30  "Global_QoL"
}
```

```
cap label drop q2labl
cap label drop q4labl
cap label drop q7labl
label define q2labl 1 "No" 2 "Yes"
label define q4labl 1 "Not at all" 2 "A little" 3 "Quite bit" 4 "V. much"
label define q7labl 1 "V. poor" 7 "Excellent"

quietly{
if `qlq'== 1 {
  for 1-7, ltype(n):label values q@ q2labl
  for 8-28, ltype(n):label values q@ q4labl
  for 29-30, ltype(n):label values q@ q7labl
}

if `qlq'== 303 {
  for 1-7, ltype(n):label values q@ q2labl
  for 8-30, ltype(n):label values q@ q4labl
  for 31-33, ltype(n):label values q@ q7labl
}
if `qlq'== 2 {
  for 1-5, ltype(n):label values q@ q2labl
  for 6-28, ltype(n):label values q@ q4labl
  for 29-30, ltype(n):label values q@ q7labl
}
if `qlq'== 3 {
  for 1-28, ltype(n):label values q@ q4labl
  for 29-30, ltype(n):label values q@ q7labl
}
}
end
```

This manual is one of a series:

EORTC QLQ-C30 Reference Values. Fayers PM, Weeden S, Curran D, on behalf of the EORTC Quality of Life Study Group. Brussels: EORTC, 1998. ISBN: 2-930064-11-0.
(The reference values are also available on CD-ROM)

EORTC Quality of Life Study Group Guidelines for Developing Questionnaire Modules. Sprangers M, Cull A, Groenvold M, on behalf of the EORTC Quality of Life Study Group. Brussels: EORTC, 1998. ISBN: 2-930064-10-2.

EORTC Quality of Life Study Group Translation Procedure. Cull A, Sprangers M, Bjordal K, Aaronson N, on behalf of the EORTC Quality of Life Study Group. Brussels: EORTC, 1998. ISBN: 2-930064-15-3.

Guidelines for Assessing Quality of Life in EORTC Clinical Trials. Young T, de Haes H, Curran D, Fayers P, Brandberg Y, on behalf of the EORTC Quality of Life Study Group. Brussels: EORTC, 1999. ISBN: 2-930064-17-X.

EORTC QLQ-C30 Scoring Manual (3rd edition). Fayers PM, Aaronson NK, Bjordal K, Groenvold M, Curran D, Bottomley A, on behalf of the EORTC Quality of Life Group. Brussels: EORTC, 2001. ISBN: 2-9300 64-22-6.

Contents

THE EORTC QLQ-C30 1

Introduction 1

Background 2

The EORTC 2

EORTC QLQ-C36 2

EORTC QLQ-C30 version 1.0 2

EORTC QLQ-C30 (+3) 3

EORTC QLQ-C30 version 2.0 3

EORTC QLQ-C30 version 3.0 3

Citation and Availability 4

SCORING PROCEDURES 5

General principles of scoring 6

Scoring the EORTC QLQ-C30 version 3.0 7

Scoring earlier versions of the EORTC QLQ-C30 9

Missing data 10

Missing items 10

Missing forms 11

Theory of scaling 13

Interpretation of scores 14

Use of statistical packages 15

QLQ SUPPLEMENTARY MODULES 16

Breast cancer module: QLQ-BR23 18

Head & Neck cancer module: QLQ-H&N35 19

Lung cancer module: QLQ-LC13 20

Oesophageal cancer module: QLQ-OES24 21

Ovarian cancer module: QLQ-OV28 22

QLQ modules under development - phase III 23

- Bladder cancer modules: QLQ-BLsup24, QLQ-BLmi30 23
- Brain cancer module: QLQ-BN20 23
- Colorectal cancer module: QLQ-CR38 23
- Gastric cancer module: QLQ-STO22 23
- Multiple myeloma module: QLQ-MY24 24
- Ophthalmic cancer module: QLQ-OPT37 24
- Pancreatic cancer module: QLQ-PAN26 24
- Prostate cancer module: QLQ-PR25 24
- Satisfaction with care module: QLQ-SAT32 24

QLQ modules under development - phases I and II **25**
- Carcinoid module 25
- Choices and decision-making module 25
- Fatigue module 25
- High-dose chemotherapy module 25
- Information module 25
- Liver module 25
- Peripheral neuropathy module 25
- Symptom check list 25

TRANSLATIONS 27

REFERENCES 30

GLOSSARY 34

APPENDIX 1 QLQ-C30 35

Appendix 1a: QLQ-C30 version 1.0 **36**

Appendix 1c: QLQ-C30 version 2.0 **39**

Appendix 1d: QLQ-C30 version 3.0 **41**

APPENDIX 2 QLQ MODULES 43

Appendix 2a: Breast cancer module QLQ-BR23 **44**

Appendix 2b: Head & Neck cancer module QLQ-H&N35 **46**

Appendix 2c: Lung cancer module QLQ-LC13 **48**

Appendix 2d: Oesophageal cancer module QLQ-OES24 **49**

Appendix 2e: Ovarian cancer module QLQ-OV28 **51**

APPENDIX 3 CODING FOR STATISTICAL PACKAGES 53

Appendix 3a: SAS program for scoring the QLQ-C30 and supplementary modules **54**

Appendix 3b: SPSS program for scoring the QLQ-C30 and supplementary modules **61**

Appendix 3c: STATA ado files for scoring the QLQ-C30 and supplementary modules **67**
