# Supplementary material for: Financial toxicity on treatment outcomes in head & neck cancer patients undergoing radiation therapy
Source: Radiat Oncol. 2025 Nov 25;20:176. doi: 10.1186/s13014-025-02749-x (PMC12648789; doi:10.1186/s13014-025-02749-x)
Supplement: Supplementary file 3 — Supplementary material 3 [file 13014_2025_2749_MOESM3_ESM.pdf]

NCI- PRO-CTCAE® ITEMS-ENGLISH (UNIVERSAL)

Item Library Version 1.0

As individuals go through treatment for their cancer they sometimes experience different symptoms and side effects. For each question, please select the one response that best describes your experiences over the past 7 days…

**1. PRO-CTCAE® Symptom Term:** Dry mouth				
a. In the last 7 days, what was the SEVERITY of your DRY MOUTH at its WORST?				
O None	O Mild	O Moderate	O Severe	O Very severe

**2. PRO-CTCAE® Symptom Term:** Difficulty swallowing				
a. In the last 7 days, what was the SEVERITY of your DIFFICULTY SWALLOWING at its WORST?				
O None	O Mild	O Moderate	O Severe	O Very severe

**3. PRO-CTCAE® Symptom Term:** Mouth/throat sores				
a. In the last 7 days, what was the SEVERITY of your MOUTH OR THROAT SORES at their WORST?				
O None	O Mild	O Moderate	O Severe	O Very severe
b. In the last 7 days, how much did MOUTH OR THROAT SORES INTERFERE with your usual or daily activities?				
O Not at all	O A little bit	O Somewhat	O Quite a bit	O Very much

**4. PRO-CTCAE® Symptom Term:** Cracking at the corners of the mouth (cheilosis/cheilitis)				
a. In the last 7 days, what was the SEVERITY of SKIN CRACKING AT THE CORNERS OF YOUR MOUTH at its WORST?				
O None	O Mild	O Moderate	O Severe	O Very severe

**5. PRO-CTCAE® Symptom Term:** Voice quality changes	
a. In the last 7 days, did you have any VOICE CHANGES?	
O Yes	O No

The PRO-CTCAE® items and information herein were developed by the Division of Cancer Control and Population Sciences in the NATIONAL CANCER INSTITUTE at the NATIONAL INSTITUTES OF HEALTH, in Bethesda, Maryland, U.S.A. Use of the PRO-CTCAE® is subject to NCI's Terms of Use.

Version date: 5/11/2023

**6. PRO-CTCAE® Symptom Term:** Hoarseness				
a. In the last 7 days, what was the SEVERITY of your HOARSE VOICE at its WORST?				
O None	O Mild	O Moderate	O Severe	O Very severe

**7. PRO-CTCAE® Symptom Term:** Taste changes				
a. In the last 7 days, what was the SEVERITY of your PROBLEMS WITH TASTING FOOD OR DRINK at their WORST?				
O None	O Mild	O Moderate	O Severe	O Very severe

**8. PRO-CTCAE® Symptom Term:** Decreased appetite				
a. In the last 7 days, what was the SEVERITY of your DECREASED APPETITE at its WORST?				
O None	O Mild	O Moderate	O Severe	O Very severe
b. In the last 7 days, how much did DECREASED APPETITE INTERFERE with your usual or daily activities?				
O Not at all	O A little bit	O Somewhat	O Quite a bit	O Very much

**9. PRO-CTCAE® Symptom Term:** Nausea				
a. In the last 7 days, how OFTEN did you have NAUSEA?				
O Never	O Rarely	O Occasionally	O Frequently	O Almost constantly
b. In the last 7 days, what was the SEVERITY of your NAUSEA at its WORST?				
O None	O Mild	O Moderate	O Severe	O Very severe

**10. PRO-CTCAE® Symptom Term:** Vomiting				
a. In the last 7 days, how OFTEN did you have VOMITING?				
O Never	O Rarely	O Occasionally	O Frequently	O Almost constantly
b. In the last 7 days, what was the SEVERITY of your VOMITING at its WORST?				
O None	O Mild	O Moderate	O Severe	O Very severe

The PRO-CTCAE® items and information herein were developed by the Division of Cancer Control and Population Sciences in the NATIONAL CANCER INSTITUTE at the NATIONAL INSTITUTES OF HEALTH, in Bethesda, Maryland, U.S.A. Use of the PRO-CTCAE® is subject to NCI's Terms of Use.

**11. PRO-CTCAE® Symptom Term:** Heartburn				
a. In the last 7 days, how OFTEN did you have HEARTBURN?				
O Never	O Rarely	O Occasionally	O Frequently	O Almost constantly
b. In the last 7 days, what was the SEVERITY of your HEARTBURN at its WORST?				
O None	O Mild	O Moderate	O Severe	O Very severe

**12. PRO-CTCAE® Symptom Term:** Gas	
a. In the last 7 days, did you have any INCREASED PASSING OF GAS (FLATULENCE)?	
O Yes	O No

**13. PRO-CTCAE® Symptom Term:** Bloating				
a. In the last 7 days, how OFTEN did you have BLOATING OF THE ABDOMEN (BELLY)?				
O Never	O Rarely	O Occasionally	O Frequently	O Almost constantly
b. In the last 7 days, what was the SEVERITY of your BLOATING OF THE ABDOMEN (BELLY) at its WORST?				
O None	O Mild	O Moderate	O Severe	O Very severe

**14. PRO-CTCAE® Symptom Term:** Hiccups				
a. In the last 7 days, how OFTEN did you have HICCUPS?				
O Never	O Rarely	O Occasionally	O Frequently	O Almost constantly
b. In the last 7 days, what was the SEVERITY of your HICCUPS at their WORST?				
O None	O Mild	O Moderate	O Severe	O Very severe

**15. PRO-CTCAE® Symptom Term:** Constipation				
a. In the last 7 days, what was the SEVERITY of your CONSTIPATION at its WORST?				
O None	O Mild	O Moderate	O Severe	O Very severe

**16. PRO-CTCAE® Symptom Term:** Diarrhea				
a. In the last 7 days, how OFTEN did you have LOOSE OR WATERY STOOLS (DIARRHEA/DIARRHOEA)?				
O Never	O Rarely	O Occasionally	O Frequently	O Almost constantly

**17. PRO-CTCAE® Symptom Term:** Abdominal pain				
a. In the last 7 days, how OFTEN did you have PAIN IN THE ABDOMEN (BELLY AREA)?				
O Never	O Rarely	O Occasionally	O Frequently	O Almost constantly
b. In the last 7 days, what was the SEVERITY of your PAIN IN THE ABDOMEN (BELLY AREA) at its WORST?				
O None	O Mild	O Moderate	O Severe	O Very severe
c. In the last 7 days, how much did PAIN IN THE ABDOMEN (BELLY AREA) INTERFERE with your usual or daily activities?				
O Not at all	O A little bit	O Somewhat	O Quite a bit	O Very much

**18. PRO-CTCAE® Symptom Term:** Fecal incontinence				
a. In the last 7 days, how OFTEN did you LOSE CONTROL OF BOWEL MOVEMENTS?				
O Never	O Rarely	O Occasionally	O Frequently	O Almost constantly
b. In the last 7 days, how much did LOSS OF CONTROL OF BOWEL MOVEMENTS INTERFERE with your usual or daily activities?				
O Not at all	O A little bit	O Somewhat	O Quite a bit	O Very much

**19. PRO-CTCAE® Symptom Term:** Shortness of breath				
a. In the last 7 days, what was the SEVERITY of your SHORTNESS OF BREATH at its WORST?				
O None	O Mild	O Moderate	O Severe	O Very severe
b. In the last 7 days, how much did your SHORTNESS OF BREATH INTERFERE with your usual or daily activities?				
O Not at all	O A little bit	O Somewhat	O Quite a bit	O Very much

The PRO-CTCAE® items and information herein were developed by the Division of Cancer Control and Population Sciences in the NATIONAL CANCER INSTITUTE at the NATIONAL INSTITUTES OF HEALTH, in Bethesda, Maryland, U.S.A. Use of the PRO-CTCAE® is subject to NCI's Terms of Use.

**20. PRO-CTCAE® Symptom Term:** Cough				
a. In the last 7 days, what was the SEVERITY of your COUGH at its WORST?				
O None	O Mild	O Moderate	O Severe	O Very severe
b. In the last 7 days, how much did COUGH INTERFERE with your usual or daily activities?				
O Not at all	O A little bit	O Somewhat	O Quite a bit	O Very much

**21. PRO-CTCAE® Symptom Term:** Wheezing				
a. In the last 7 days, what was the SEVERITY of your WHEEZING (WHISTLING NOISE IN THE CHEST WITH BREATHING) at its WORST?				
O None	O Mild	O Moderate	O Severe	O Very severe

**22. PRO-CTCAE® Symptom Term:** Swelling				
a. In the last 7 days, how OFTEN did you have ARM OR LEG SWELLING?				
O Never	O Rarely	O Occasionally	O Frequently	O Almost constantly
b. In the last 7 days, what was the SEVERITY of your ARM OR LEG SWELLING at its WORST?				
O None	O Mild	O Moderate	O Severe	O Very severe
c. In the last 7 days, how much did ARM OR LEG SWELLING INTERFERE with your usual or daily activities?				
O Not at all	O A little bit	O Somewhat	O Quite a bit	O Very much

**23. PRO-CTCAE® Symptom Term:** Heart palpitations				
a. In the last 7 days, how OFTEN did you feel a POUNDING OR RACING HEARTBEAT (PALPITATIONS)?				
O Never	O Rarely	O Occasionally	O Frequently	O Almost constantly
b. In the last 7 days, what was the SEVERITY of your POUNDING OR RACING HEARTBEAT (PALPITATIONS) at its WORST?				
O None	O Mild	O Moderate	O Severe	O Very severe

The PRO-CTCAE® items and information herein were developed by the Division of Cancer Control and Population Sciences in the NATIONAL CANCER INSTITUTE at the NATIONAL INSTITUTES OF HEALTH, in Bethesda, Maryland, U.S.A. Use of the PRO-CTCAE® is subject to NCI's Terms of Use.

24. **PRO-CTCAE® Symptom Term:** Rash	
a. In the last 7 days, did you have any RASH?	
O Yes	O No

25. **PRO-CTCAE® Symptom Term:** Skin dryness				
a. In the last 7 days, what was the SEVERITY of your DRY SKIN at its WORST?				
O None	O Mild	O Moderate	O Severe	O Very severe

26. **PRO-CTCAE® Symptom Term:** Acne				
a. In the last 7 days, what was the SEVERITY of your ACNE OR PIMPLES ON THE FACE OR CHEST at its WORST?				
O None	O Mild	O Moderate	O Severe	O Very severe

27. **PRO-CTCAE® Symptom Term:** Hair loss				
a. In the last 7 days, did you have any HAIR LOSS?				
O Not at all	O A little bit	O Somewhat	O Quite a bit	O Very much

28. **PRO-CTCAE® Symptom Term:** Itching				
a. In the last 7 days, what was the SEVERITY of your ITCHY SKIN at its WORST?				
O None	O Mild	O Moderate	O Severe	O Very severe

29. **PRO-CTCAE® Symptom Term:** Hives	
a. In the last 7 days, did you have any HIVES (ITCHY RED BUMPS ON THE SKIN)?	
O Yes	O No

The PRO-CTCAE® items and information herein were developed by the Division of Cancer Control and Population Sciences in the NATIONAL CANCER INSTITUTE at the NATIONAL INSTITUTES OF HEALTH, in Bethesda, Maryland, U.S.A. Use of the PRO-CTCAE® is subject to NCI's Terms of Use.

**30. PRO-CTCAE® Symptom Term:** Hand-foot syndrome				
a. In the last 7 days, what was the SEVERITY of your HAND-FOOT SYNDROME (A RASH OF THE HANDS OR FEET THAT CAN CAUSE CRACKING, PEELING, REDNESS OR PAIN) at its WORST?				
O None	O Mild	O Moderate	O Severe	O Very severe

**31. PRO-CTCAE® Symptom Term:** Nail loss	
a. In the last 7 days, did you LOSE ANY FINGERNAILS OR TOENAILS?	
O Yes	O No

**32. PRO-CTCAE® Symptom Term:** Nail ridging	
a. In the last 7 days, did you have any RIDGES OR BUMPS ON YOUR FINGERNAILS OR TOENAILS?	
O Yes	O No

**33. PRO-CTCAE® Symptom Term:** Nail discoloration	
a. In the last 7 days, did you have any CHANGE IN THE COLOR OF YOUR FINGERNAILS OR TOENAILS?	
O Yes	O No

**34. PRO-CTCAE® Symptom Term:** Sensitivity to sunlight	
a. In the last 7 days, did you have any INCREASED SKIN SENSITIVITY TO SUNLIGHT?	
O Yes	O No

**35. PRO-CTCAE® Symptom Term:** Bed/pressure sores	
a. In the last 7 days, did you have any BED SORES?	
O Yes	O No

The PRO-CTCAE® items and information herein were developed by the Division of Cancer Control and Population Sciences in the NATIONAL CANCER INSTITUTE at the NATIONAL INSTITUTES OF HEALTH, in Bethesda, Maryland, U.S.A. Use of the PRO-CTCAE® is subject to NCI's Terms of Use.

**36. PRO-CTCAE® Symptom Term:** Radiation skin reaction					
a. In the last 7 days, what was the SEVERITY of your SKIN BURNS FROM RADIATION at their WORST?					
O None	O Mild	O Moderate	O Severe	O Very severe	O Not applicable

**37. PRO-CTCAE® Symptom Term:** Skin darkening	
a. In the last 7 days, did you have any UNUSUAL DARKENING OF THE SKIN?	
O Yes	O No

**38. PRO-CTCAE® Symptom Term:** Stretch marks	
a. In the last 7 days, did you have any STRETCH MARKS?	
O Yes	O No

**39. PRO-CTCAE® Symptom Term:** Numbness & tingling				
a. In the last 7 days, what was the SEVERITY of your NUMBNESS OR TINGLING IN YOUR HANDS OR FEET at its WORST?				
O None	O Mild	O Moderate	O Severe	O Very severe
b. In the last 7 days, how much did NUMBNESS OR TINGLING IN YOUR HANDS OR FEET INTERFERE with your usual or daily activities?				
O Not at all	O A little bit	O Somewhat	O Quite a bit	O Very much

**40. PRO-CTCAE® Symptom Term:** Dizziness				
a. In the last 7 days, what was the SEVERITY of your DIZZINESS at its WORST?				
O None	O Mild	O Moderate	O Severe	O Very severe
b. In the last 7 days, how much did DIZZINESS INTERFERE with your usual or daily activities?				
O Not at all	O A little bit	O Somewhat	O Quite a bit	O Very much

The PRO-CTCAE® items and information herein were developed by the Division of Cancer Control and Population Sciences in the NATIONAL CANCER INSTITUTE at the NATIONAL INSTITUTES OF HEALTH, in Bethesda, Maryland, U.S.A. Use of the PRO-CTCAE® is subject to NCI's Terms of Use.

**41. PRO-CTCAE® Symptom Term:** Blurred vision				
a. In the last 7 days, what was the SEVERITY of your BLURRY VISION at its WORST?				
O None	O Mild	O Moderate	O Severe	O Very severe
b. In the last 7 days, how much did BLURRY VISION INTERFERE with your usual or daily activities?				
O Not at all	O A little bit	O Somewhat	O Quite a bit	O Very much

**42. PRO-CTCAE® Symptom Term:** Flashing lights	
a. In the last 7 days, did you have any FLASHING LIGHTS IN FRONT OF YOUR EYES?	
O Yes	O No

**43. PRO-CTCAE® Symptom Term:** Visual floaters	
a. In the last 7 days, did you have any SPOTS OR LINES (FLOATERS) THAT DRIFT IN FRONT OF YOUR EYES?	
O Yes	O No

**44. PRO-CTCAE® Symptom Term:** Watery eyes				
a. In the last 7 days, what was the SEVERITY of your WATERY EYES (TEARING) at their WORST?				
O None	O Mild	O Moderate	O Severe	O Very severe
b. In the last 7 days, how much did WATERY EYES (TEARING) INTERFERE with your usual or daily activities?				
O Not at all	O A little bit	O Somewhat	O Quite a bit	O Very much

**45. PRO-CTCAE® Symptom Term:** Ringing in ears				
a. In the last 7 days, what was the SEVERITY of RINGING IN YOUR EARS at its WORST?				
O None	O Mild	O Moderate	O Severe	O Very severe

The PRO-CTCAE® items and information herein were developed by the Division of Cancer Control and Population Sciences in the NATIONAL CANCER INSTITUTE at the NATIONAL INSTITUTES OF HEALTH, in Bethesda, Maryland, U.S.A. Use of the PRO-CTCAE® is subject to NCI's Terms of Use.

**46. PRO-CTCAE® Symptom Term:** Concentration				
a. In the last 7 days, what was the SEVERITY of your PROBLEMS WITH CONCENTRATION at their WORST?				
O None	O Mild	O Moderate	O Severe	O Very severe
b. In the last 7 days, how much did PROBLEMS WITH CONCENTRATION INTERFERE with your usual or daily activities?				
O Not at all	O A little bit	O Somewhat	O Quite a bit	O Very much

**47. PRO-CTCAE® Symptom Term:** Memory				
a. In the last 7 days, what was the SEVERITY of your PROBLEMS WITH MEMORY at their WORST?				
O None	O Mild	O Moderate	O Severe	O Very severe
b. In the last 7 days, how much did PROBLEMS WITH MEMORY INTERFERE with your usual or daily activities?				
O Not at all	O A little bit	O Somewhat	O Quite a bit	O Very much

**48. PRO-CTCAE® Symptom Term:** General pain				
a. In the last 7 days, how OFTEN did you have PAIN?				
O Never	O Rarely	O Occasionally	O Frequently	O Almost constantly
b. In the last 7 days, what was the SEVERITY of your PAIN at its WORST?				
O None	O Mild	O Moderate	O Severe	O Very severe
c. In the last 7 days, how much did PAIN INTERFERE with your usual or daily activities?				
O Not at all	O A little bit	O Somewhat	O Quite a bit	O Very much

**49. PRO-CTCAE® Symptom Term:** Headache				
a. In the last 7 days, how OFTEN did you have a HEADACHE?				
O Never	O Rarely	O Occasionally	O Frequently	O Almost constantly
b. In the last 7 days, what was the SEVERITY of your HEADACHE at its WORST?				
O None	O Mild	O Moderate	O Severe	O Very severe
c. In the last 7 days, how much did your HEADACHE INTERFERE with your usual or daily activities?				
O Not at all	O A little bit	O Somewhat	O Quite a bit	O Very much

The PRO-CTCAE® items and information herein were developed by the Division of Cancer Control and Population Sciences in the NATIONAL CANCER INSTITUTE at the NATIONAL INSTITUTES OF HEALTH, in Bethesda, Maryland, U.S.A. Use of the PRO-CTCAE® is subject to NCI's Terms of Use.

**50. PRO-CTCAE® Symptom Term:** Muscle pain				
a. In the last 7 days, how OFTEN did you have ACHING MUSCLES?				
O Never	O Rarely	O Occasionally	O Frequently	O Almost constantly
b. In the last 7 days, what was the SEVERITY of your ACHING MUSCLES at their WORST?				
O None	O Mild	O Moderate	O Severe	O Very severe
c. In the last 7 days, how much did ACHING MUSCLES INTERFERE with your usual or daily activities?				
O Not at all	O A little bit	O Somewhat	O Quite a bit	O Very much

**51. PRO-CTCAE® Symptom Term:** Joint pain				
a. In the last 7 days, how OFTEN did you have ACHING JOINTS (SUCH AS ELBOWS, KNEES, SHOULDERS)?				
O Never	O Rarely	O Occasionally	O Frequently	O Almost constantly
b. In the last 7 days, what was the SEVERITY of your ACHING JOINTS (SUCH AS ELBOWS, KNEES, SHOULDERS) at their WORST?				
O None	O Mild	O Moderate	O Severe	O Very severe
c. In the last 7 days, how much did ACHING JOINTS (SUCH AS ELBOWS, KNEES, SHOULDERS) INTERFERE with your usual or daily activities?				
O Not at all	O A little bit	O Somewhat	O Quite a bit	O Very much

**52. PRO-CTCAE® Symptom Term:** Insomnia				
a. In the last 7 days, what was the SEVERITY of your INSOMNIA (INCLUDING DIFFICULTY FALLING ASLEEP, STAYING ASLEEP, OR WAKING UP EARLY) at its WORST?				
O None	O Mild	O Moderate	O Severe	O Very severe
b. In the last 7 days, how much did INSOMNIA (INCLUDING DIFFICULTY FALLING ASLEEP, STAYING ASLEEP, OR WAKING UP EARLY) INTERFERE with your usual or daily activities?				
O Not at all	O A little bit	O Somewhat	O Quite a bit	O Very much

The PRO-CTCAE® items and information herein were developed by the Division of Cancer Control and Population Sciences in the NATIONAL CANCER INSTITUTE at the NATIONAL INSTITUTES OF HEALTH, in Bethesda, Maryland, U.S.A. Use of the PRO-CTCAE® is subject to NCI's Terms of Use.

**53. PRO-CTCAE® Symptom Term:** Fatigue				
a. In the last 7 days, what was the SEVERITY of your FATIGUE, TIREDNESS, OR LACK OF ENERGY at its WORST?				
O None	O Mild	O Moderate	O Severe	O Very severe
b. In the last 7 days, how much did FATIGUE, TIREDNESS, OR LACK OF ENERGY INTERFERE with your usual or daily activities?				
O Not at all	O A little bit	O Somewhat	O Quite a bit	O Very much

**54. PRO-CTCAE® Symptom Term:** Anxious				
a. In the last 7 days, how OFTEN did you feel ANXIETY?				
O Never	O Rarely	O Occasionally	O Frequently	O Almost constantly
b. In the last 7 days, what was the SEVERITY of your ANXIETY at its WORST?				
O None	O Mild	O Moderate	O Severe	O Very severe
c. In the last 7 days, how much did ANXIETY INTERFERE with your usual or daily activities?				
O Not at all	O A little bit	O Somewhat	O Quite a bit	O Very much

**55. PRO-CTCAE® Symptom Term:** Discouraged				
a. In the last 7 days, how OFTEN did you FEEL THAT NOTHING COULD CHEER YOU UP?				
O Never	O Rarely	O Occasionally	O Frequently	O Almost constantly
b. In the last 7 days, what was the SEVERITY of your FEELINGS THAT NOTHING COULD CHEER YOU UP at their WORST?				
O None	O Mild	O Moderate	O Severe	O Very severe
c. In the last 7 days, how much did FEELING THAT NOTHING COULD CHEER YOU UP INTERFERE with your usual or daily activities?				
O Not at all	O A little bit	O Somewhat	O Quite a bit	O Very much

The PRO-CTCAE® items and information herein were developed by the Division of Cancer Control and Population Sciences in the NATIONAL CANCER INSTITUTE at the NATIONAL INSTITUTES OF HEALTH, in Bethesda, Maryland, U.S.A. Use of the PRO-CTCAE® is subject to NCI's Terms of Use.

**56. PRO-CTCAE® Symptom Term:** Sad				
a. In the last 7 days, how OFTEN did you have SAD OR UNHAPPY FEELINGS?				
O Never	O Rarely	O Occasionally	O Frequently	O Almost constantly
b. In the last 7 days, what was the SEVERITY of your SAD OR UNHAPPY FEELINGS at their WORST?				
O None	O Mild	O Moderate	O Severe	O Very severe
c. In the last 7 days, how much did SAD OR UNHAPPY FEELINGS INTERFERE with your usual or daily activities?				
O Not at all	O A little bit	O Somewhat	O Quite a bit	O Very much

**57. PRO-CTCAE® Symptom Term:** Irregular periods/vaginal bleeding		
a. In the last 7 days, did you have any IRREGULAR MENSTRUAL PERIODS?		
O Yes	O No	O Not applicable

**58. PRO-CTCAE® Symptom Term:** Missed expected menstrual period		
a. In the last 7 days, did you MISS AN EXPECTED MENSTRUAL PERIOD?		
O Yes	O No	O Not applicable

**59. PRO-CTCAE® Symptom Term:** Vaginal discharge				
a. In the last 7 days, did you have any UNUSUAL VAGINAL DISCHARGE?				
O Not at all	O A little bit	O Somewhat	O Quite a bit	O Very much

**60. PRO-CTCAE® Symptom Term:** Vaginal dryness				
a. In the last 7 days, what was the SEVERITY of your VAGINAL DRYNESS at its WORST?				
O None	O Mild	O Moderate	O Severe	O Very severe

The PRO-CTCAE® items and information herein were developed by the Division of Cancer Control and Population Sciences in the NATIONAL CANCER INSTITUTE at the NATIONAL INSTITUTES OF HEALTH, in Bethesda, Maryland, U.S.A. Use of the PRO-CTCAE® is subject to NCI's Terms of Use.

**61. PRO-CTCAE® Symptom Term:** Painful urination				
a. In the last 7 days, what was the SEVERITY of your PAIN OR BURNING WITH URINATION at its WORST?				
O None	O Mild	O Moderate	O Severe	O Very severe

**62. PRO-CTCAE® Symptom Term:** Urinary urgency				
a. In the last 7 days, how OFTEN did you feel an URGE TO URINATE ALL OF A SUDDEN?				
O Never	O Rarely	O Occasionally	O Frequently	O Almost constantly
b. In the last 7 days, how much did SUDDEN URGES TO URINATE INTERFERE with your usual or daily activities?				
O Not at all	O A little bit	O Somewhat	O Quite a bit	O Very much

**63. PRO-CTCAE® Symptom Term:** Urinary frequency				
a. In the last 7 days, were there times when you had to URINATE FREQUENTLY?				
O Never	O Rarely	O Occasionally	O Frequently	O Almost constantly
b. In the last 7 days, how much did FREQUENT URINATION INTERFERE with your usual or daily activities?				
O Not at all	O A little bit	O Somewhat	O Quite a bit	O Very much

**64. PRO-CTCAE® Symptom Term:** Change in usual urine color	
a. In the last 7 days, did you have any URINE COLOR CHANGE?	
O Yes	O No

**65. PRO-CTCAE® Symptom Term:** Urinary incontinence				
a. In the last 7 days, how OFTEN did you have LOSS OF CONTROL OF URINE (LEAKAGE)?				
O Never	O Rarely	O Occasionally	O Frequently	O Almost constantly
b. In the last 7 days, how much did LOSS OF CONTROL OF URINE (LEAKAGE) INTERFERE with your usual or daily activities?				
O Not at all	O A little bit	O Somewhat	O Quite a bit	O Very much

The PRO-CTCAE® items and information herein were developed by the Division of Cancer Control and Population Sciences in the NATIONAL CANCER INSTITUTE at the NATIONAL INSTITUTES OF HEALTH, in Bethesda, Maryland, U.S.A. Use of the PRO-CTCAE® is subject to NCI's Terms of Use.

66. **PRO-CTCAE® Symptom Term:** Achieve and maintain erection						
a. In the last 7 days, what was the SEVERITY of your DIFFICULTY GETTING OR KEEPING AN ERECTION at its WORST?						
O None	O Mild	O Moderate	O Severe	O Very severe	O Not sexually active	O Prefer not to answer

67. **PRO-CTCAE® Symptom Term:** Ejaculation						
a. In the last 7 days, how OFTEN did you have EJACULATION PROBLEMS?						
O Never	O Rarely	O Occasionally	O Frequently	O Almost constantly	O Not sexually active	O Prefer not to answer

68. **PRO-CTCAE® Symptom Term:** Decreased libido						
a. In the last 7 days, what was the SEVERITY of your DECREASED SEXUAL INTEREST at its WORST?						
O None	O Mild	O Moderate	O Severe	O Very severe	O Not sexually active	O Prefer not to answer

69. **PRO-CTCAE® Symptom Term:** Delayed orgasm			
a. In the last 7 days, did you feel that it TOOK TOO LONG TO HAVE AN ORGASM OR CLIMAX?			
O Yes	O No	O Not sexually active	O Prefer not to answer

70. **PRO-CTCAE® Symptom Term:** Unable to have orgasm			
a. In the last 7 days, were you UNABLE TO HAVE AN ORGASM OR CLIMAX?			
O Yes	O No	O Not sexually active	O Prefer not to answer

71. **PRO-CTCAE® Symptom Term:** Pain w/sexual intercourse						
a. In the last 7 days, what was the SEVERITY of your PAIN DURING VAGINAL SEX at its WORST?						
O None	O Mild	O Moderate	O Severe	O Very severe	O Not sexually active	O Prefer not to answer

The PRO-CTCAE® items and information herein were developed by the Division of Cancer Control and Population Sciences in the NATIONAL CANCER INSTITUTE at the NATIONAL INSTITUTES OF HEALTH, in Bethesda, Maryland, U.S.A. Use of the PRO-CTCAE® is subject to NCI's Terms of Use.

72. **PRO-CTCAE® Symptom Term:** Breast swelling and tenderness				
a. In the last 7 days, what was the SEVERITY of your BREAST AREA ENLARGEMENT OR TENDERNESS at its WORST?				
O None	O Mild	O Moderate	O Severe	O Very severe

73. **PRO-CTCAE® Symptom Term:** Bruising	
a. In the last 7 days, did you BRUISE EASILY (BLACK AND BLUE MARKS)?	
O Yes	O No

74. **PRO-CTCAE® Symptom Term:** Chills				
a. In the last 7 days, how OFTEN did you have SHIVERING OR SHAKING CHILLS?				
O Never	O Rarely	O Occasionally	O Frequently	O Almost constantly
b. In the last 7 days, what was the SEVERITY of your SHIVERING OR SHAKING CHILLS at their WORST?				
O None	O Mild	O Moderate	O Severe	O Very severe

75. **PRO-CTCAE® Symptom Term:** Increased sweating				
a. In the last 7 days, how OFTEN did you have UNEXPECTED OR EXCESSIVE SWEATING DURING THE DAY OR NIGHTIME (NOT RELATED TO HOT FLASHES/FLUSHES)?				
O Never	O Rarely	O Occasionally	O Frequently	O Almost constantly
b. In the last 7 days, what was the SEVERITY of your UNEXPECTED OR EXCESSIVE SWEATING DURING THE DAY OR NIGHTTIME (NOT RELATED TO THE HOT FLASHES/FLUSHES) at its WORST?				
O None	O Mild	O Moderate	O Severe	O Very severe

76. **PRO-CTCAE® Symptom Term:** Decreased sweating	
a. In the last 7 days, did you have an UNEXPECTED DECREASE IN SWEATING?	
O Yes	O No

The PRO-CTCAE® items and information herein were developed by the Division of Cancer Control and Population Sciences in the NATIONAL CANCER INSTITUTE at the NATIONAL INSTITUTES OF HEALTH, in Bethesda, Maryland, U.S.A. Use of the PRO-CTCAE® is subject to NCI's Terms of Use.

**77. PRO-CTCAE® Symptom Term:** Hot flashes				
a. In the last 7 days, how OFTEN did you have HOT FLASHES/FLUSHES?				
O Never	O Rarely	O Occasionally	O Frequently	O Almost constantly
b. In the last 7 days, what was the SEVERITY of your HOT FLASHES/FLUSHES at their WORST?				
O None	O Mild	O Moderate	O Severe	O Very severe

**78. PRO-CTCAE® Symptom Term:** Nosebleed				
a. In the last 7 days, how OFTEN did you have NOSEBLEEDS?				
O Never	O Rarely	O Occasionally	O Frequently	O Almost constantly
b. In the last 7 days, what was the SEVERITY of your NOSEBLEEDS at their WORST?				
O None	O Mild	O Moderate	O Severe	O Very severe

**79. PRO-CTCAE® Symptom Term:** Pain and swelling at injection site		
a. In the last 7 days, did you HAVE ANY PAIN, SWELLING, OR REDNESS AT A SITE OF DRUG INJECTION OR IV?		
O Yes	O No	O Not applicable

**80. PRO-CTCAE® Symptom Term:** Body odor				
a. In the last 7 days, what was the SEVERITY of your BODY ODOR at its WORST?				
O None	O Mild	O Moderate	O Severe	O Very severe

The PRO-CTCAE® items and information herein were developed by the Division of Cancer Control and Population Sciences in the NATIONAL CANCER INSTITUTE at the NATIONAL INSTITUTES OF HEALTH, in Bethesda, Maryland, U.S.A. Use of the PRO-CTCAE® is subject to NCI's Terms of Use.

NCI- PRO-CTCAE® ITEMS-ENGLISH (UNIVERSAL)

Item Library Version 1.0

**OTHER SYMPTOMS**	
Do you have any other symptoms that you wish to report?	
O Yes	O No
**Please list any other symptoms:**	
1.	In the last 7 days, what was the SEVERITY of this symptom at its WORST?<br>O None O Mild O Moderate O Severe O Very Severe
2.	In the last 7 days, what was the SEVERITY of this symptom at its WORST?<br>O None O Mild O Moderate O Severe O Very Severe
3.	In the last 7 days, what was the SEVERITY of this symptom at its WORST?<br>O None O Mild O Moderate O Severe O Very Severe
4.	In the last 7 days, what was the SEVERITY of this symptom at its WORST?<br>O None O Mild O Moderate O Severe O Very Severe
5.	In the last 7 days, what was the SEVERITY of this symptom at its WORST?<br>O None O Mild O Moderate O Severe O Very Severe

The PRO-CTCAE® items and information herein were developed by the Division of Cancer Control and Population Sciences in the NATIONAL CANCER INSTITUTE at the NATIONAL INSTITUTES OF HEALTH, in Bethesda, Maryland, U.S.A. Use of the PRO-CTCAE® is subject to NCI's Terms of Use.
