# Supplementary material for: Financial toxicity on treatment outcomes in head & neck cancer patients undergoing radiation therapy
Source: Radiat Oncol. 2025 Nov 25;20:176. doi: 10.1186/s13014-025-02749-x (PMC12648789; doi:10.1186/s13014-025-02749-x)
Supplement: Supplementary file 4 — Supplementary material 4 [file 13014_2025_2749_MOESM4_ESM.docx]

**
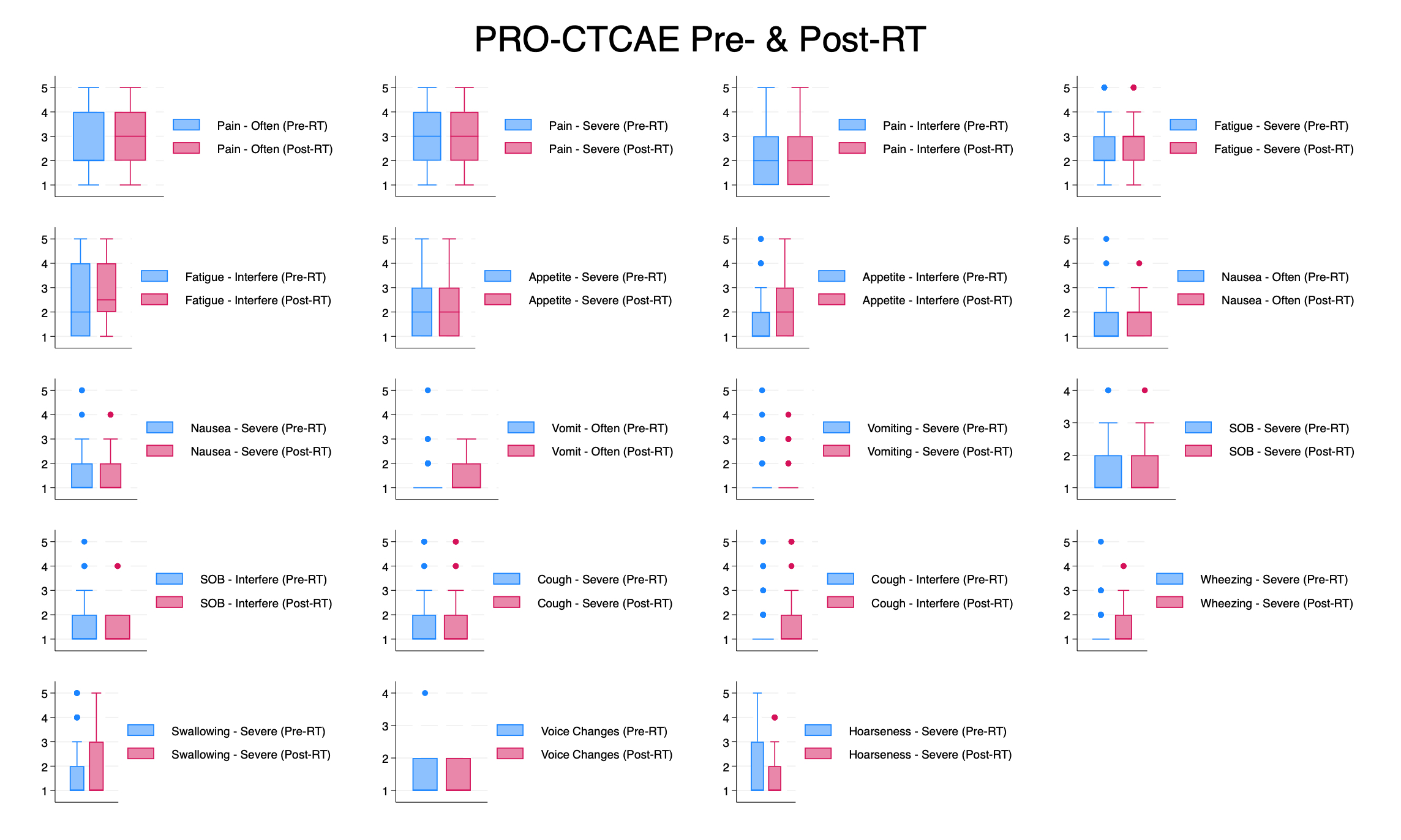
**

**SUPPLEMENTAL FIGURE 1.** Distribution of pre-radiation therapy (pre-RT) and post-radiation therapy (post-RT) patient-reported outcomes for common terminology criteria for adverse events (PRO-CTCAE). Higher scores indicate higher patient-reported toxicity outcomes.

**SUPPLEMENTAL FIGURE 2.** Kaplan Meier Curves for overall survival stratified by (A) pre-radiation therapy (pre-RT) grade 0 vs. grade 1 financial toxicity and (B) post-radiation therapy (post-RT) grade 0 vs. grade 1 financial toxicity.
